# Nitrone chemistry: a versatile gateway to diverse heterocycles

**DOI:** 10.1039/d5ra07748f

**Published:** 2026-01-05

**Authors:** Suhasini Mohapatra, Kamalika Prusty, Subhashree Bhol, Gopinatha Panigrahi, Sabita Nayak

**Affiliations:** a Organic Synthesis Laboratory, Department of Chemistry, Ravenshaw University Cuttack-753003 Odisha India sabitanayak18@gmail.com

## Abstract

Nitrones (azomethine *N*-oxides) are among the most versatile intermediates in organic synthesis, enabling the efficient construction of heterocyclic frameworks that underpin advances in medicinal chemistry, materials science, and chemical biology. Over the past few years, transition-metal-catalyzed strategies have delivered remarkable control over regio- and stereoselectivity, yet their cost and limitations in substrate scope have encouraged the search for alternatives. In this context, transition-metal-free protocols, including [3 + 2], [2 + 2] and [4 + 2] cycloadditions, have emerged as sustainable and economical approaches. Complementing these methods, new developments such as asymmetric click reactions, deoxygenative cyclizations, silylacetate-promoted addition reactions, photoredox catalysis, and self-oxidative cyclizations further broaden the synthetic toolbox, enabling access to structurally complex and biologically relevant scaffolds. By integrating these diverse methodologies, nitrone chemistry continues to evolve as a dynamic platform for heterocycle construction. This review highlights recent synthetic strategies for nitrone-derived heterocycles reported from 2021 to 2025, critically evaluating their advantages and limitations while outlining promising directions toward greener, more versatile, and practically useful methodologies.

## Abstract 2

A concise overview of recent advances (2021–2025) in nitrone chemistry, emphasizing metal-catalyzed and metal-free cycloadditions, emerging photoredox and asymmetric strategies, and their impact on constructing diverse heterocycles.

## Graphical abstract

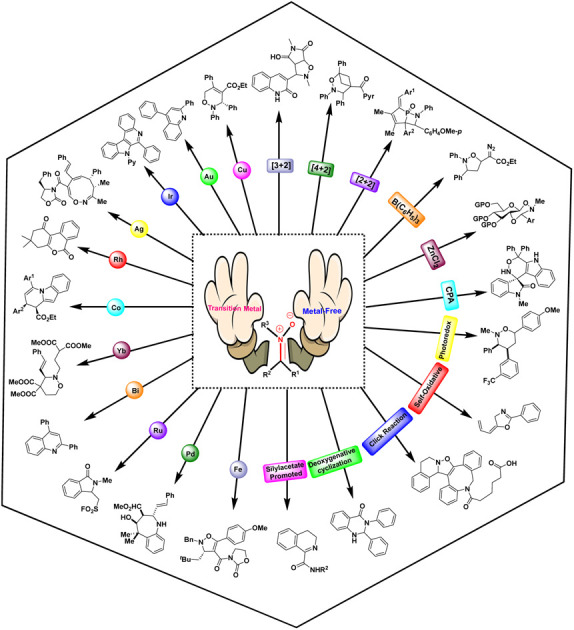

## 1 Introduction

Nitrones (azomethine *N*-oxides) are recognized as important molecules in medicinal chemistry, in addition to their applications in agrochemicals, polymers, and material science.^1–3^ Various nitrone derivatives have exhibited promising biological activities, including antioxidant, neuroprotective, anticancer, and antiviral effects. Furthermore, nitrones have been extensively explored as 1,3-dipoles in cycloaddition reactions, providing access to diverse biologically active heterocycles.^4,5^ Owing to this broad spectrum of utility, significant research efforts have been devoted to the synthesis of diverse nitrone frameworks.^6,7^

**Fig. 1:**
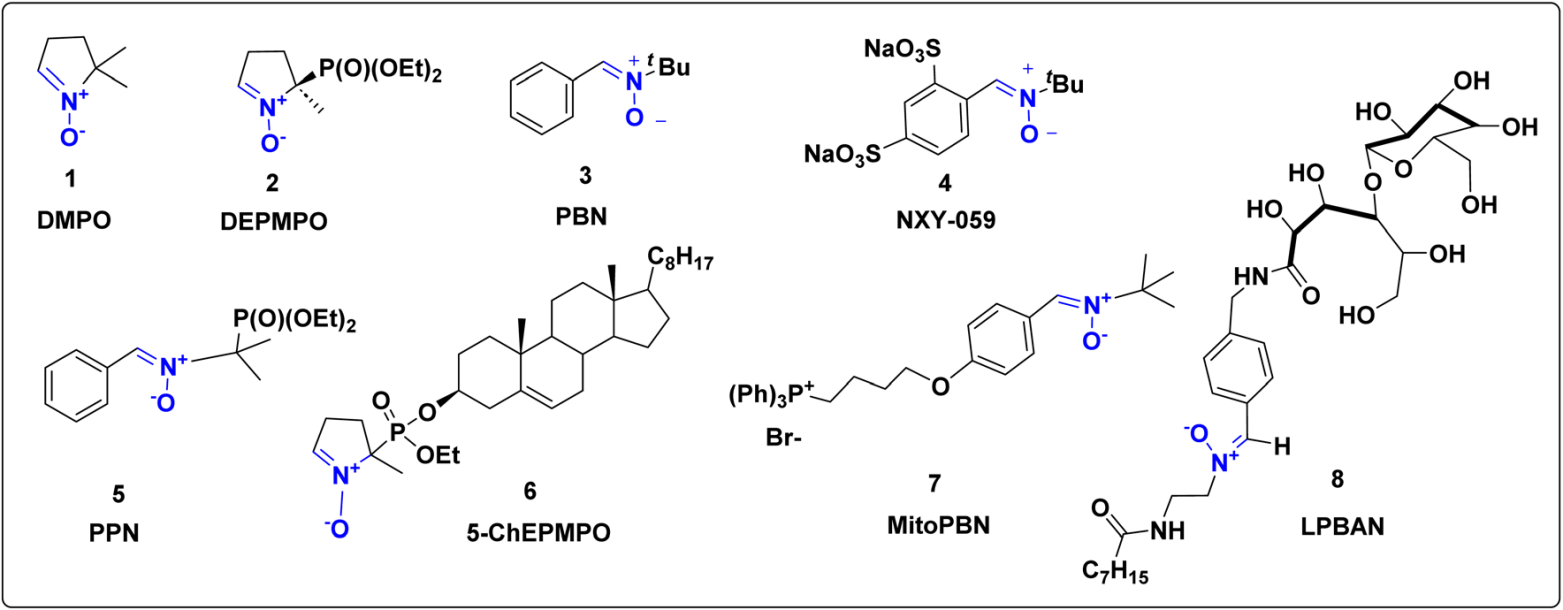
Nitrone based bioactive molecules.

Among them, α-phenyl-*N-tert*-butylnitrone (PBN) has emerged as the prototypical spin trap, exhibiting diverse pharmacological activities including neuroprotective, cardioprotective, and anti-teratogenic effects.^8–16^ Its water-soluble analog, disodium [(*tert*-butylimino)methyl]benzene-1,3-disulfonate *N*-oxide (NXY-059), advanced to phase III clinical trials as a neuroprotective drug, although its mechanism of action remains incompletely understood.^17^ Meanwhile, other nitrones such as 5,5-dimethyl-1-pyrroline *N*-oxide (DMPO) have been less extensively studied for pharmacological activity. To address limitations of poor stability and limited cellular uptake, structurally modified nitrones such as DEPMPO, PPN, 5-ChEPMO, MitoPBN, and LPBNAH (Fig. 1) have been developed with improved spin trapping properties and bioavailability.^18,19^ These advances highlight the dual importance of nitrones in both chemical and biomedical research.

**Fig. 2:**
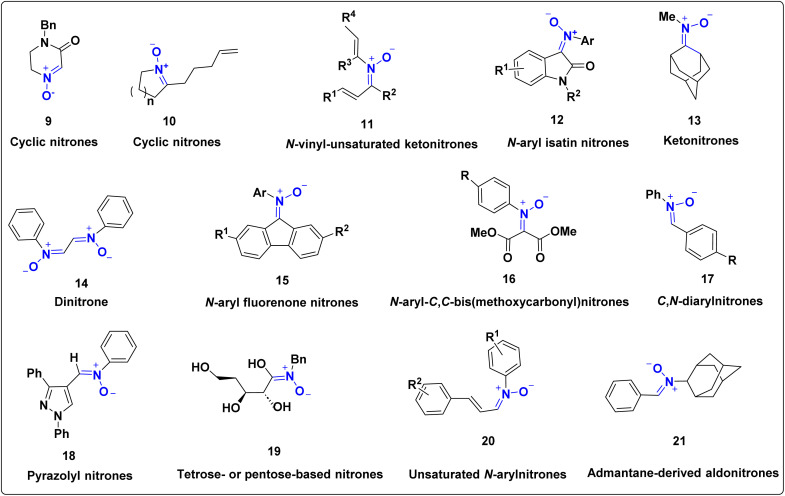
Various types of nitrones.

From a synthetic perspective, nitrones are easy to prepare and serve as versatile intermediates in cycloaddition chemistry. Their electronic structure allows them to react with alkenes, alkynes, and carbonyl groups, leading to a wide variety of heterocyclic products. Nitrones are best known for their classical [3 + 2] dipolar cycloadditions, which produce isoxazolidines and isoxazolines important five-membered heterocycles often found in natural products and pharmaceuticals.^20–28^ However, they can also participate in other reaction modes, including [2 + 2], [3 + 3], [4 + 1], and [4 + 2] cycloadditions, which expand their synthetic scope.^29^ The nature of the nitrone framework plays a crucial role in these reactions. Based on these structural classifications, the nitrone derivatives in Fig. 2 (compounds 9–21) highlight representative examples used widely in contemporary 1,3-dipolar cycloaddition chemistry. Compounds 9–10 represent cyclic nitrones derived from isoxazolidine or pyrroline scaffolds, whose conformational rigidity improves regio- and stereoselectivity in cycloaddition reactions.^30,31^ Compound 11 corresponds to an α,β-unsaturated ketonitrone, which exhibits enhanced electrophilicity and reacts efficiently with electron-rich dipolarophiles.^32,33^ Compounds 12–13 are *N*-aryl isatin nitrones and ketonitrones that serve as versatile precursors in the synthesis of spirocyclic and fused heterocyclic scaffolds.^34^ Compound 14 is a dinitrone derivative capable of undergoing sequential cycloaddition pathways, enabling rapid molecular complexity. Compounds 15–17 include *N*-aryl and *C*-aryl nitrones that allow electronic tuning and participate effectively under metal-free or organocatalytic conditions. Compounds 18–19 represent heteroaryl and carbohydrate-derived chiral nitrones that provide enantioselective access to stereo defined heterocycles. Finally, compounds 20–21 are unsaturated and adamantane-derived nitrones widely utilized in the construction of rigid polycyclic and biologically relevant frameworks.^6,31^

Recent advancements in nitrone chemistry have further broadened their synthetic potential, particularly in the context of metal-free and metal-catalyzed methodologies. Transition-metal catalysts such as Cu, Ag, Yb, and Ni have been employed to mediate regio- and stereoselective cycloaddition reactions under mild conditions with improved efficiency.^36–39^ In parallel, organocatalytic and Lewis's acid/base-promoted protocols have enabled greener and more sustainable approaches. These innovations facilitate the construction of not only simple five- and six-membered rings, but also complex, highly functionalized heterocycles with pharmaceutical relevance.^40,41^

In this review, we aim to provide a comprehensive overview of recent developments in nitrone-mediated synthesis of heterocyclic compounds. Special emphasis is placed on categorizing these methodologies based on the type of cycloaddition, the nature of catalysis (metal-free *vs.* metal-catalyzed), and the structural complexity of the products formed. Through this exploration, we seek to highlight the strategic value of nitrone chemistry in modern heterocyclic synthesis and its growing impact on the development of bioactive molecules. Herein, we have discussed in detail almost 47 publications from the last four years, *i.e.*, from 2021 to 2025.

### 1.1 Metal-catalyzed synthesis

Transition-metal catalysis has emerged as one of the most versatile and powerful strategies for nitrone transformations, enabling the construction of complex heterocyclic architectures with high efficiency, selectivity, and functional group tolerance. Transition metals such as Cu, Au, Ir, Ag, Pd, Co, Rh, Ru, Yb, Bi and Fe have been widely employed to mediate a variety of cycloadditions, annulations, and oxidative couplings involving nitrones.^35–39^ These catalytic systems often operate under mild conditions and allow access to frameworks that are otherwise difficult to achieve through metal-free or purely thermal processes. Moreover, transition-metal catalysts enable precise control over regio- and stereoselectivity, and in many cases facilitate cascade or multicomponent reactions that enhance step economy.^31^ Despite concerns regarding cost, toxicity, and metal residues, transition-metal catalysis continues to expand the synthetic scope of nitrone chemistry and plays a central role in the development of new methodologies for heterocycle synthesis and drug discovery.^42^

#### 1.1.1 Cu-Catalyzed

**Scheme 1:**
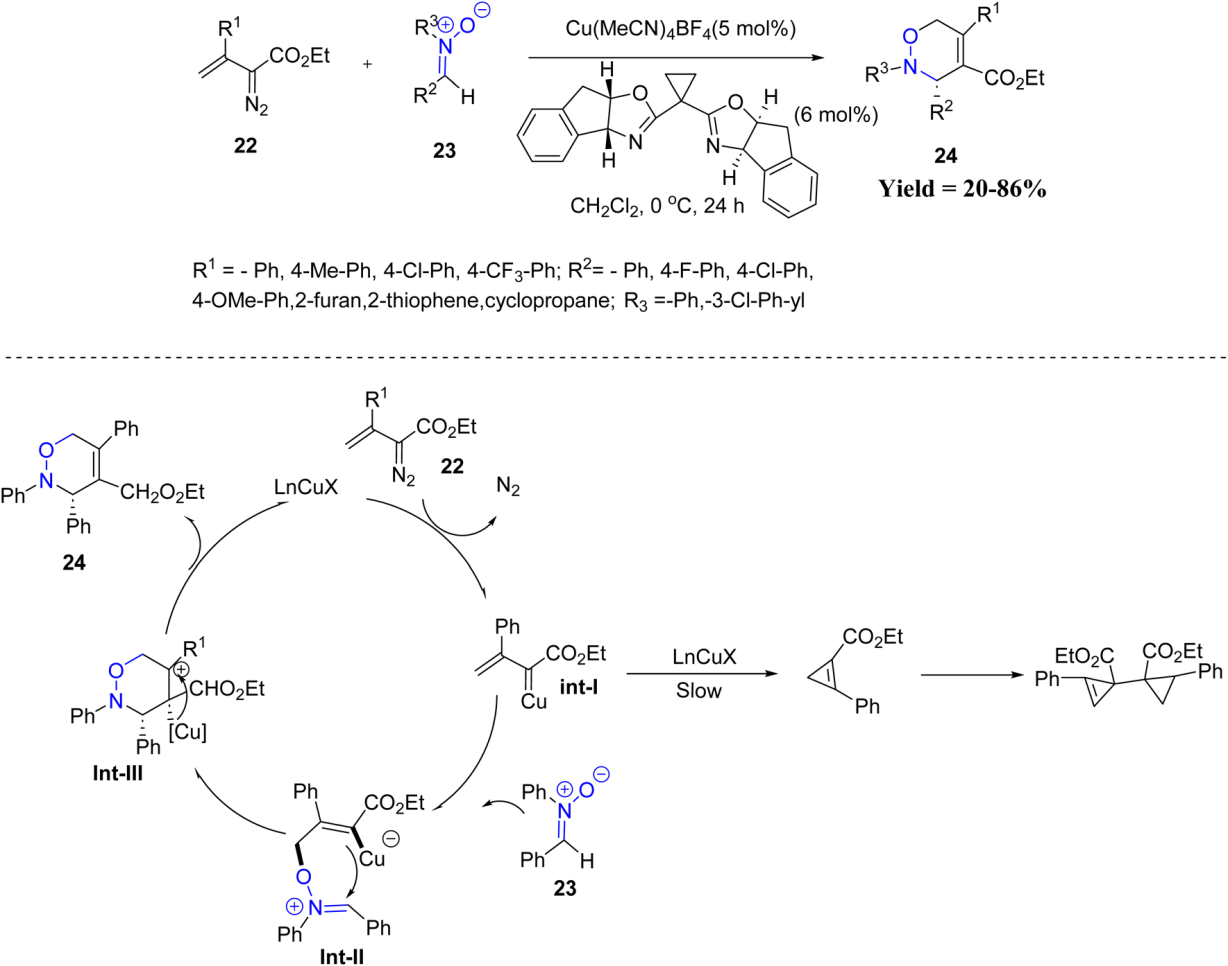
Cu(i)-Catalyzed [3 + 3] cycloaddition of β-aryl/alkyl vinyl diazoacetates with nitrones.

In this context, Michael P. Doyle and co-workers in 2021 reported that β-phenyl vinyl diazoacetate undergoes Rh_2_(OAc)_4_-catalyzed dimerization, and the resulting dimer was later applied as a ligand in the Cu(i)-catalyzed [3 + 3] cycloaddition of β-aryl and β-alkyl vinyl diazoacetates with nitrones. In this study, a clear reactivity trend was observed, where β-aryl substituted vinyl diazo compounds reacted more efficiently than β-alkyl analogues. Electron-donating groups on the aromatic ring improved both yield and enantioselectivity, while strong electron-withdrawing groups reduced the reactivity. He main limitations of the protocol include low yields for β-alkyl substrates, poor performance with *para*-CF_3_ substituted systems, and a lack of compatibility with certain highly electron-rich precursors. Under the optimized copper(i) catalytic system, the reaction furnished 3,6-dihydro-1,2-oxazine derivatives in good yields with excellent enantioselectivity under mild conditions. A plausible mechanism was proposed, wherein the copper(i) complex reacts with vinyl diazoacetate to generate a metallo–vinylcarbene intermediate (Int-I), which is trapped by the nitrone to afford intermediate (Int-II). Subsequent intramolecular cyclization of Int-II, followed by elimination of the copper(i) species, delivers the 3,6-dihydro-1,2-oxazine product (Scheme 1).^43^

**Scheme 2:**
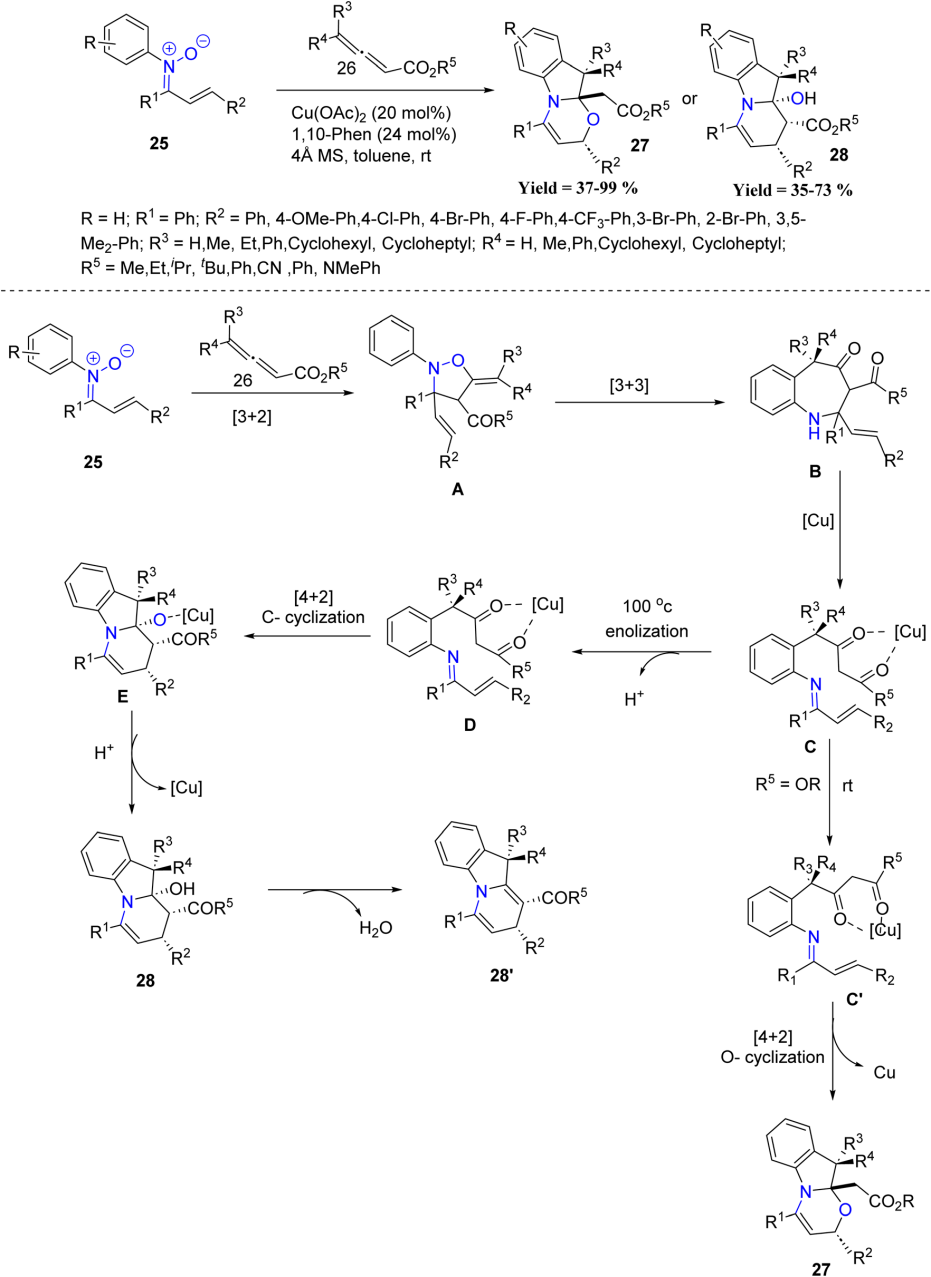
Copper(ii)-catalyzed cascade reactions of *N*-aryl nitrones and disubstituted allenoates.

Building upon the utility of nitrones in Cu-mediated transformations, Dong-Liang Mo *et al.* in 2023, reported a copper-catalyzed reaction between *N*-aryl nitrones and disubstituted allenoates for the synthesis of [1,3]oxazino[3,2-*a*]indolines and dihydropyrido[1,2-*a*]indolines in good to excellent yields with high diastereoselectivity. In this study, both electron-donating and electron-withdrawing substituents on the nitrone aryl ring reacted smoothly, giving comparable yields, indicating no strong electronic preference. Temperature had a clear influence on product outcome, where lower temperatures favoured formation of oxazinoindoline products, whereas higher temperatures promoted dehydration to give the corresponding dihydro-pyridoindolines. Although the method demonstrates a broad substrate scope and good functional group tolerance, several limitations were noted, including reduced yields with unsubstituted allenoates, formation of side products with ketone- or amide-substituted allenoates, longer reaction times for some substrates, and modest enantioselective induction. Mechanistically, nitrones first react with allenoates to form isooxazoline intermediate A, which undergoes [3,3]-sigmatropic rearrangement to yield azepinone B. Copper catalysis then promotes a *retro*-Mannich reaction, generating imine intermediate C. At low temperature, C adopts conformer C′, which proceeds through oxa-[4 + 2] cycloaddition to afford oxazinoindolines 27. Alternatively, enolization of C gives intermediate D, which undergoes C-[4 + 2] cycloaddition to form E, followed by protonolysis to give compound 28. Dehydration of 28 subsequently yields dihydropyridoindolines 28′. The high diastereoselectivity is attributed to copper(ii) coordination, which controls the stereochemistry of key intermediates during the cycloaddition steps (Scheme 2).^44^

**Scheme 3:**
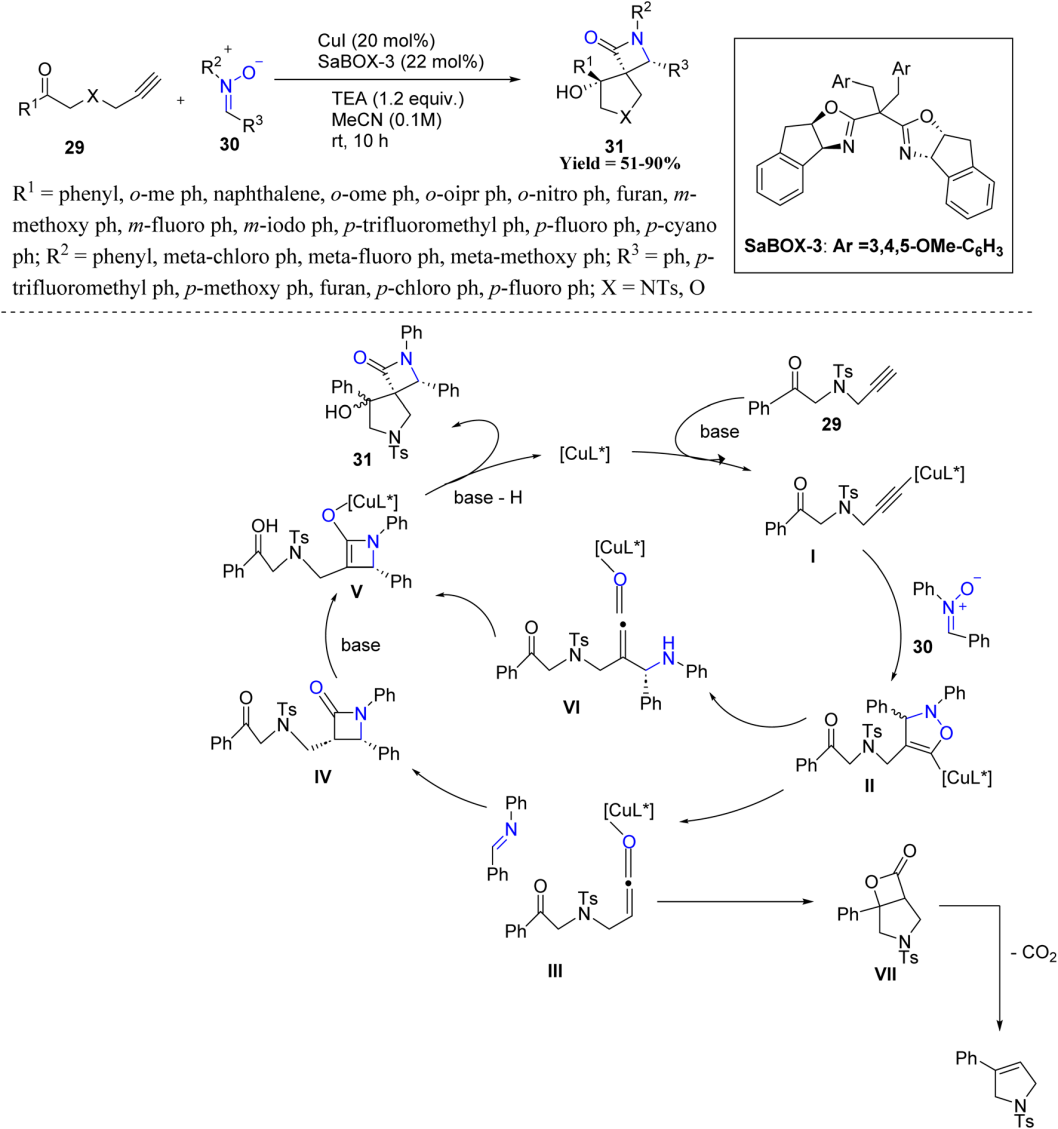
Copper-catalyzed Kinugasa/aldol domino reaction of alkyne-tethered ketones and nitrones.

In a related vein, Mark Lautens and co-workers in 2023 designed an enantioselective copper-catalyzed Kinugasa/aldol domino reaction that provides efficient access to structurally complex spirocyclic β-lactam pyrrolidinones 31. This innovative approach enables the stereoselective construction of two spirofused ring systems bearing three contiguous stereocenters, all under mild reaction conditions. Reactivity trends reveal that both steric and electronic effects of aryl ketones and nitrones strongly influence diastereo- and enantioselectivity. *Ortho*-methyl and methoxy ketones improved diastereoselectivity, whereas bulkier substituents like ^i^Pr reduced it. Electron-rich *ortho*- and *meta*-substituents generally enhanced enantioselectivity, while *para*-electron-withdrawing groups (*e.g.*, CF_3_) increased diastereoselectivity. Electron-rich or electron-deficient aryl nitrones were well-tolerated, whereas alkyl-substituted nitrones, oxygen-tethered ketones, and alkyl ketones showed poor stereocontrol or indeterminate enantiomeric ratios, highlighting limitations in substrate scope. Mechanistically, the reaction begins with a formal [3 + 2] cycloaddition between a terminal alkyne and a nitrone, facilitated by a Cu(i) catalyst and base, yielding an initial cycloadduct. A subsequent retro-[3 + 2] fragmentation generates a ketene and an imine intermediate. These species undergo a copper-mediated asymmetric [2 + 2] cycloaddition to form a β-lactam, which then undergoes a base-promoted, diastereoselective intramolecular aldol cyclization, forming the final spirocyclic product 31 (Scheme 3).^45^

**Scheme 4:**
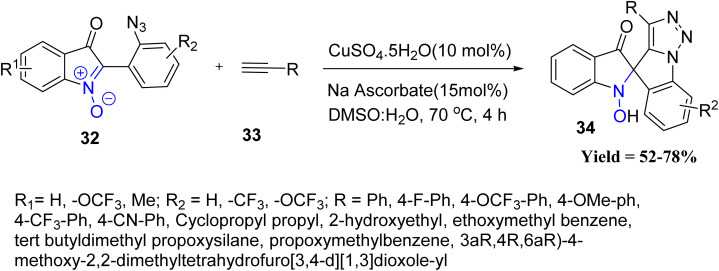
Cu-Catalyzed cycloaddition reaction of azido alkyne and nitrone group.

More recently, Chepuri V. Ramana and co-workers developed an efficient strategy for the construction of 1,2,3-triazole-fused spirocyclic frameworks 34. The methodology is based on a copper-catalyzed azide–alkyne cycloaddition (CuAAC), in which an appropriately positioned nitrone group effectively intercepts the reactive triazolide intermediate. This intramolecular trapping event enables the straightforward assembly of unprecedented spiro-polyheterocyclic scaffolds. The reaction tolerates a wide range of azido–isatogens and terminal alkynes, including aryl, cyclopropyl, and aliphatic substituents, delivering products in moderate to good yields. Protected alcohols and cyclopropyl groups were compatible, and even ribose-derived alkynes furnished products, albeit as diastereomeric mixtures. Mechanistically, the transformation proceeds *via* a Cu-catalyzed [3 + 2] cycloaddition of (2-azidoaryl) isatogen 32 with a terminal alkyne 33, followed by intramolecular capture of the Cu-triazolide by the isatogen. The reaction sequence results in the formation of one C–C and two C–N bonds, leading to a spiro-annulated heterocyclic framework 34. Limitations include modest yields with certain functional groups, difficulty in reducing the N–OH bond under mild conditions, and partial diastereoselectivity with more complex alkynes, indicating restricted substrate scope and functional group tolerance (Scheme 4).^46^

#### 1.1.2 Au-Catalyzed

**Scheme 5:**
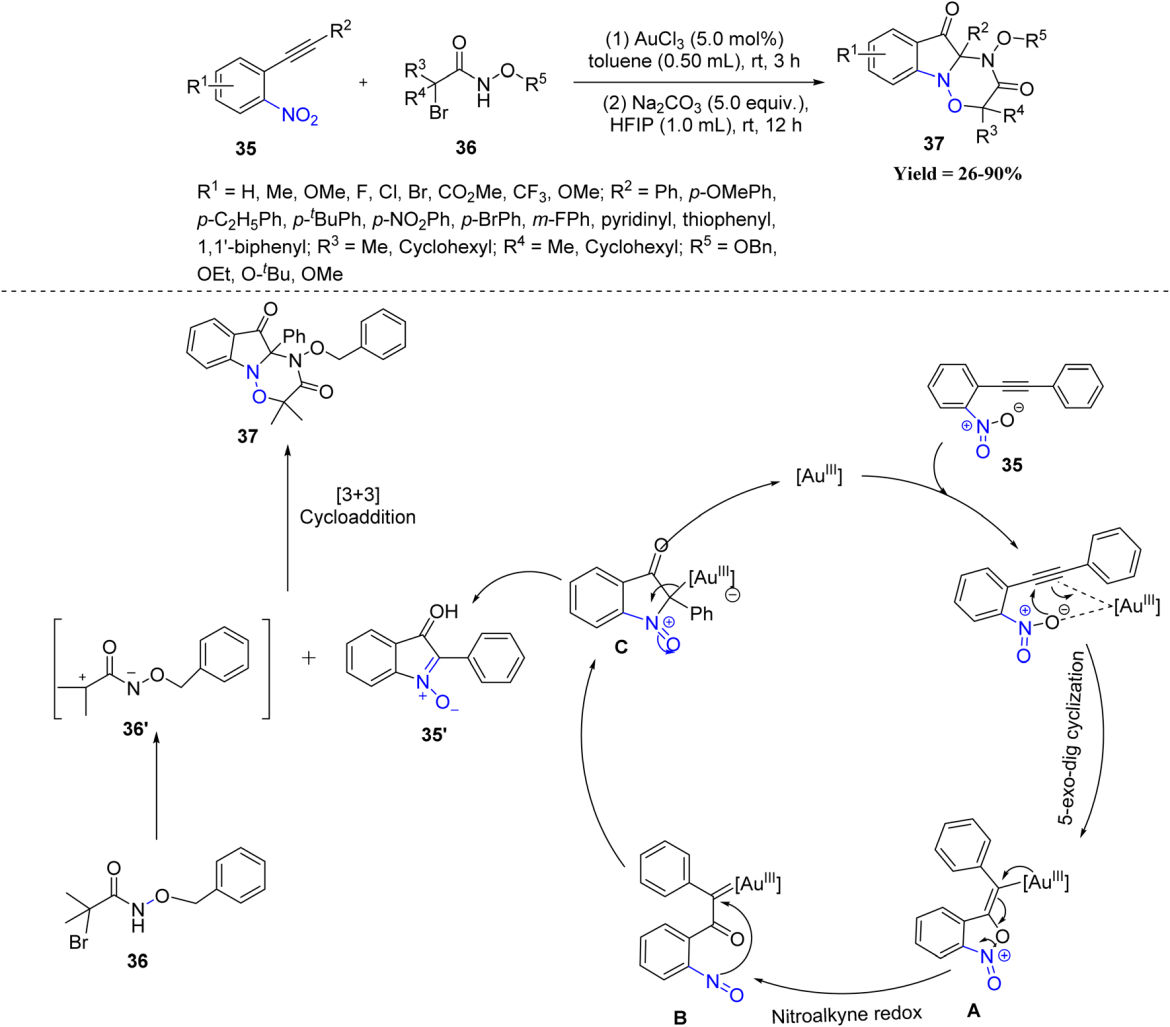
Au catalyzed [3 + 3] cycloaddition reaction of isatogens with azaoxyallyl cations.

An early advance in this area was reported by Xiuling Cui and co-workers in 2021, who reported a formal [3 + 3] cycloaddition between azaoxyallyl cations and isatogens generated *in situ* from α-halo hydroxamates 36 and *o*-nitroalkynes 35. The process involved cycloisomerization of *o*-nitroalkynes, base-mediated elimination, and subsequent dipolar cycloaddition, providing access to tricyclic fused indolin-3-ones 37 with excellent functional group tolerance (Scheme 5). Most *o*-nitroalkynes worked well, and the yields did not change much with different groups, even bulky ones, although nitro and some halogen groups gave slightly lower yields. The biggest limitations came from the α-halo hydroxamates, because monoalkyl and trichloro types did not react, and replacing the *N*-alkoxy group completely inhibited product formation, establishing the necessity of the *N*-alkoxy functionality. Importantly, the products exhibited strong DNA-binding fluorescence, highlighting their potential in biological imaging applications.^47^

**Scheme 6:**
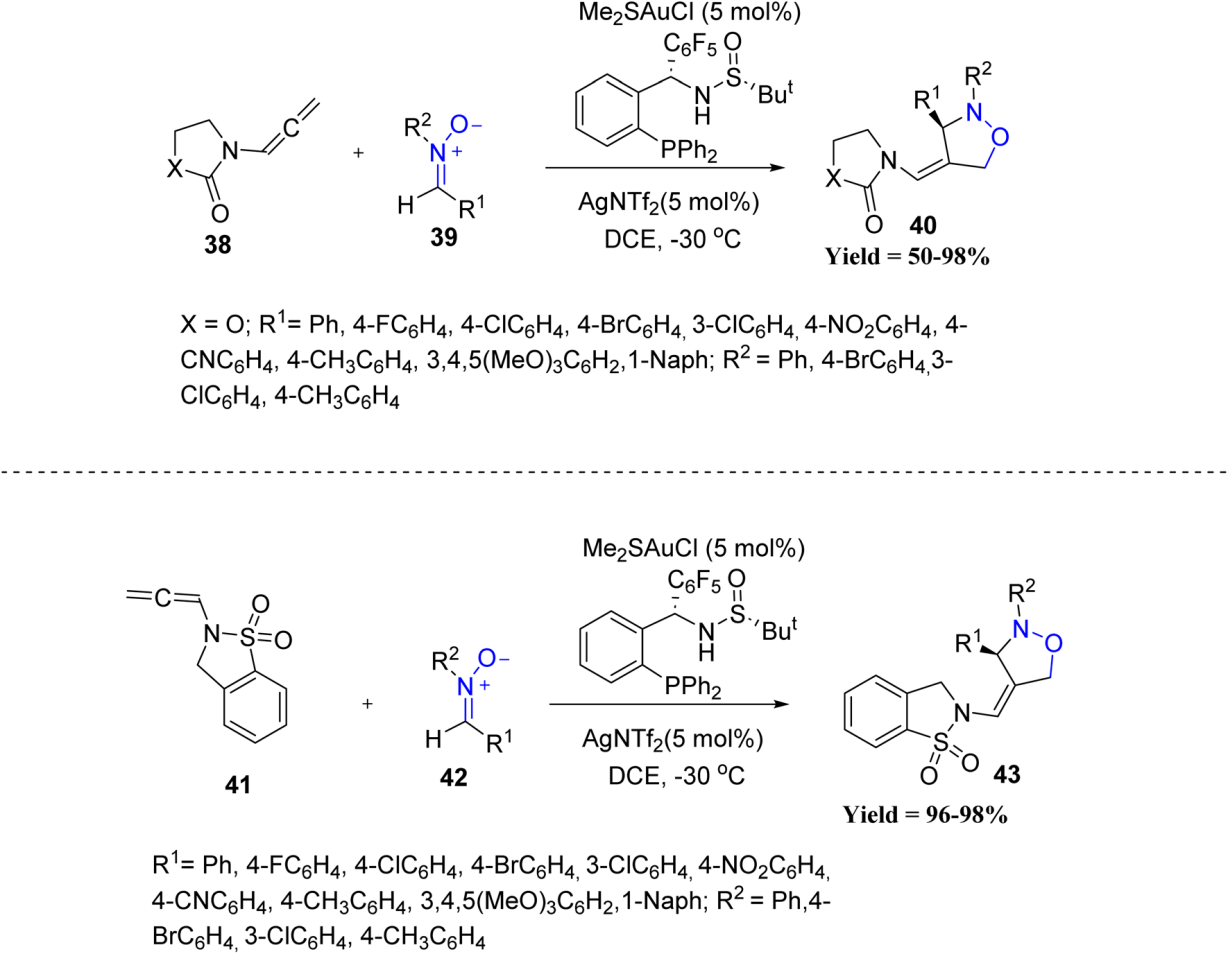
Gold-catalyzed intermolecular [3 + 2] cycloaddition of *N*-allenamides with nitrones.

Junliang Zhang *et al.* in 2022 expanded the synthetic toolbox by developing a highly enantioselective Au-catalyzed [3 + 2] cycloaddition of *N*-allenamides 38 with nitrones 39 using the chiral ligand Ming-Phos M6 (Scheme 6). Careful optimization of the chiral ligand revealed clear structure selectivity relationships. High enantioselectivity was obtained only when the ligand contained both a pentafluorophenyl substituent and a free sulfinamide N–H, demonstrating that these elements are essential for efficient asymmetric induction. In contrast, fluorine-modified or *N*-methylated variants resulted in a pronounced decrease in enantioselectivity, confirming the sensitivity of the system to subtle ligand modifications. However, tosyl-derived allenamides afforded products with lower enantioselectivity, and substrates outside heterocyclic *N*-allenamides were not tolerated, indicating limitations in broader substrate diversity.^48^

**Scheme 7:**
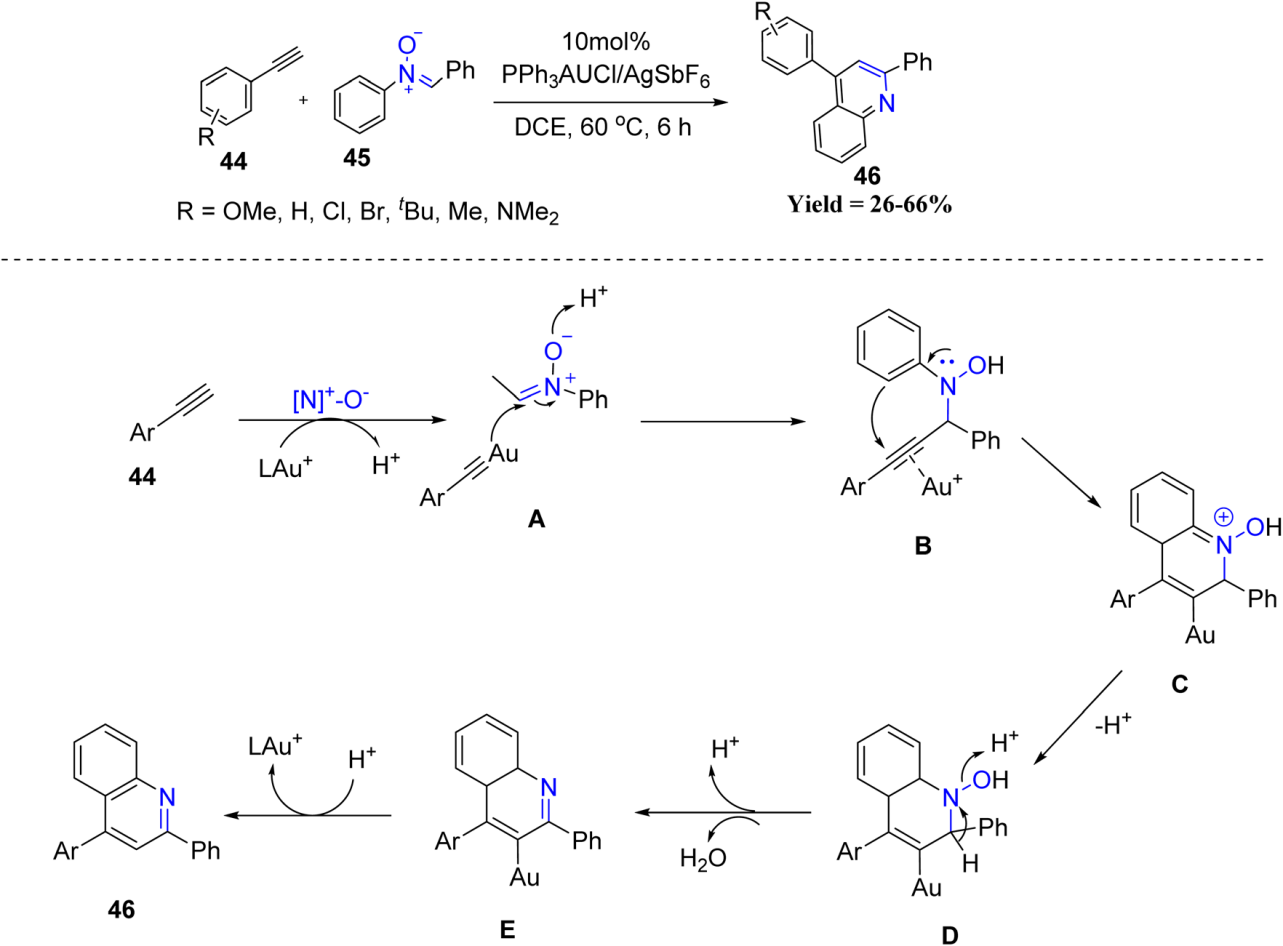
Gold-catalyzed [4 + 2] annulation between terminal arylynes and nitrones.

In the same year, Rai-Shung Liu *et al.* in 2022 disclosed a gold-catalyzed [4 + 2] annulation of terminal arylalkynes 44 with nitrones 45, affording quinoline derivatives 46 (Scheme 7). Initially, the terminal alkyne coordinates with a cationic gold(i) catalyst in the presence of an oxidizing additive, such as pyridine *N*-oxide, to generate a reactive alkynyl gold species A. This intermediate is known to exhibit enhanced nucleophilicity at the α-position and readily undergoes nucleophilic addition to the electrophilic carbon of the nitrone, forming a propargylic-type intermediate B. Subsequent intramolecular cyclization, likely proceeding *via* a 6-*endo*-dig pathway, leads to the construction of a six-membered dihydroquinoline framework E. The resulting intermediate then undergoes protodeauration to eliminate the gold catalyst and furnish the dihydroquinoline product 46. Electron-rich arylalkynes provided higher yields than electron-neutral or electron-withdrawing substrates. The reaction was also compatible with a range of substituted nitrones, although *meta*-substituted nitrones produced mixtures of regioisomers. Despite its broad applicability, the method showed reduced efficiency with electron-deficient alkynes, did not tolerate aliphatic alkynes, and exhibited regioselectivity limitations with sterically or electronically biased nitrones, defining boundaries in substrate scope (Scheme 7).^49^

**Scheme 8:**
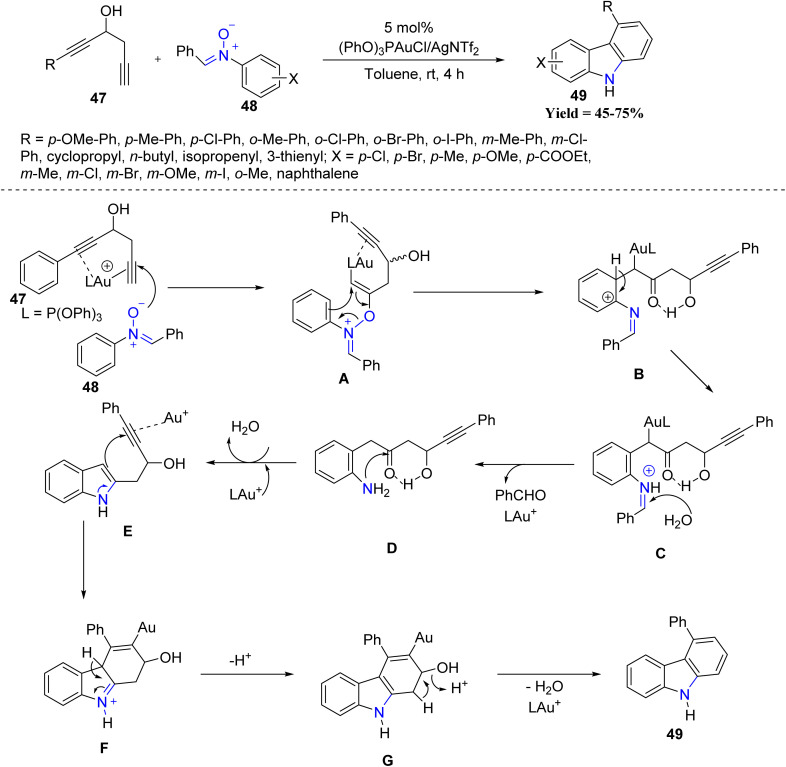
Au-Catalyzed [3 + 2] annulation cascades between 1,5-diyn-3-ol and nitrone.

Further innovation came from the same group in 2023, when they devised a cascade annulation of 1,5-diyn-3-ols 47 with nitrones 48 to furnish carbazole frameworks 49 (Scheme 8). The transformation begins with coordination of the gold catalyst to the alkyne, generating alkenyl–gold species A. This intermediate undergoes a 3,3-sigmatropic rearrangement to give species B, which upon proton loss produces an iminium cation C. Subsequent hydrolysis of C yields an aniline bearing a ketone functionality (intermediate D), which undergoes intramolecular indole/alkyne cyclization to form species F. Finally, dehydration of intermediate G delivers the carbazole product 49. This study demonstrates that gold catalysis can efficiently promote cascade processes for the rapid assembly of complex heterocycles. *Para*-substituted nitrones showed the highest reactivity, whereas *meta*-substituted substrates gave regioisomeric mixtures, and *ortho*-substituted nitrones afforded low yields. A limitation of the protocol is that substrates outside the 1,5-diyn-3-ol framework or lacking the internal alkyne functionality failed to undergo annulation.^50^

**Scheme 9:**
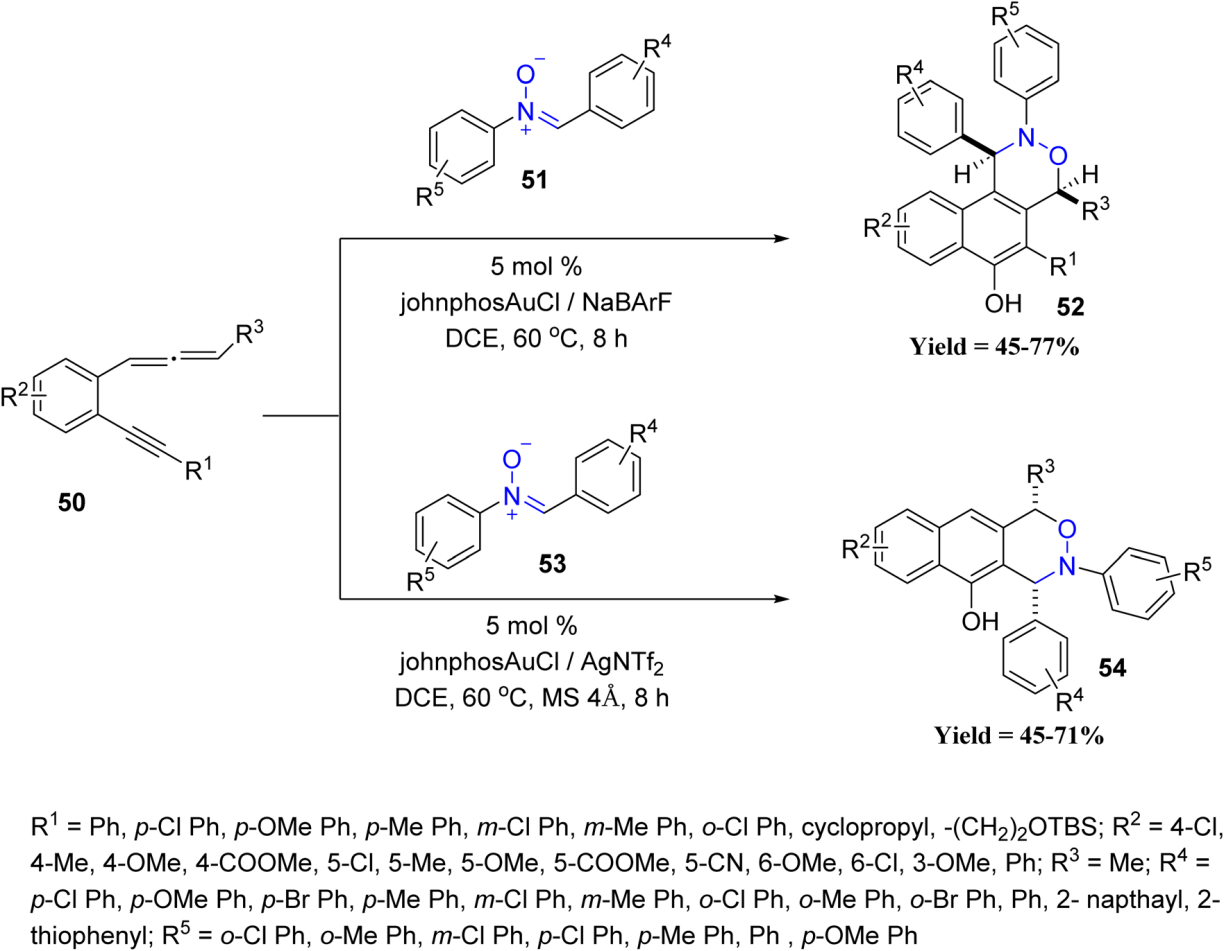
Gold-catalyzed oxidative annulations of 1,5-allenynes with nitrones.

In 2024, Liu's group showcased the regioselectivity control achievable in Au catalysis through two oxidative annulation pathways of 1,5-allenynes 50 with nitrones 51/53 (Scheme 9). Internal alkynes afforded 3,4-fused nitroxy naphthols 52, while terminal alkynes gave 2,3-fused analogues 54. The study revealed that the nature of the alkyne substituent dictates the annulation outcome, providing a predictable regioselective switch. Despite these productive trends, the methodology exhibited limitations, as substrates outside the 1,5-allenyne framework, non-aromatic nitrone variants, and sterically congested partners failed or gave diminished yields, and regioisomer separation became challenging in several cases.^51^

**Scheme 10:**
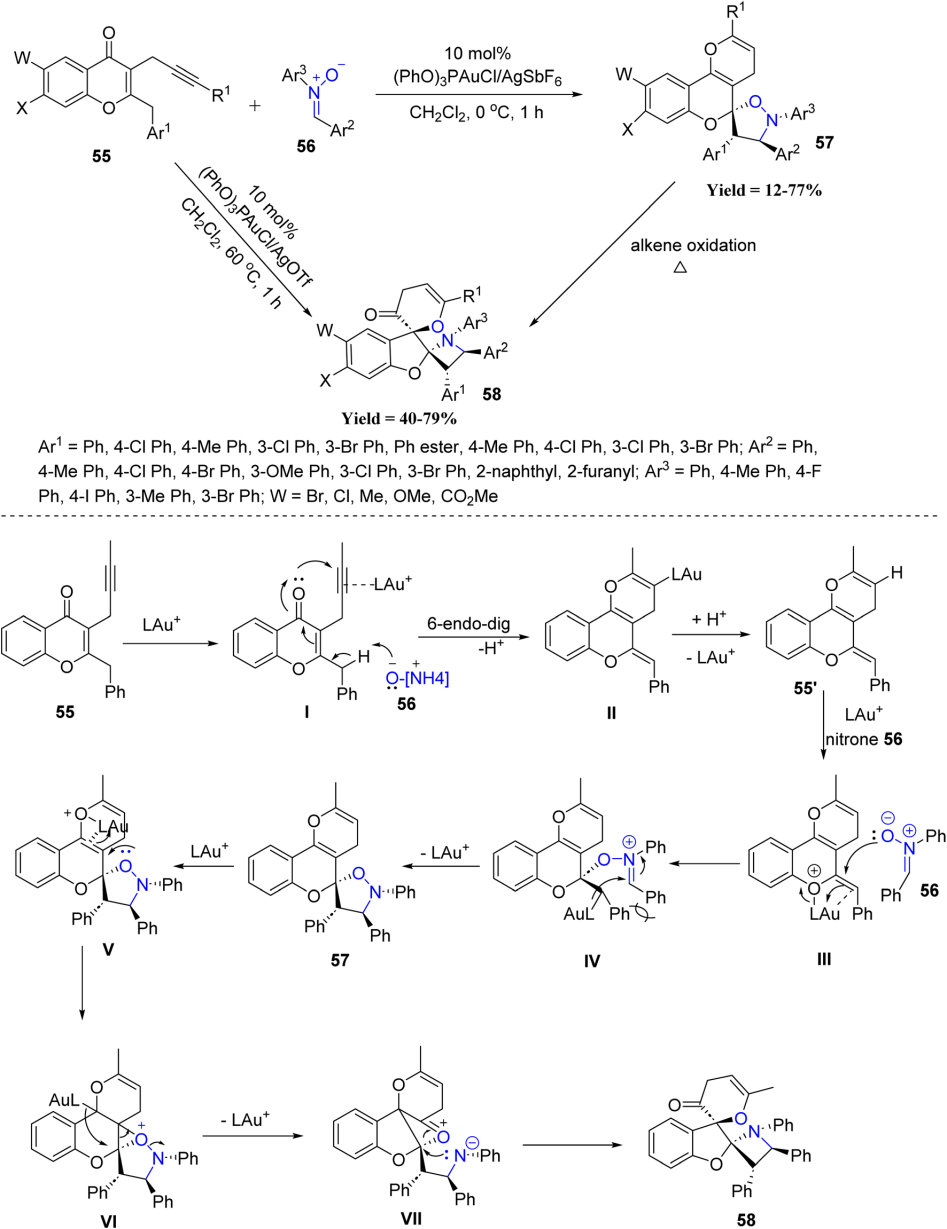
Gold-catalyzed cascade reactions of 2-benzyl-3-alkynyl chromone and nitrone.

Wen-Tai Li and co-workers in 2024 introduced a stereoselective spiro-cyclization protocol employing 2-benzyl-3-alkynylchromones 55 and nitrones 56 (Scheme 10). Mechanistically, gold activation of the alkyne forms intermediates I, which after deprotonation and 6-*endo*-dig cyclization gives alkenyl–gold species II. Subsequent [3 + 2] cycloaddition with the nitrone generates intermediate III (isoxazolidine), which undergoes gold-assisted oxirane formation VI, followed by ring contraction and opening to produce species VII. An intramolecular *N*-attack then completes the formation of the dispiro-benzofuran product 58. This gold-catalyzed stereoselective spiro-cyclization demonstrated a clear reactivity trend, where variations at the C-6 and C-7 positions of the chromone core were well tolerated, and both electron-donating and electron-withdrawing substituents afforded good yields of spiro-isoxazolidine and dispiro-benzofuran products. *Para*- and *meta*-substituted benzylic aryl groups reacted efficiently, while *ortho*-substituted and sterically encumbered partners delivered comparatively lower conversions. However, the method showed limited applicability, as only suitably substituted 2-benzyl-3-alkynylchromones and aryl nitrones were reactive, while sterically hindered, non-aryl, or structurally deviating substrates failed or gave poor yields (Scheme 10).^52^

#### 1.1.3 Ir-Catalyzed

**Scheme 11:**
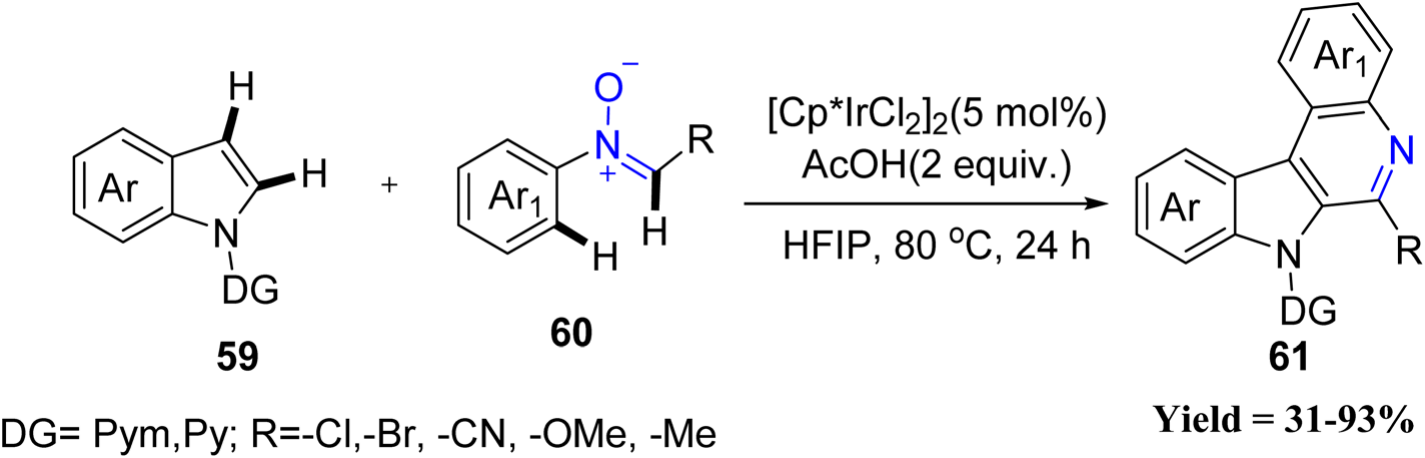
Iridium-catalyzed C–H functionalization of indoles with nitrones.

In this context, Song Sun and co-workers in 2021 developed a redox-neutral iridium-catalyzed dual C–H activation at the C2 and C3 positions of indoles 59 with nitrones 60 (Scheme 11). Under the optimized conditions, a wide range of indoles bearing electron-donating, electron-withdrawing, halogen, and heteroaryl substituents smoothly delivered the desired products in good to excellent yields. Likewise, diverse nitrones, including sterically hindered, heteroaryl, naphthyl, cinnamyl, and functionally enriched variants, were well tolerated, affording the fused heterocycles efficiently. Mechanistic investigations revealed that nitrones act not only as coupling partners but also as internal oxidants. However, the method displays a key limitation: it strictly requires the 2-pyridyl directing group, as alternative directing groups or imine analogues failed to produce any product.^53^

#### 1.1.4 Ag-Catalyzed

**Scheme 12:**
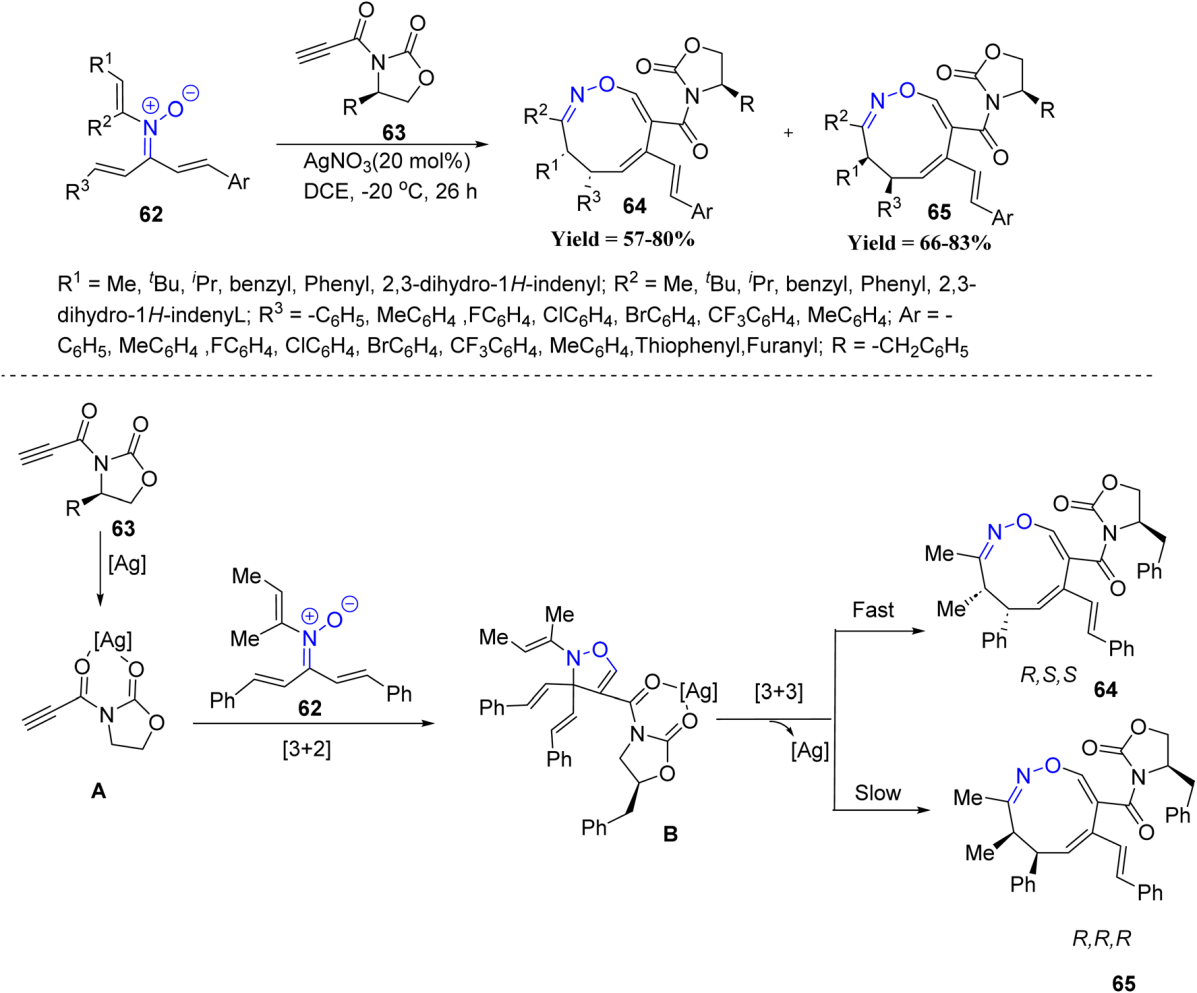
Silver(i)-promoted cycloaddition and rearrangement from *N*-vinyl-α,β-unsaturated nitrones with chiral 3-propioloyloxazolidin-2-ones.

A key contribution to silver catalysis was reported by Dong-Liang Mo and co-workers in 2021, who designed a [3 + 2] cycloaddition/[3,3]-sigmatropic rearrangement cascade between *N*-vinyl α,β-unsaturated nitrones 62 and chiral propioloyloxazolidin-2-ones 63 (Scheme 12). This strategy provided access to chiral nine-membered N-heterocycles 64/65 with excellent diastereoselectivity. Systematic evaluation revealed that oxazolidinones bearing Me, ^i^Pr, ^*t*^Bu, Ph, or indenyl substituents afforded the corresponding cycloadducts in good yields and tunable diastereoselectivity, with bulkier substituents enhancing stereocontrol. The reaction exhibited broad nitrone compatibility: aryl, heteroaryl, alkyl-, and ring-substituted *N*-vinyl nitrones efficiently furnished the nine-membered heterocycles in moderate to good yields as single diastereomers under low-temperature conditions. Notably, attempts to perform the reaction at room temperature led to inseparable mixtures of the two diastereomers, highlighting a key limitation, as strict temperature control is essential to obtain single isomers. Mechanistically, coordination of the oxazolidinone substrate to the silver catalyst generates intermediate A, which undergoes [3 + 2] cycloaddition with the nitrone to give intermediate B. A subsequent [3,3]-sigmatropic rearrangement furnishes the observed products 64 and 65, with the silver catalyst being regenerated through a reversible equilibrium. This work highlights the ability of silver catalysis to combine pericyclic processes in a highly stereoselective fashion, offering a valuable entry to architecturally complex N-heterocycles (Scheme 12).^54^

#### 1.1.5 Rh-Catalyzed

**Scheme 13:**
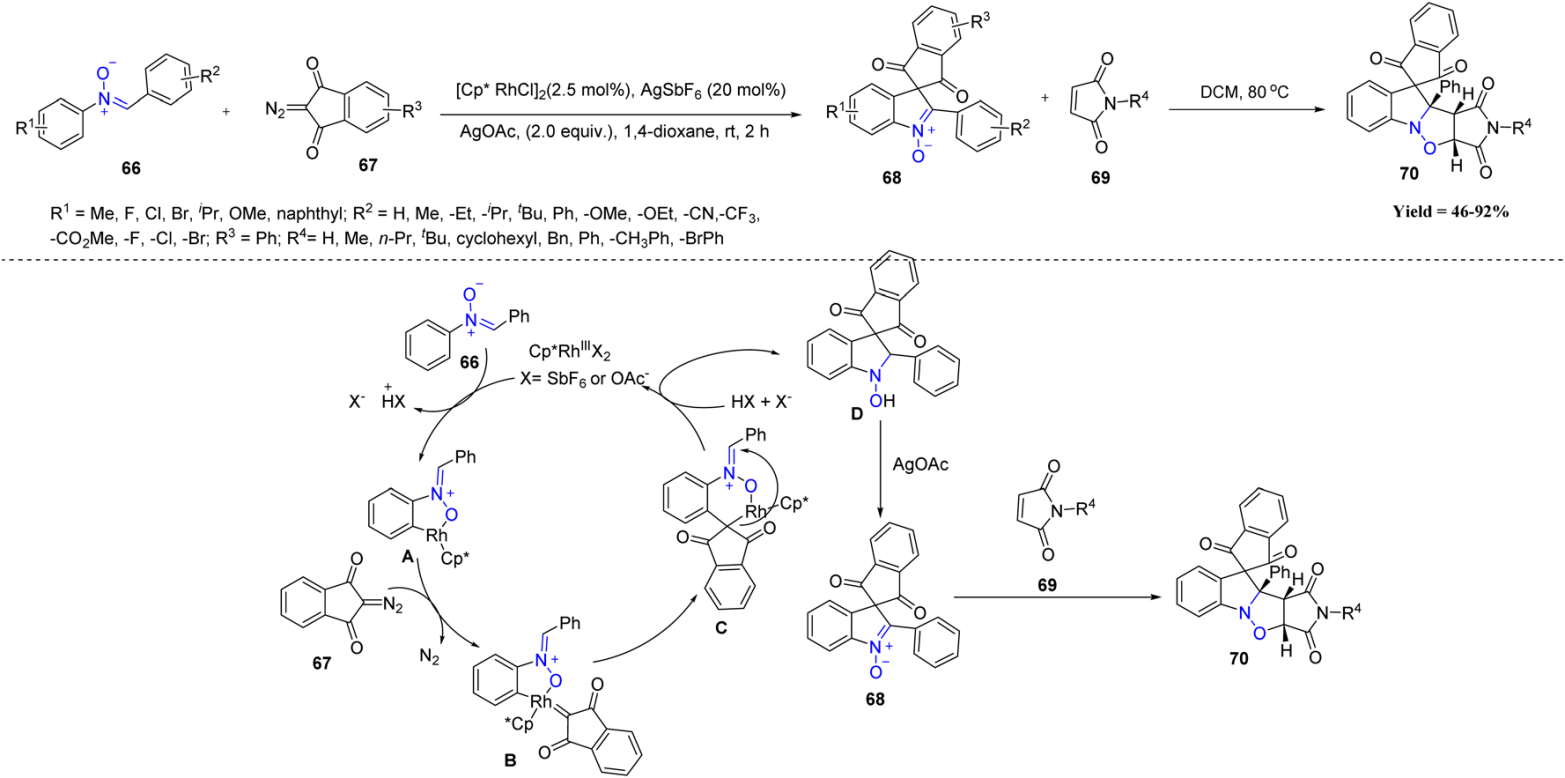
Rh(iii)-Catalyzed spiroannulation reaction of *N*-aryl nitrones with 2-diazo-1,3-indandiones.

In recent years, Xuesen Fan *et al.* demonstrated an efficient and mild Rh(iii)-catalyzed [4 + 1] spiro-annulation strategy for the synthesis of spirocyclic indole-*N*-oxide derivatives 68. The reaction involves *N*-aryl nitrones 66 and 2-diazo-1,3-indandiones 67 as C1 synthons, proceeding *via* a C–H activation/spiro-annulation cascade under extremely mild conditions. The method showed excellent functional-group tolerance: *para*-, *meta*-, and even several *ortho*-substituted nitrones bearing alkyl, alkoxy, halogen, CF_3_, CN, CO_2_Me, heteroaryl, alkenyl, naphthyl, and disubstituted aryl groups reacted smoothly, delivering the products in moderate to excellent yields. The diazo component also displayed wide scope, accommodating alkyl-, halo-, dimethyl-substituted, and naphthalene-fused variants, as well as six-membered diazo compounds. A notable limitation, however, is that certain *ortho*-substituted nitrones particularly *ortho*-Me failed to undergo spirocyclization due to steric hindrance. Initially, the reaction begins with C–H activation of the *N*-aryl nitrone 66 by the Rh(iii) catalyst to form a rhoda-cycle intermediate A. Coordination with the diazo compound 67 generates a Rh-carbene B, which undergoes migratory insertion followed by intramolecular nucleophilic attack on the C=N bond. Protonation regenerates the catalyst and forms a key intermediate D, which is subsequently oxidized by AgOAc to deliver the spirocyclic indole-*N*-oxide 68. The product 68 is then successfully employed in 1,3-dipolar cycloaddition reactions with maleimides 69, leading to the formation of structurally complex maleimide-fused polycyclic scaffolds 70 (Scheme 13).^55^

**Scheme 14:**
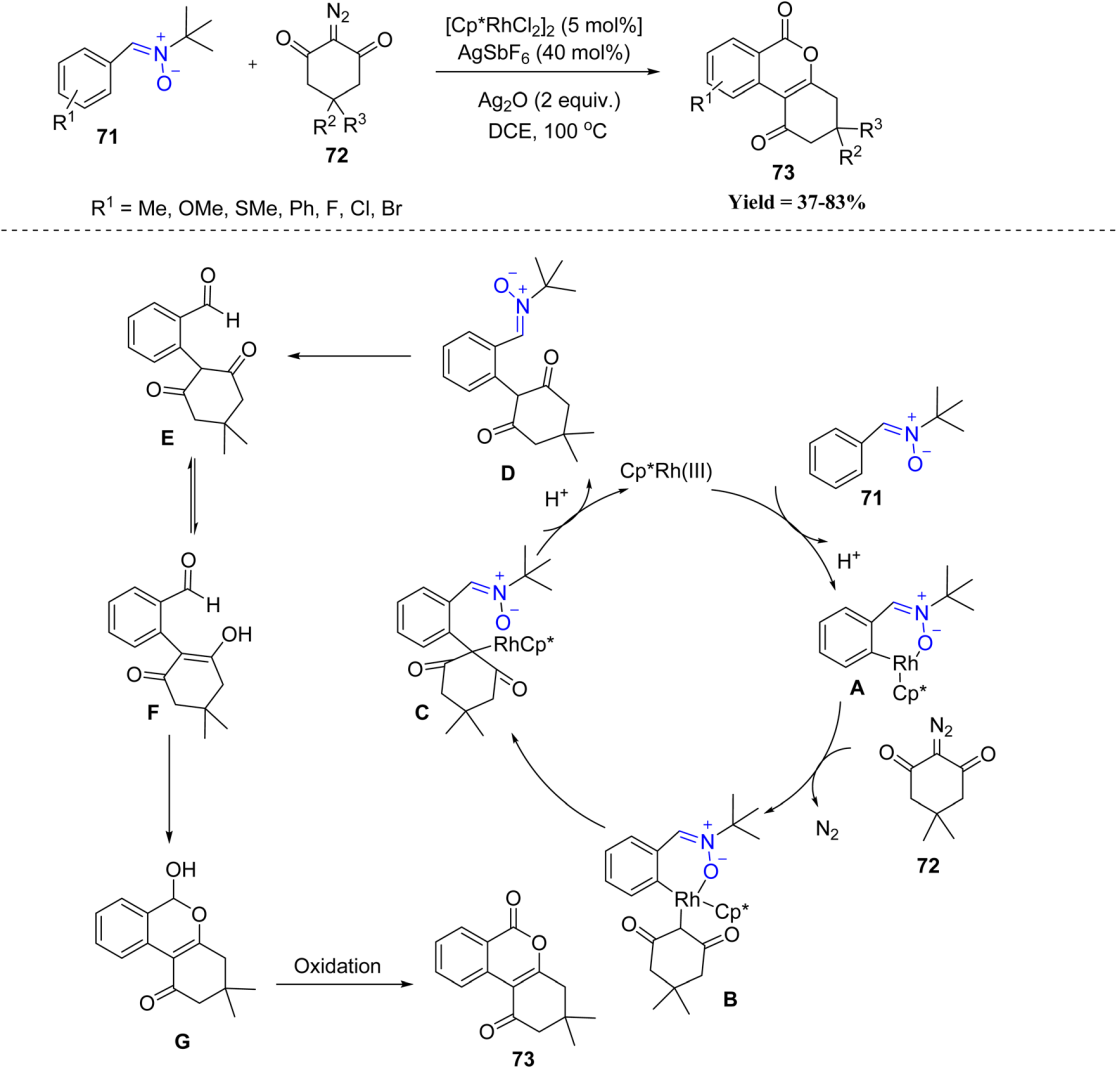
Rh(iii) catalyzed C–H annulation of nitrones with cyclic 2-diazo-1,3 diones.

Following this, Li-Ming Zhao *et al.* in 2023, developed a Rh(iii)-catalyzed cascade reaction of nitrones 71 and cyclic 2-diazo-1,3-diones 72 for the efficient synthesis of angular chromendiones. The nitrone serves as a traceless directing group, enabling C–H activation and annulation. Electron-donating nitrones showed the highest reactivity, while electron-withdrawing or strongly electron-deficient nitrones failed. Substitution pattern had minimal effect, though *ortho*-substituted nitrones gave lower yields due to steric hindrance. A range of cyclic diazo compounds was tolerated, but unsubstituted or indantrione-based diazos reacted poorly. Overall, the method is efficient but limited by electron-poor nitrones and specific less-reactive diazo partners. Mechanistically, the reaction starts with coordination of a cationic Rh(iii) catalyst to nitrone, followed by C–H activation to form a six-membered rhodacycle A. Then A reacts with a diazo compound, releasing N_2_ to generate a Rh-carbene intermediate B. Migratory insertion of the Rh–aryl bond forms a seven-membered rhodacycle C, which undergoes protonolysis and ligand dissociation to produce intermediate D, regenerating the Rh(iii) catalyst. The nitrone in D rapidly decomposes into an aldehyde, yielding intermediate E, an aryl-substituted 1,3-diketone. Tautomerization to the enol form F is followed by intramolecular nucleophilic attack on the carbonyl, forming cyclic hemiacetal G. Final oxidation of G produced the angular chromendione 73 (Scheme 14).^56^

**Scheme 15:**
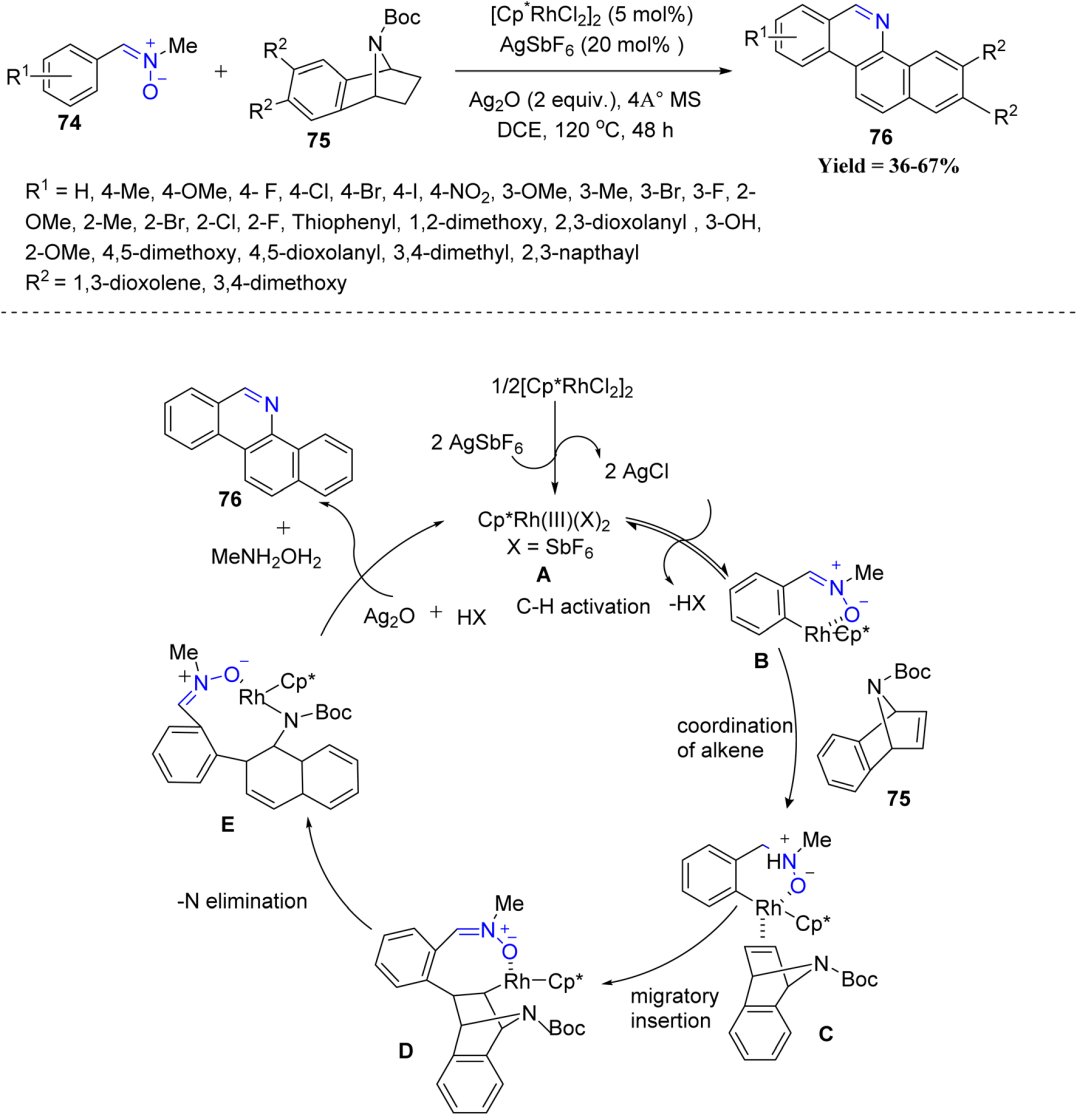
Rh(iii) catalyzed 7-azabenzonorbornadienes and aryl nitrones.

Masilamani Jeganmohan *et al.* applied Rh(iii) catalysis to a one-pot synthesis of benzo[*c*]phenanthridine alkaloids 76 through a cascade involving C–C bond formation and cycloaromatization. In this method, aryl nitrones 74 react with 7-azabenzonorbornadienes 75 to afford various biologically important benzo[*c*]phenanthridine derivatives 76 in moderate to good yields. A wide range of nitrones including electron-neutral, electron-rich, halogenated, unsymmetrical, disubstituted, dimethyl, and naphthyl variants reacted efficiently, with electron-donating nitrones showing superior reactivity. The method also tolerated diverse azabenzonorbornadiene partners and enabled the rapid synthesis of bioactive alkaloids such as norchelerythrine, norsanguinarine, and decarine. However, the reaction failed with 2-thiophenyl nitrones due to catalyst chelation, and electron-deficient nitrones delivered comparatively lower yields, marking the main limitations of the protocol. Mechanistically, the active cationic Rh(iii) species A is generated *via* ligand exchange between a rhodium dimer and AgSbF_6_. The aryl nitrone coordinates to this Rh(iii) center through its oxygen atom, forming a six-membered rhodacycle intermediate B*via* a deprotonation process. Subsequent coordination and migratory insertion of the azabenzonorbornadiene lead to an eight-membered rhodacycle D, which undergoes β-nitrogen elimination to yield an intermediate E bearing a reactive imine motif. A final intramolecular cyclization and aromatization step, assisted by silver oxide, completes the transformation and regenerates the rhodium catalyst (Scheme 15).^57^

**Scheme 16:**
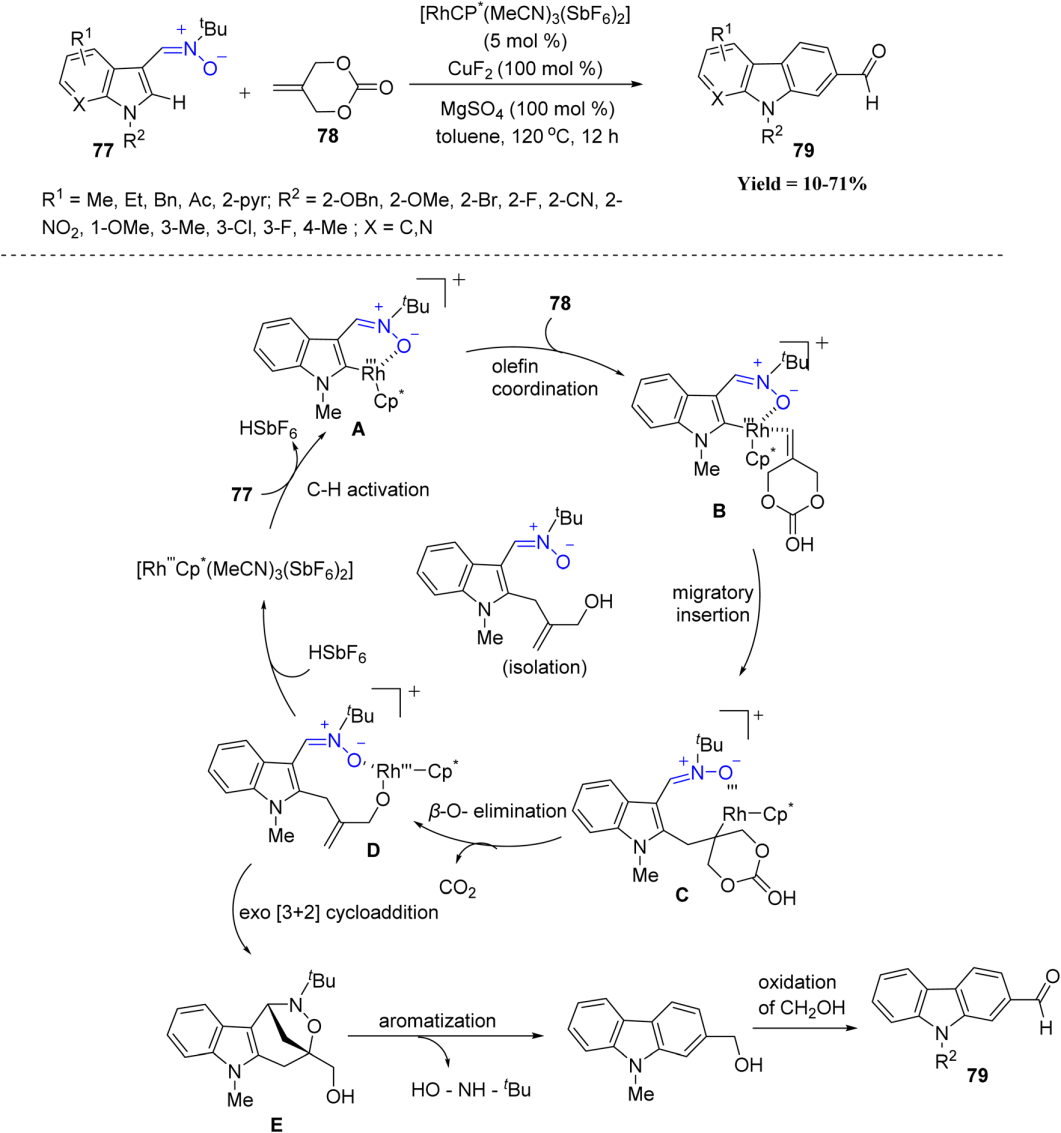
Rhodium(iii)-catalyzed cross-coupling reaction between indolyl nitrones and 2-methylidene cyclic carbonate.

Similarly, Su Kim *et al.* in 2023 disclosed a novel Rh(iii)-catalyzed cross-coupling between indolyl nitrones 77 and 2-methylidene cyclic carbonates 78, enabling the synthesis of C2-formylated carbazoles derivatives 79. Various N-protected and C4–C7 substituted indoles, as well as heteroaryl nitrones, reacted smoothly, delivering the products in good yields. Mechanistically, a cationic rhodium(iii) catalyst promotes selective C2–H activation of the indolyl nitrone to form a rhodacycle intermediate A. Subsequent coordination and migratory insertion of the cyclic carbonate yields an O–Rh–C complex C, which undergoes β-*O*-elimination followed by an *exo*-type [3 + 2] cycloaddition to form a bridged heterocycle E. Aromatization and aerobic oxidation of the resulting benzylic alcohol complete the transformation, furnishing the C2-formylated carbazole scaffold 79. However, the method showed clear limitations: sterically bulkier carbonates (*e.g.*, benzylidene carbonate) completely inhibited the reaction, and altered vinyl carbonate structures diverted the pathway to undesired C3-formylation (Scheme 16).^58^

**Scheme 17:**
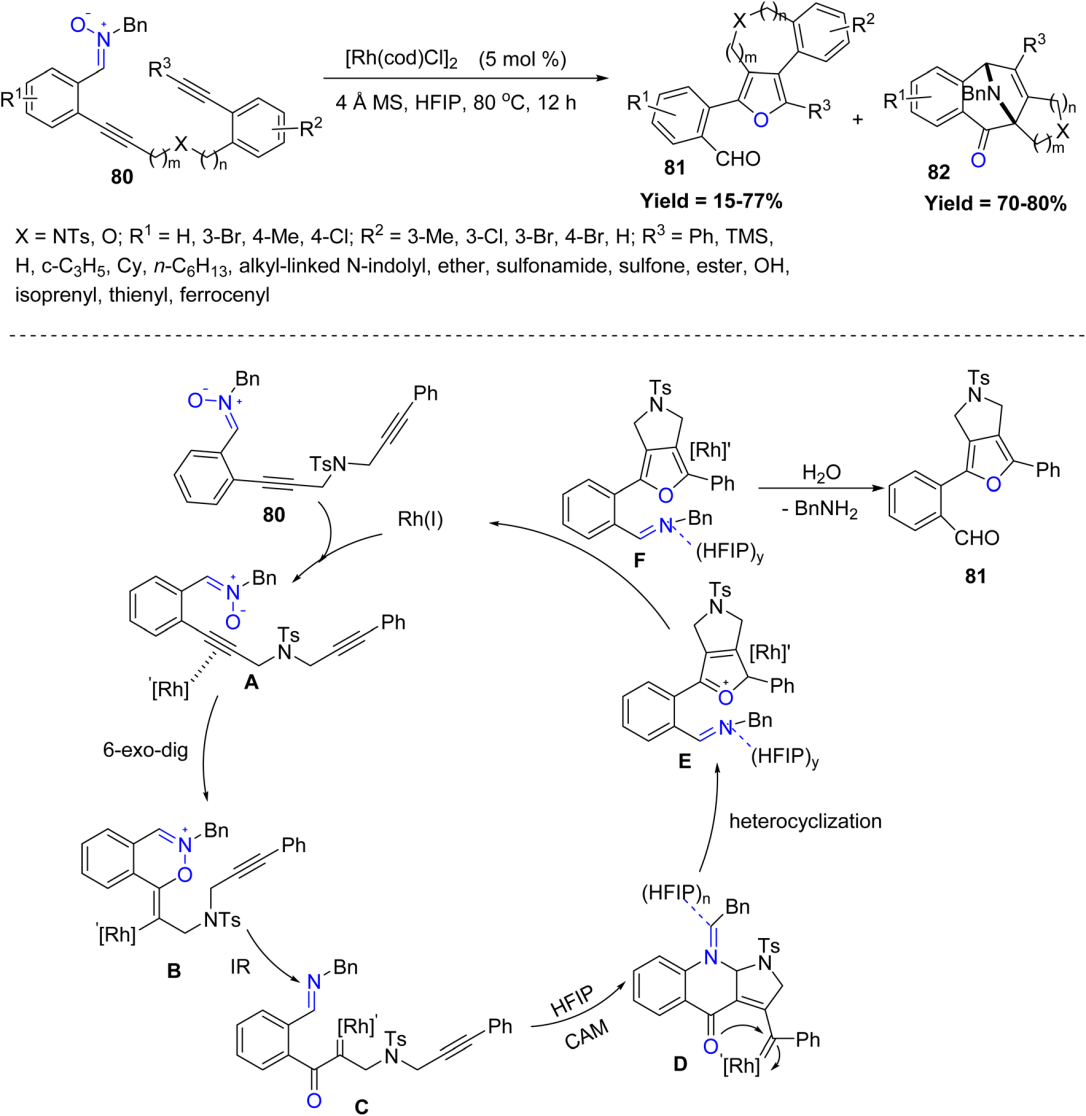
Rhodium(i)-catalyzed cascade annulation of 1,*n*-diynyl nitrones.

Further extending Rh(i) catalysis, Weidong Rao *et al.* reported an efficient rhodium(i)-catalyzed strategy for constructing fully substituted furans featuring a 3,4-fused 5–8-membered carbocycle, heterocycle, or spirocycle in 2024. The transformation involves a cascade annulation of 1,*n*-diynyl nitrones, facilitated by 1,1,1,3,3,3-hexafluoroisopropanol (HFIP) as a critical additive. 1,7- and 1,8-diynyl nitrones showed the highest reactivity, O-tethered variants also worked well, while sterically congested or terminal-alkyne substrates and longer tethers (*e.g.*, 1,9-diynyl) displayed markedly reduced reactivity. Mechanistic investigations using 1,6-diynyl nitrone as a model substrate revealed that the Rh(i) catalyst selectively coordinates with the *ortho*-alkynylphenyl moiety to form a Rh-bound intermediate A. This species undergoes a 6-*exo*-dig oxycyclization, generating a vinyl-rhodium intermediate B. A subsequent N–O bond cleavage driven by an internal redox process leads to the formation of an α-oxo rhodium carbene C. Interestingly, instead of following the conventional azomethine ylide pathway, the imino nitrogen of the intermediate interacts *via* hydrogen bonding with the HFIP solvent, altering the course of reactivity. This unique interaction enables the carbene intermediate to undergo a cascade cyclization *via* a carbene-assisted mechanism (CAM), yielding a γ-oxo rhodium carbene complex D. Further intramolecular nucleophilic attack by the oxygen atom on the carbene center leads to heterocyclization, forming an oxonium intermediate E. Demetalation and subsequent hydrolysis during work-up yield the final 3,4-fused fully substituted furan product 81 along with benzylamine 82 as a byproduct. However, limitations included decomposition of terminal-alkyne substrates and poor yields from 1,6- and especially 1,9-diynyl nitrones due to unfavourable transannular and entropic effects (Scheme 17).^59^

#### 1.1.6 Co-Catalyzed

**Scheme 18:**
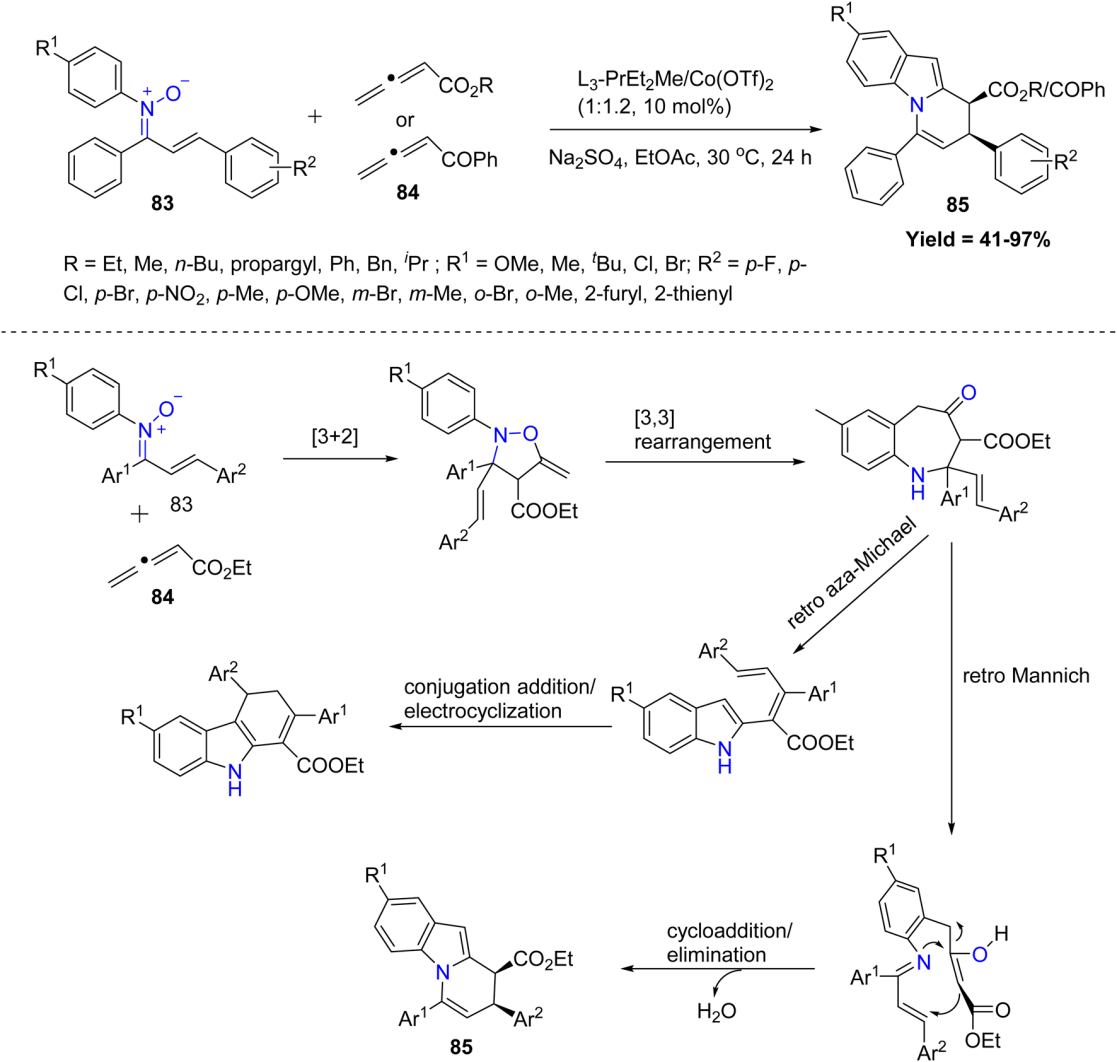
Cobalt(ii) complex catalytic multistep cascade reaction of α,β-unsaturated-*N*-aryl nitroneswithallenes.

Xiaoming Feng *et al.* in 2022, developed an efficient *N*,*N*-dioxide/Co(ii) catalytic system for the cascade reaction of α,β-unsaturated *N*-aryl nitrones 83 with allenes 84, yielding dihydro pyridoindoles 85 in good yields with excellent diastereo- and enantioselectivity under mild conditions. The reaction proceeds *via* an initial [3 + 2] cycloaddition, followed by a [3,3]-sigmatropic rearrangement to form a benzazepine intermediate, which selectively undergoes a retro-Mannich pathway under cobalt catalysis to give the desired products 85. Diverse allenoate esters (Me, *n*-Bu, propargyl, Ph, Bn) and a wide range of α,β-unsaturated *N*-aryl nitrones including electron-rich, electron-poor, *ortho*/*meta*/*para*-substituted, heteroaryl, and ring-fused systems gave the dihydropyridoindoles in good yields with excellent diastereo- and enantioselectivity. The main limitations were decreased stereocontrol with sterically bulky allene esters and modest yields for some highly substituted or heteroaryl nitrones, indicating steric and electronic sensitivity at the nitrone component (Scheme 18).^60^

**Scheme 19:**
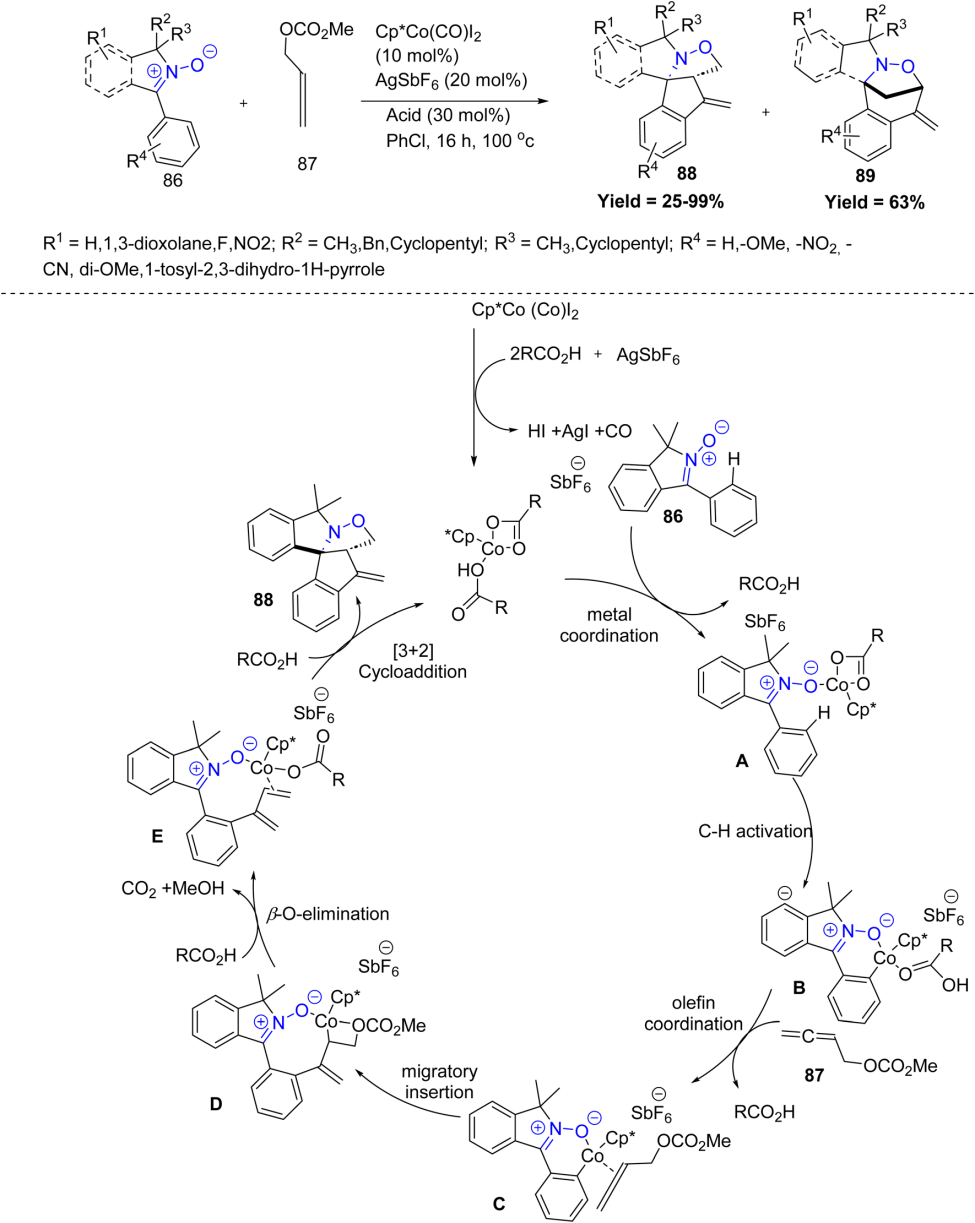
Co(iii) catalyzed dipolar cycloaddition reaction of cyclic *C*-aryl nitrones with 2,3-butadien-1-ol carbonates.

More recently, Rafał Loska *et al.* in 2023, developed a tandem strategy has been developed for the construction of complex spirocyclic isoindolines 88 and pyrrolidines 89, involving Co(iii)-catalyzed dienylation of cyclic *C*-aryl nitrones 86 with 2,3-butadien-1-ol carbonates 87, followed by intramolecular 1,3-dipolar cycloaddition. In this process, the nitrone moiety functions dually as the directing group for C(aryl)–H activation and as the dipole in the cycloaddition step. Notably, the regioselectivity between fused 88 and bridged products 89 can be tuned by varying the reaction temperature. The proposed catalytic cycle begins with nitrone-directed C–H activation by a carboxylate–Co(iii) complex, followed by nucleophilic attack on the central carbon of cumulene 87. Subsequent elimination furnishes a diene intermediate E, which undergoes a rapid intramolecular 1,3-dipolar cycloaddition to form spirocyclic isoindolines 88. Electron-rich nitrones generally provided better regioselectivity, and both EDG- and EWG-substituted substrates were well tolerated. Moderately substituted allenes reacted efficiently, while bulky *N*-substituents enhanced diastereoselectivity. Key limitations include the failure of heteroaryl nitrones, sterically congested aryl groups (*e.g.*, 2,4-dimethoxyphenyl) that impede C–H activation, reduced yields with cyano-containing nitrones, and poor reactivity of heavily substituted allenes or sterically hindered diene intermediates (Scheme 19).^61^

#### 1.1.7 Yb-Catalyzed

**Scheme 20:**
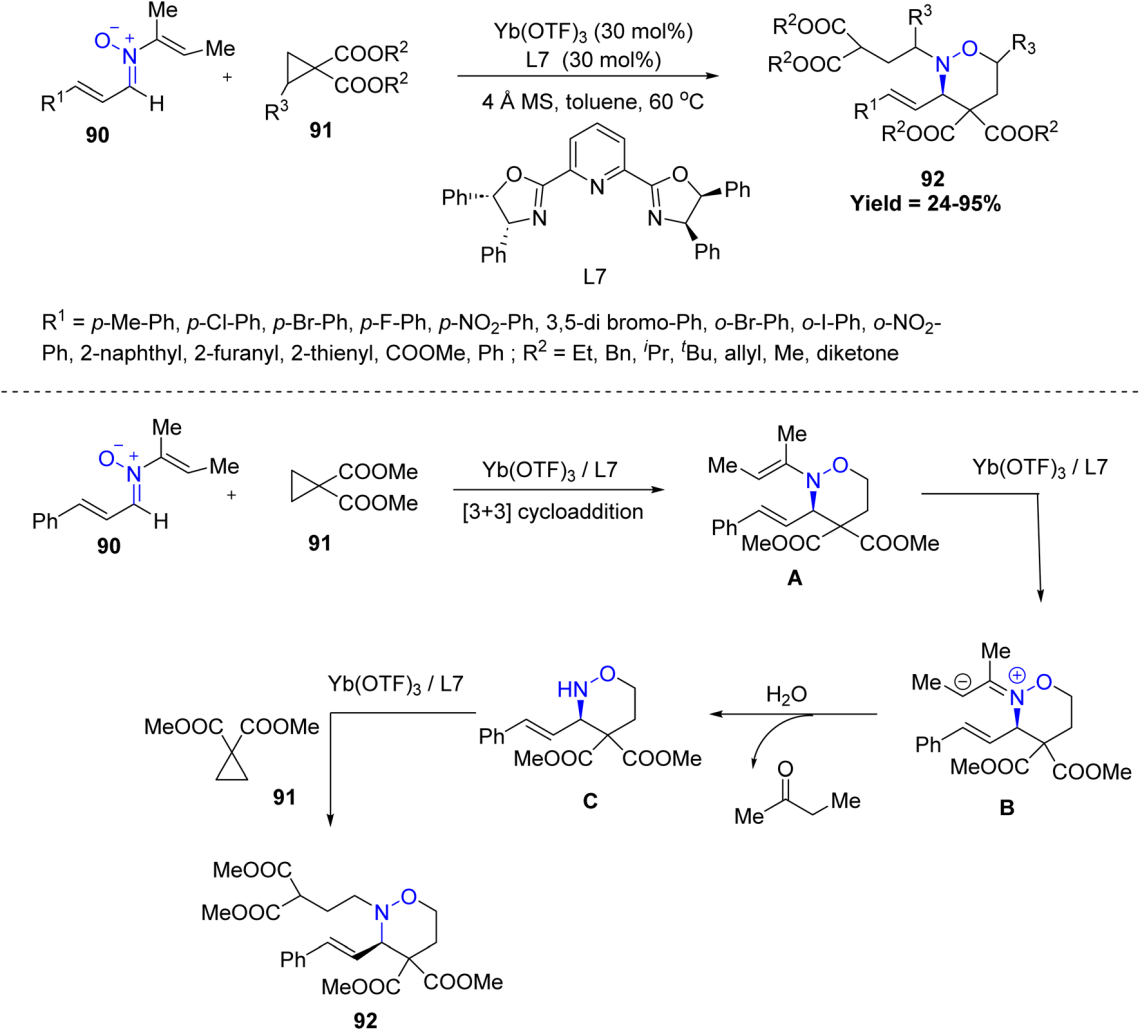
Yb(Otf)_3_-Catalyzed asymmetric [3 + 3] cycloaddition of *N*-vinyl nitrones with activated cyclopropanes.

Dong-Liang Mo *et al.* in 2025, developed a three-component asymmetric [3 + 3] cycloaddition reaction between *N*-vinyl cinnamaldehyde nitrones 90 and activated cyclopropanes 91 for the efficient synthesis of diverse functionalized 1,2-oxazines 92 in presence of Yb(OTf)_3_, as catalyst. This reaction offers wide substrate scope, good functional group tolerance, and efficient asymmetric [3 + 3] cycloaddition in a domino sequence. *Para*-substituted aryl nitrones showed the highest reactivity, whereas *ortho*-substituted or aliphatic ester nitrones were significantly less effective. Simple ester-substituted cyclopropanes performed well, but bulky ^*t*^Bu or aromatic groups consistently lowered yields and selectivity. Mechanistically, the Yb(OTf)_3_/L7 catalytic system promotes the ring-opening of cyclopropane 91, which then undergoes [3 + 3] cycloaddition with nitrone 90 to form intermediate A. This species quickly isomerizes to a zwitterionic intermediate B under the influence of the strong Lewis acid catalyst. Hydrolysis of B yields 2-butanone as a byproduct and produces intermediate C. Finally, C undergoes nucleophilic attack on another molecule of cyclopropane 91 in the presence of the Lewis acid to afford the final product 92. Thus, despite broad applicability, the reaction is limited by steric congestion, strong EWGs, and heavily substituted cyclopropanes (Scheme 20).^62^

#### 1.1.8 Bi-Catalyzed

**Scheme 21:**
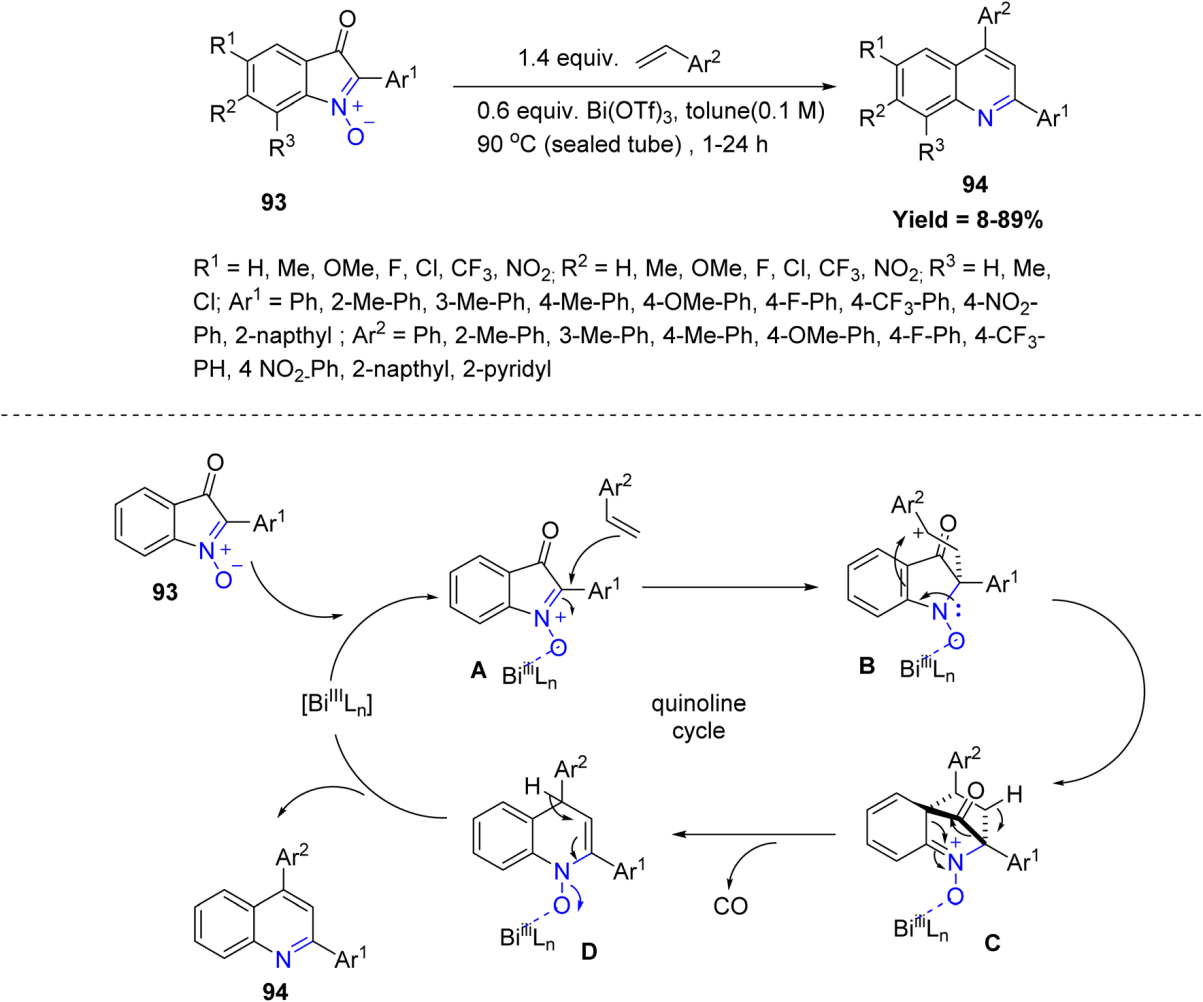
Bi(OTf)_3_-Catalyzed cascade cycloaddition–decarbonylation–*N*-deoxygenative aromatization between 2-arylisatogens and styrenes.

A significant contribution in this field was made by Charnsak Thongsornkleeb and co-workers in 2024, who reported a novel Bi(OTf)_3_-catalyzed transformation of 2-arylisatogens 93 with vinyl arenes to synthesize 2,4-diarylquinolines 94. This unprecedented reaction proceeds efficiently across a broad substrate scope, affording the desired quinoline derivatives 94 in excellent yields. Mechanistically, the Bi(iii) catalyst first coordinates with the nitrone oxygen of the isatogen to generate an electrophilic complex A. Subsequent nucleophilic attack by the vinyl arene occurs in a stepwise manner, forming a benzylic cationic intermediate B that is stabilized by resonance with the adjacent aryl ring. This leads to the formation of a strained tricyclic species C, which undergoes rapid carbon monoxide extrusion to generate an intermediate D that aromatizes to yield the final quinoline product 94, completing the catalytic cycle. The reaction showed good tolerance for electronically neutral substrates, while strong electron-donating or withdrawing substituents and steric hindrance markedly reduced yields or diverted the reaction to anthranils. Styrenes bearing *para*-substituents reacted well, whereas *ortho*-substituted and heteroaryl alkenes were ineffective. Non-styrenyl alkenes generally failed, highlighting limitations related to electronic effects, steric congestion, and catalyst deactivation (Scheme 21).^63^

#### 1.1.9 Ru-Catalyzed

**Scheme 22:**
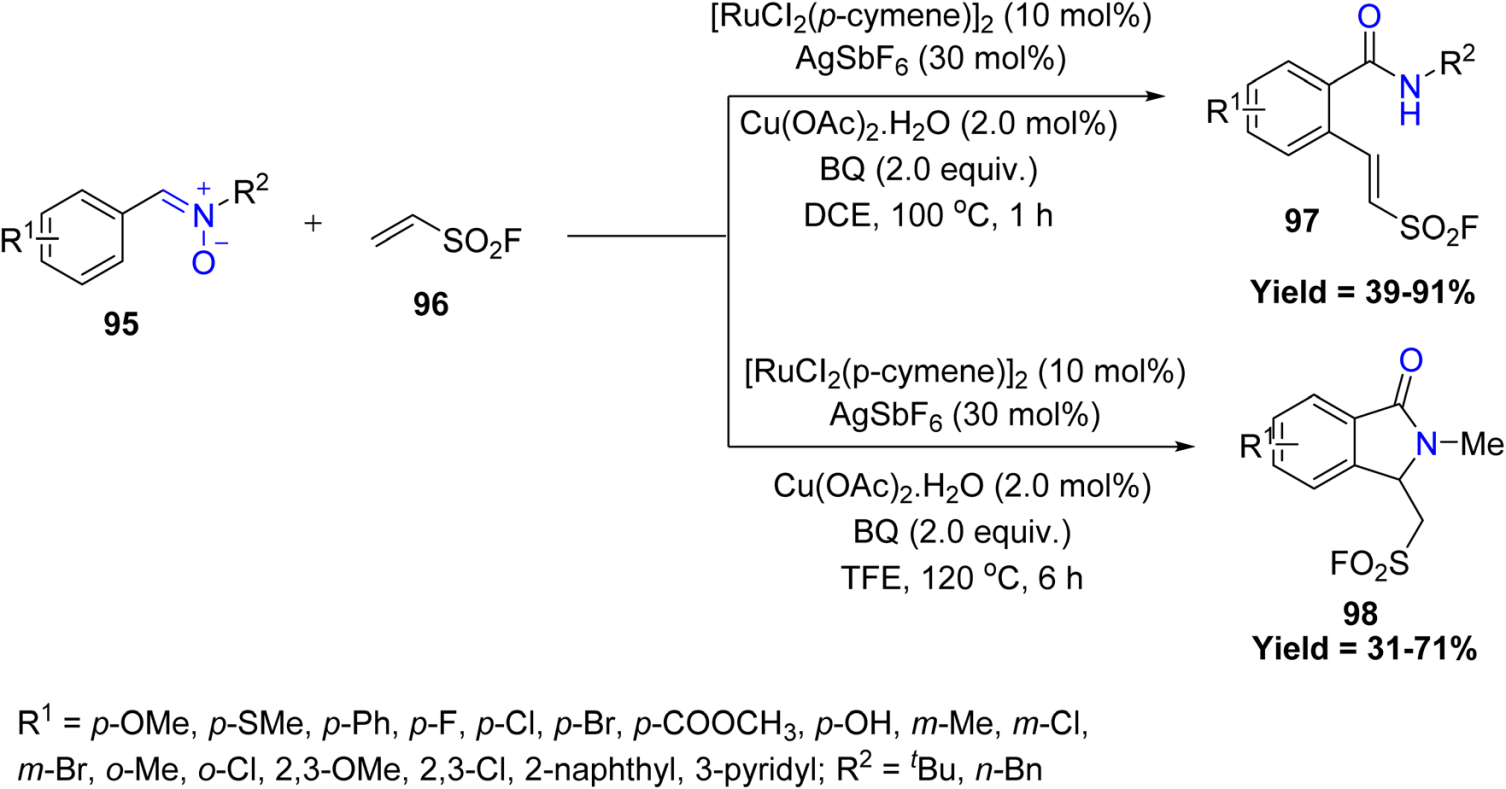
Ru-Catalyzed C–H activation of nitrones with ethenesulfonyl fluoride.

In this context, in 2022, Li-Ming Zhao *et al.* demonstrated, a Ru-catalyzed C–H activation strategy allowing for the effective alkenylation of nitrones 95 to give 2-arylethenesulfonyl fluorides 97 and isoindolinones 98 in good yields. Here, directing group concomitantly converts to useful amide moieties (Scheme 22). Electron-rich and moderately substituted aryl nitrones show the highest reactivity, while steric congestion and heteroaryl or strongly withdrawing groups significantly reduce efficiency. *N*-Substituent size strongly influences the pathway, with small groups causing side reactions and limiting overall substrate scope.^64^

#### 1.1.10 Pd-Catalyzed

**Scheme 23:**
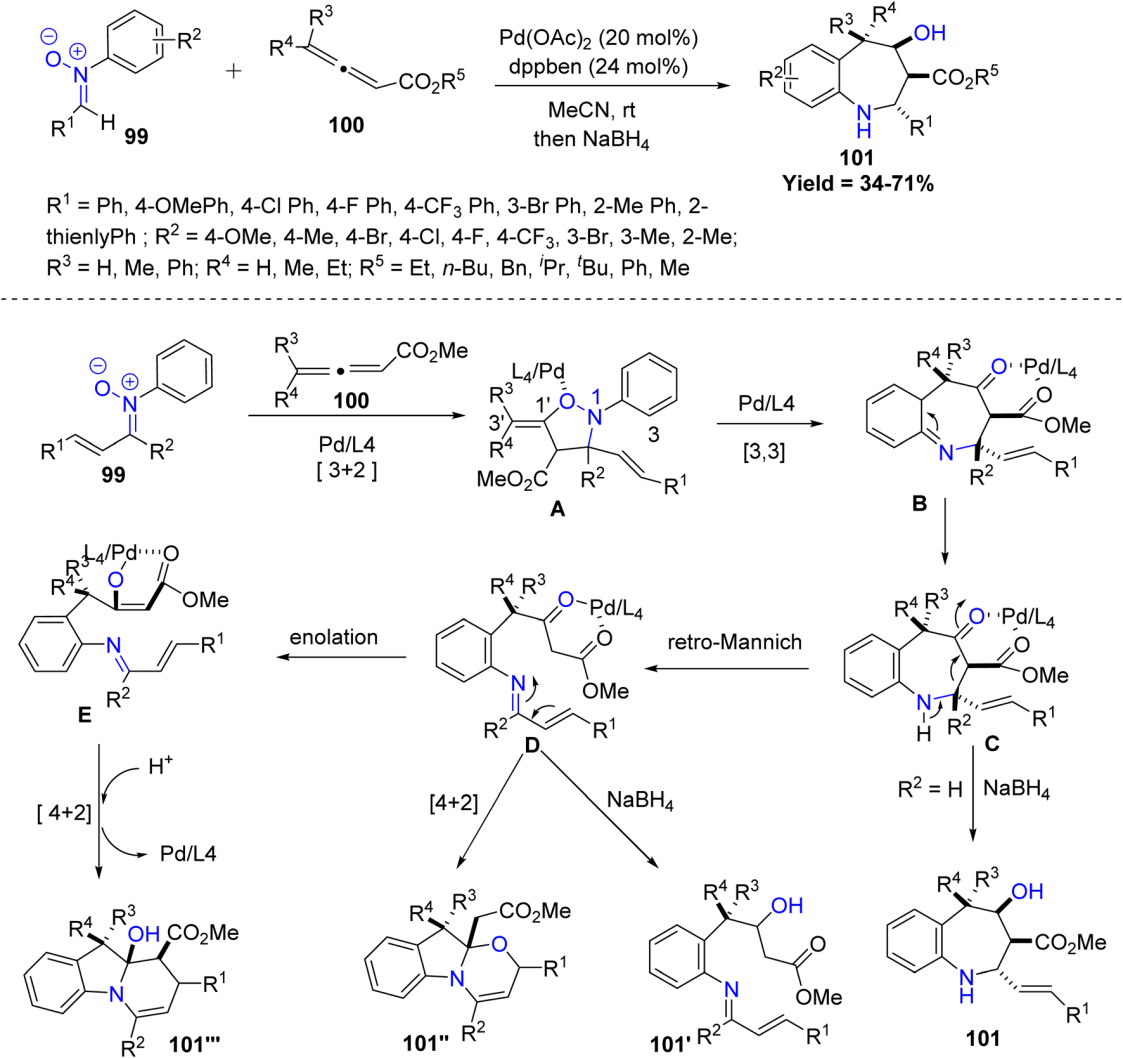
Pd(ii)-Catalyzed [3 + 2] cycloaddition of *N*-aryl nitrones with allenoates.

A recent study by Dong-Liang Mo and co-workers showcased this potential through a Pd(ii)-catalyzed diastereoselective synthesis of benzazepines featuring three contiguous carbon stereocenters. The transformation begins with a [3 + 2] cycloaddition between *N*-aryl nitrones 99 and allenoates 100, generating an isooxazoline intermediate A that undergoes N–O bond cleavage and a [3,3]-sigmatropic rearrangement, followed by aromatization to deliver a benzazepinone scaffold. Further treatment with NaBH_4_ delivers the reduced benzazepine product 101. Alternatively, the benzazepinone can undergo a *retro*-Mannich reaction to generate an imine intermediate, which either proceeds through an intramolecular *O*-[4 + 2] cycloaddition to form a bridged heterocycle 101″ or is reduced to another product 101′ while regenerating the active Pd catalyst. In another pathway, isomerization of the imine yields an enolate–azadiene intermediate, which undergoes an intramolecular [4 + 2] cycloaddition to furnish a distinct polycyclic compound 101‴, again completing the catalytic cycle. The reaction works best with electron-rich or moderately substituted *N*-aryl nitrones and various allenoates, giving good yields. However, sterically hindered nitrones, heteroaryl nitrones, and bulky or ketone-derived allenes give lower yields, limiting the substrate scope (Scheme 23).^65^

#### 1.1.11 Fe-Catalyzed

**Scheme 24:**
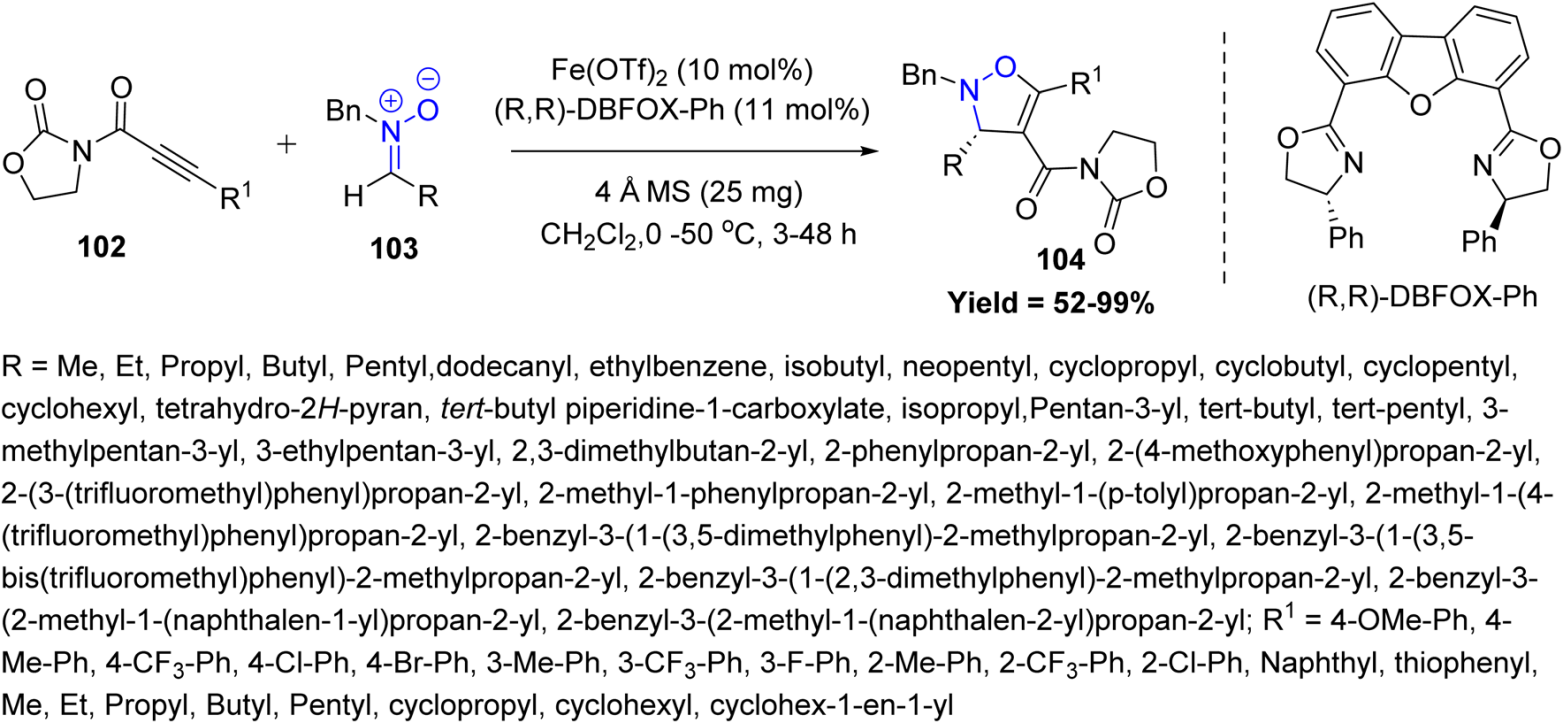
Iron catalyzed asymmetric clicking of alkynyl dipolarophiles and nitrones.

In 2024, Yi-Yong Huang *et al.*, reported a Fe-catalyzed asymmetric [3 + 2] cycloaddition between nonterminal alkynyl imides 102 and α-aliphatic nitrones 103, efficiently generating chiral 4-isoxazolines 104 bearing 3-alkyl-substituted stereogenic centers. A key advantage of this protocol is its scalability to gram-scale synthesis and its compatibility with facile downstream derivatization, highlighting the synthetic utility of the chiral 4-isoxazoline scaffold and the embedded imide functionality. However, the protocol is limited by sterically bulky nitrones, *ortho*-substituted alkynes, and sensitive or strained alkynes, which exhibit reduced yields or undergo decomposition under the reaction conditions (Scheme 24).^66^

## 2 Metal-free synthesis

Metal-free synthesis has gained significant importance over transition-metal-catalyzed strategies due to its sustainability, cost-effectiveness, and operational simplicity. Unlike transition-metal catalysts, which are often expensive, toxic, and sensitive to air or moisture, metal-free approaches typically rely on cycloaddition reaction, organ catalysts, Lewis's acids, or photochemical activation, making them more environmentally benign and broadly accessible. At the same time, metal-free methods often provide excellent regio- and stereo control, enabling efficient construction of heterocycles from nitrones while maintaining high atom economy and functional group tolerance.^67–71^

### 2.1 Cycloaddition-based

#### 2.1.1 [3 + 2] dipolar cycloaddition

**Scheme 25:**
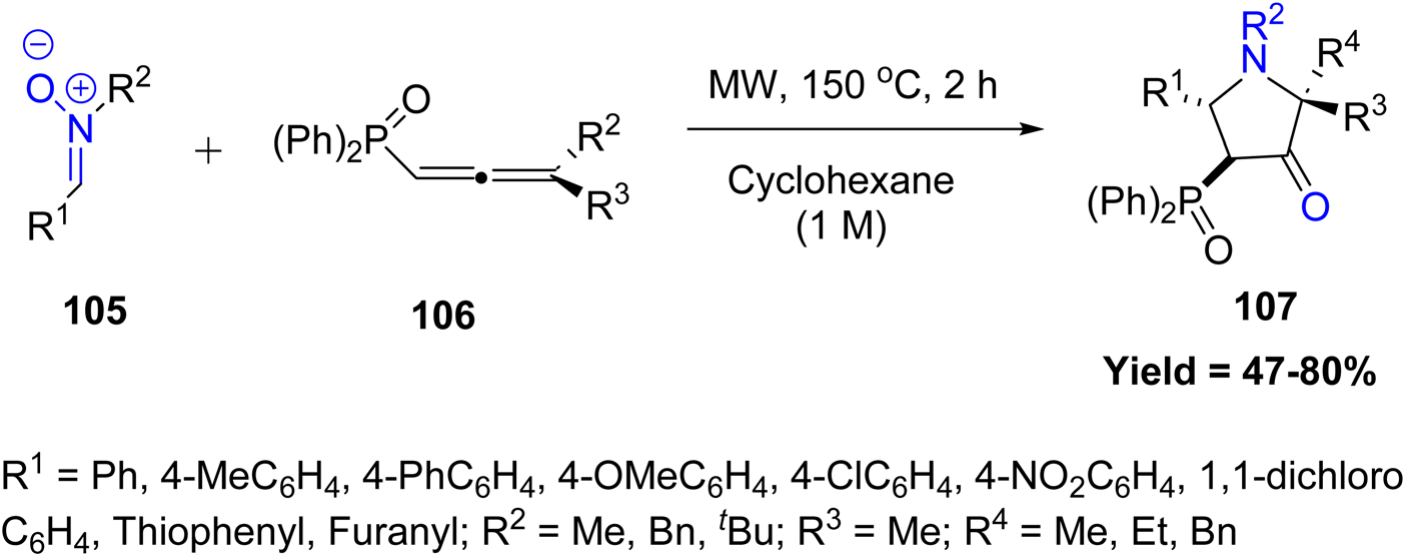
[3 + 2] cycloaddition of nitrones with phosphinylallenes.

The scope of [3 + 2] cycloadditions has been continuously broadened with new substrate classes. For instance, in 2023, Jean-François Poisson and co-workers reported a pioneering 1,3-dipolar cycloaddition between *N*-alkyl nitrones 105 and phosphine oxide-substituted allenes 106, offering a novel and efficient strategy for the synthesis of 4-phosphinylpyrrolidin-3-ones 107. The reaction proceeds with good yields and excellent 4,5-*trans* diastereoselectivity. Remarkably, under the reaction conditions, the initially formed cycloadducts undergo an *in situ* rearrangement to selectively generate the corresponding pyrrolidin-3-one derivatives. Sterically bulky nitrones, highly substituted allenes, and unstable or sensitive substrates show lower reactivity, decomposition, or reduced selectivity, limiting the overall substrate scope (Scheme 25).^72^

**Scheme 26:**
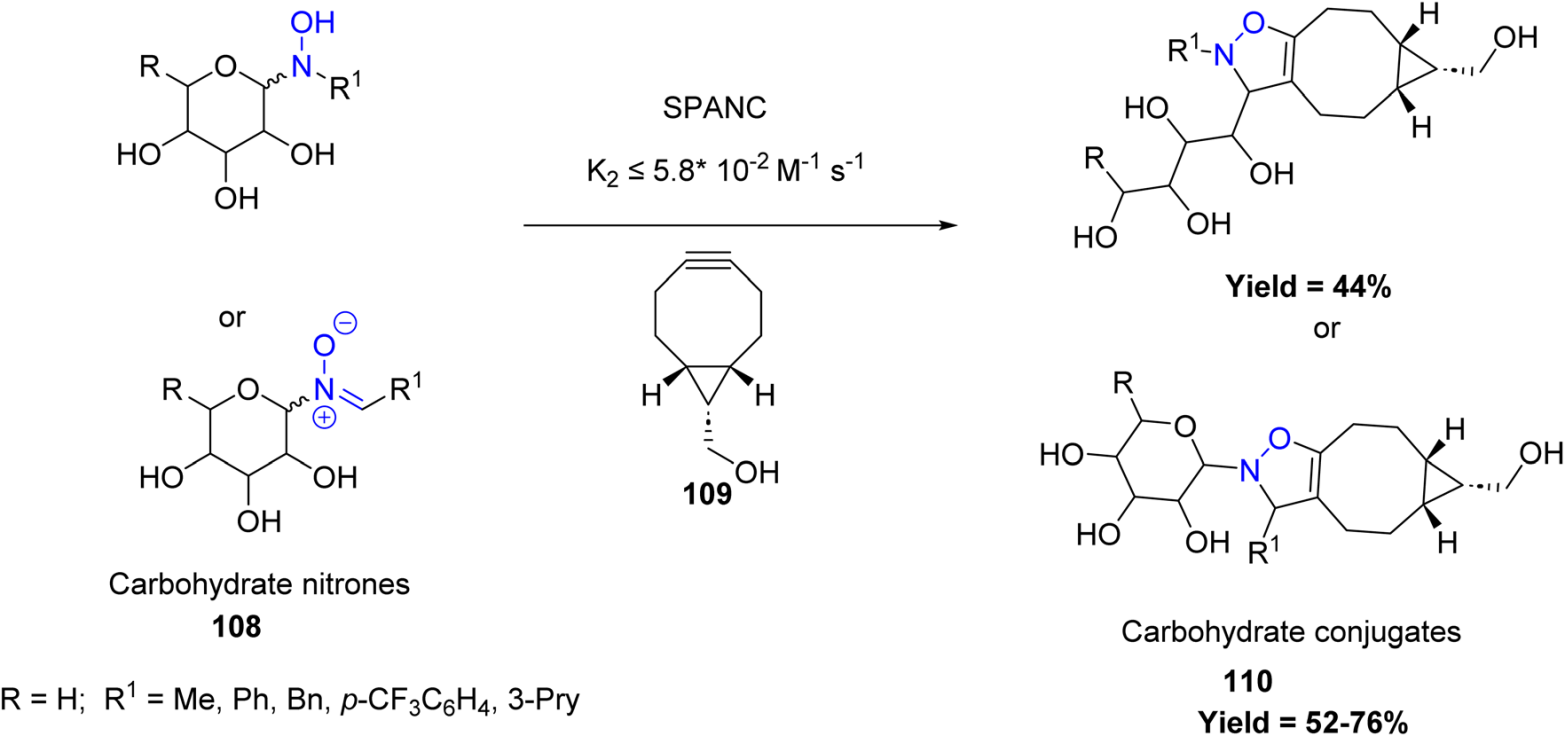
[3 + 2] cycloaddition of unprotected carbohydrate-based nitrones with strain-promoted alkyne.

Expanding into carbohydrate chemistry, Jean-Bernard Behr *et al.*, in 2024 described an efficient heat promoted cycloaddition of unprotected carbohydrate-based nitrones 108 with strain-promoted alkyne to give isoxazolidines 110 in good yields. This is a new tool for bioconjugation. The reaction also studied in aqueous media and gives good yield of product. However, the method is limited by the slow reactivity of certain carbohydrate-based nitrones and the instability of electron-rich or *N*-aryl nitrones under the reaction conditions (Scheme 26).^73^

**Scheme 27:**
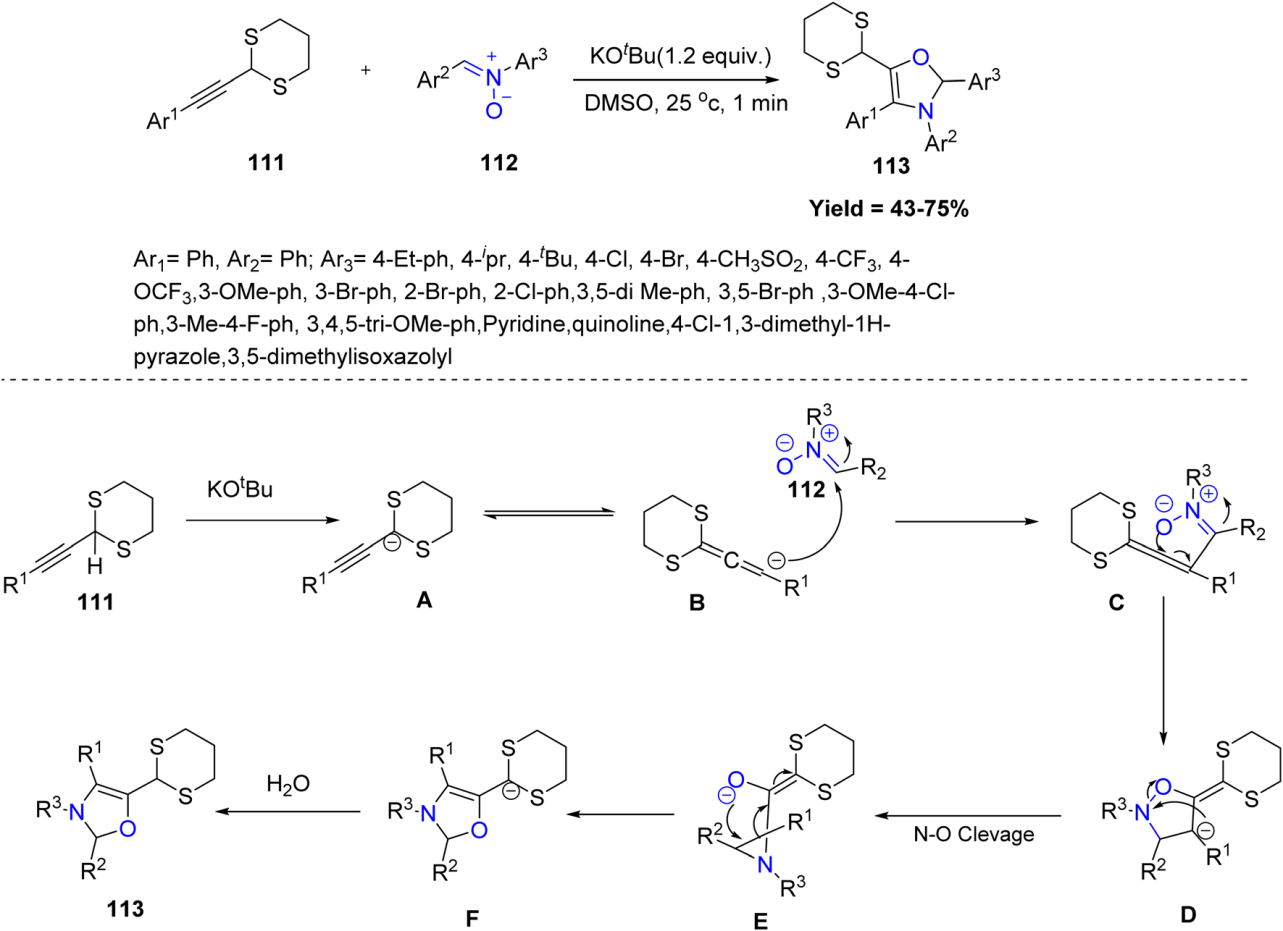
[3 + 2] cycloadditions of alkynyl-1,3-dithianes and nitrones.

In the same year, Shouchu Tang *et al.*, in 2024 reported a regioselective [3 + 2] cycloaddition and umpolung strategy of 1,3-dithianyl alkynes 111 with nitrones 112, in presence of KO-^*t*^Bu, yielding rearranged 2,3-dihydrooxazole derivatives with moderate to high yields. A broad range of aryl- and *N*-substituted nitrones was tolerated, while alkyl nitrones and certain alkyl-dithiane derivatives failed, indicating substrate limitations. Gram-scale reactions confirmed practicality, and the products could be further transformed into esters, amides, and α-amino carbonyl derivatives, demonstrating synthetic versatility. A plausible mechanism study reveals that, initially, alkynyl-1,3-dithiane undergoes rapid deprotonation in the presence of KO-^*t*^Bu, transforming in to an allene anionic intermediate. Subsequently, nucleophilic addition occurs between the allene anion B and the electrophilic nitrone 112, forming allene intermediate C and dihydroisoxazole anion D through an anion relay process. Cleavage of the N–O bond in anion D leads to the formation of aziridine E. Finally, the aziridine ring opens, and the capture of the dithianyl anion with H_2_O yields the desired 2,3-dihydrooxazole product 113 (Scheme 27).^74^

**Scheme 28:**
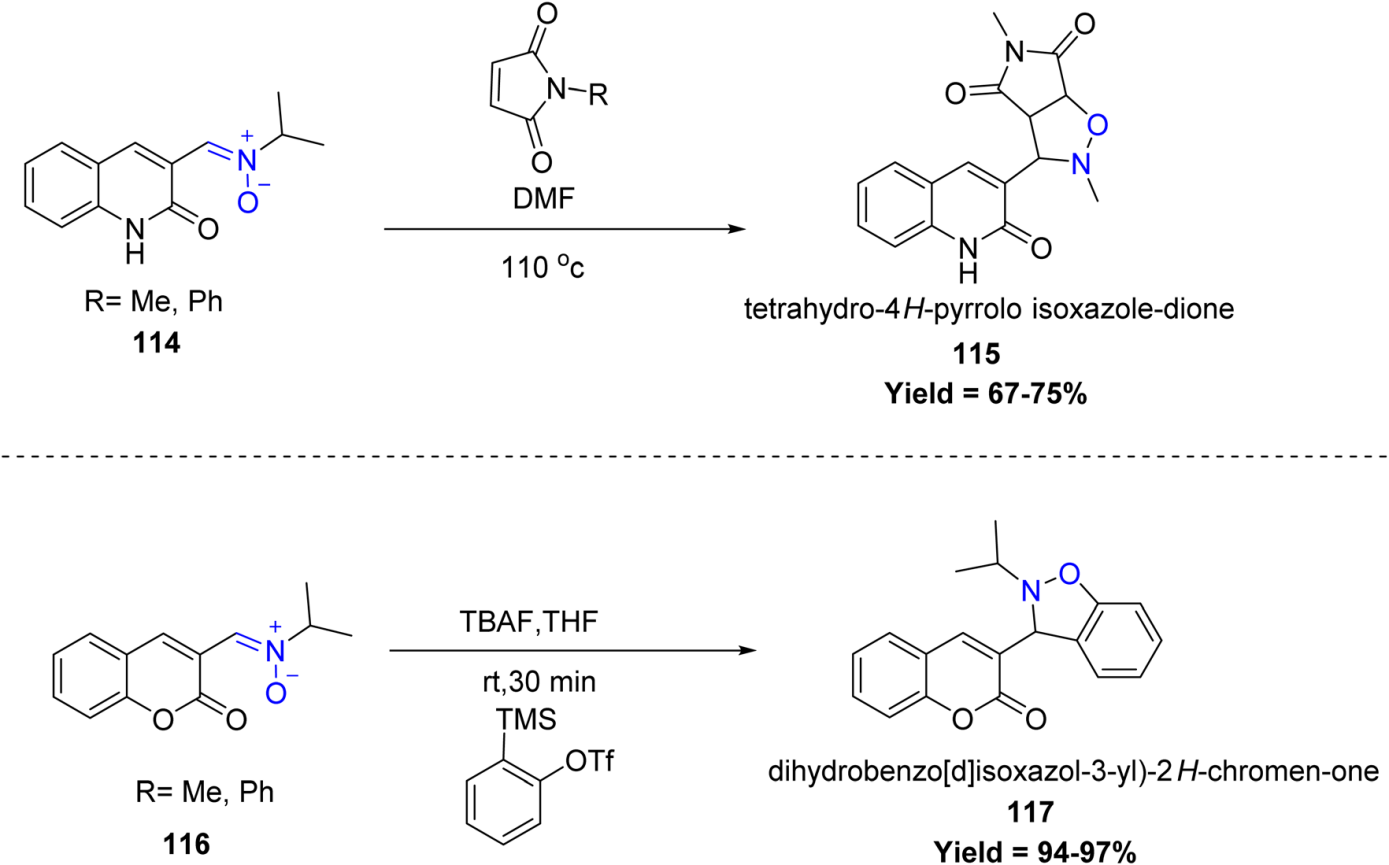
[3 + 2] reaction of quinolinoneyl nitrones and coumarinyl nitrones with maleimide and PhOTfTMS.

Longjia Yan *et al.*, in 2024 reported a mild and efficient method for the synthesis of quinolinonyl 115 and coumarinyl-nitrone 117 derivatives. The reaction was carried out between *N*-(2-formylphenyl) propiolamide/2-formylphenyl propiolate and *N*-methylhydroxylamine hydrochloride under an argon atmosphere at room temperature. The resulting quinolinonyl nitrone underwent a [3 + 2] cycloaddition with maleimide, affording tetrahydro-4*H*-pyrrolo[3,4-*c*]isoxazole-dione derivatives 115. Meanwhile, the coumarinyl nitrone reacted with PhOTfTMS in the presence of TBAF, undergoing a similar [3 + 2] cycloaddition to form 3-(2-isopropyl-2,3-dihydrobenzo[*d*]isoxazol-3-yl)-2*H*-chromen-4-one derivatives 117 in excellent yield. However, the method is limited by sterically hindered or strongly electron-withdrawing substituents, which significantly lower the yields, and certain benzyl/alkyl hydroxylamines or terminal alkynes fail to give the desired nitrones (Scheme 28).^75^

**Scheme 29:**
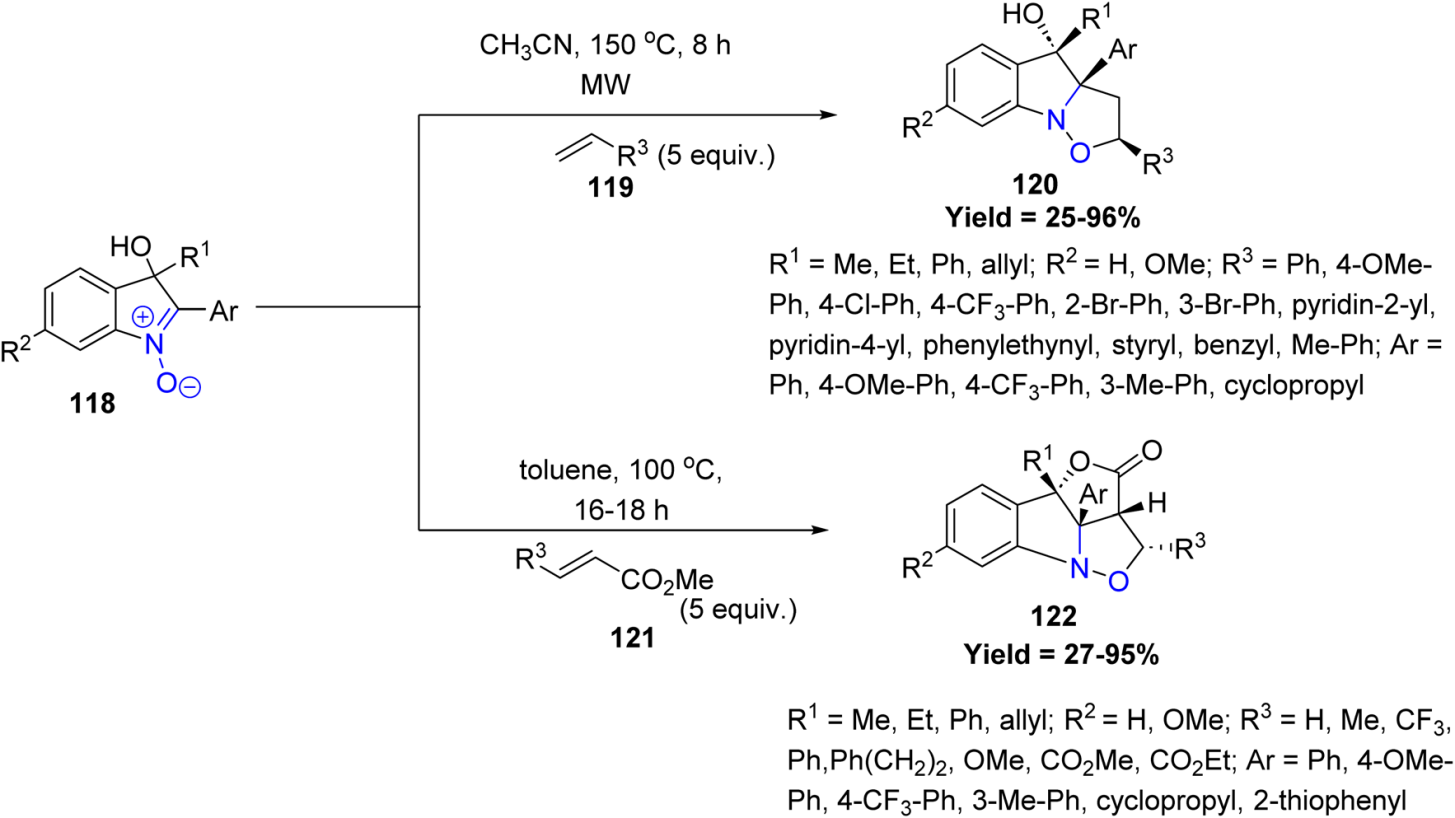
Regio- and stereoselective [3 + 2] cycloaddition using isatogenol.

Similarly, Shigeru Arai *et al.* in 2024 disclosed a regio- and stereoselective [3 + 2] cycloaddition strategy for the construction of fully substituted indoline frameworks 120 and 122, demonstrating the synthetic utility of isatogenol 118 as a key building block. This method enables the rapid formation of densely functionalized, multiring-fused heterocycles bearing contiguous tetra-substituted carbon centers in a single step. Electron-rich aryl groups on isatogenol show higher reactivity, while electron-withdrawing or sterically hindered substituents give lower yields. A key limitation is the reduced selectivity or lower reactivity with sterically hindered or α-substituted alkenes. DFT studies further elucidated that the observed regio- and stereoselectivity is predominantly governed by specific hydrogen-bonding interactions during the cycloaddition with acrylate partners. This work highlights a powerful and elegant approach toward structurally complex indoline derivatives (Scheme 29).^76^

**Scheme 30:**
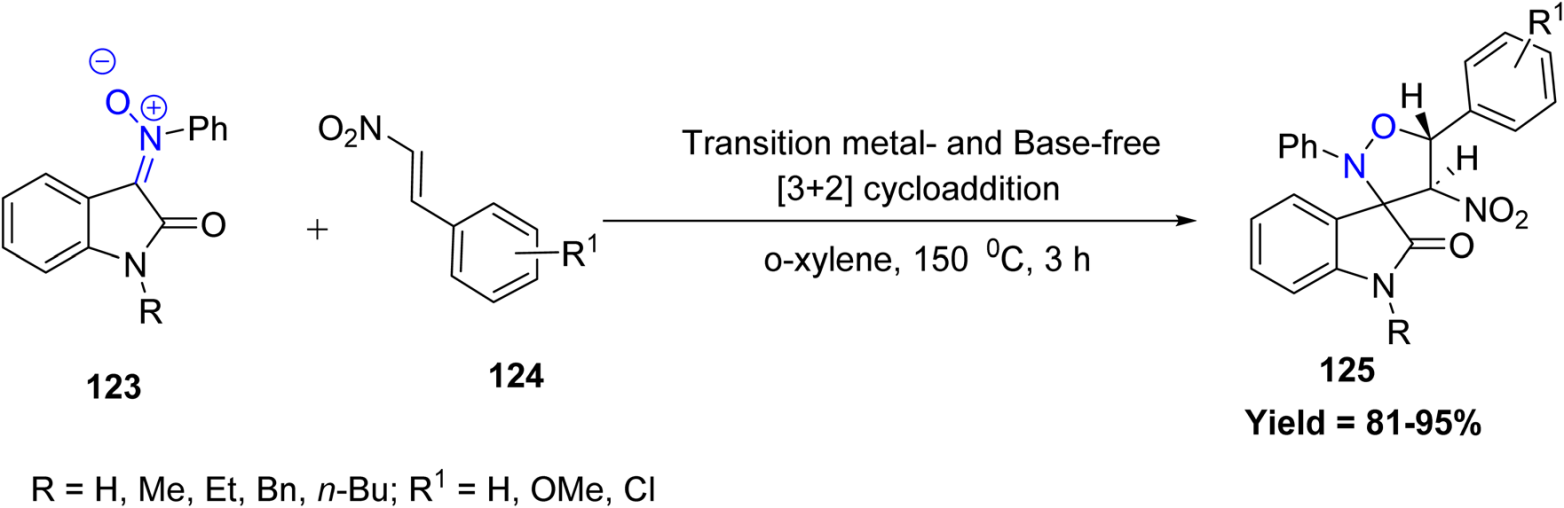
[3 + 2] cycloaddition reaction of isatin ketonitrone derivatives with β-nitrostyrene.

Highlighting the advantages of transition-metal-free strategies, Nayak *et al.* reported a transition metal- and base-free [3 + 2] cycloaddition of isatin ketonitrones 123 with β-nitrostyrene 124 to efficiently construct 4′-nitro-2′,5′-diphenyl spiro[indoline-3,3′-isoxazolidin]-2-one derivatives 125. The reaction proceeds smoothly with a variety of substituted β-nitrostyrenes, and both electron-donating and electron-withdrawing groups on the aromatic ring show minimal influence on product yield. Reactivity trends revealed that *N*-alkyl-substituted nitrones, particularly *N*-methyl, *N*-ethyl, *N*-butyl, and *N*-benzyl variants, significantly enhance the reaction efficiency, providing higher yields relative to unsubstituted nitrones. However, the methodology shows limitations with sterically congested or strongly electron-rich/electron-poor styrenes, as di- and tri-methoxy as well as nitro-substituted styrenes failed to undergo cycloaddition. The antimicrobial activity of all synthesized compounds was evaluated against a panel of Gram-positive and Gram-negative pathogens (Scheme 30).^77^

**Scheme 31:**
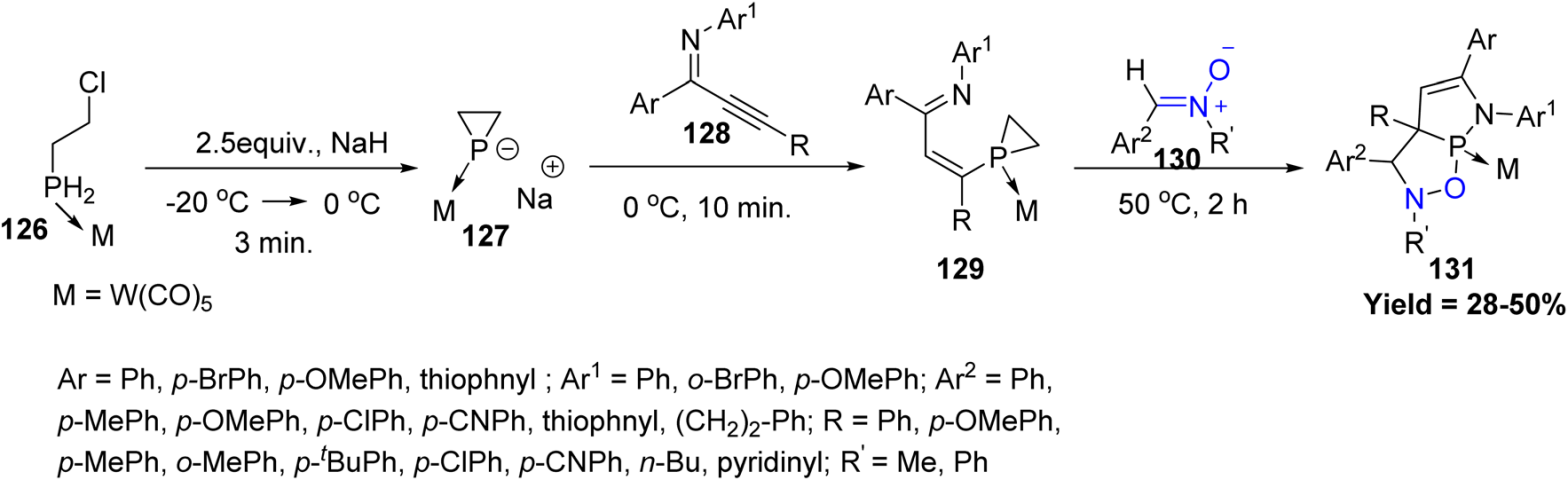
[3 + 2] cycloaddition of P-heteroarenes and nitrones.

Dearomative cycloaddition of heteroarenes has appeared as one of the simple and powerful strategies to access polyheterocyclic skeletons. In this connection Zheng Duan *et al.* assembles simple β-chloroethyl-phosphane 126, alkynyl imines 128 (or alkynyl ketones), and nitrones 130 into structurally complex isoxazolidine fused phospholene scaffolds 131*via* a sequential process involving phospha-Michael addition, intramolecular cyclization, and dearomatizing [3 + 2] cycloaddition reactions. The method tolerates both electron-rich and electron-poor aryl substituents, whereas sterically hindered or strongly electron-donating groups lead to reduced yields. Electron-donating nitrones display higher reactivity, while *N*-aryl nitrones remain unreactive. This strategy also extends to ynones, enabling access to oxaphospholene analogues and potential P-stereogenic ligand frameworks. Unlike pyrroles and furans, aromatic 2-phosphapyrroles and 2-phosphafurans serve as excellent 2π-components in dearomative [3 + 2] cycloadditions due to the poor 2p–3p orbital overlap within the C=P unit (Scheme 31).^78^

**Scheme 32:**
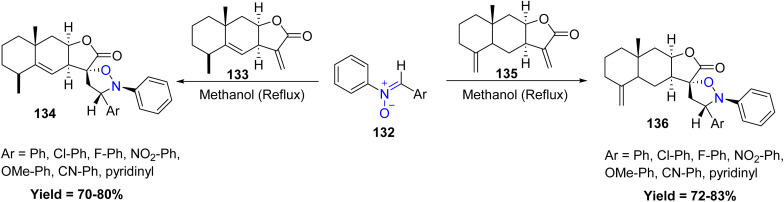
Cycloaddition reactions of nitrone and alantolactone or iso alantolactone.

Yogesh P. Bharitkar *et al.* utilized nitrone cycloaddition to synthesize novel spiroisoxazolidino hybrids 134/136 of alantolactone 133 and isoalantolactone 135. Which are two isomeric sesquiterpene lactones of innula recemosa. This study includes the synthesis of dinitrone with glyoxal and terephthalaldehyde. Both nitrone cycloaddition and di-nitrone cycloaddition proceed with high regio- and diastereoselectivity, resulting in the formation of only one isomer. Notably, the reactions proceeded under mild conditions without the need for chromatographic purification, highlighting the synthetic efficiency of this approach. The antimicrobial activity of all synthesized compounds was evaluated against a panel of Gram-positive and Gram-negative pathogens. Few compounds show potent activity (Scheme 32).^79^

**Scheme 33:**
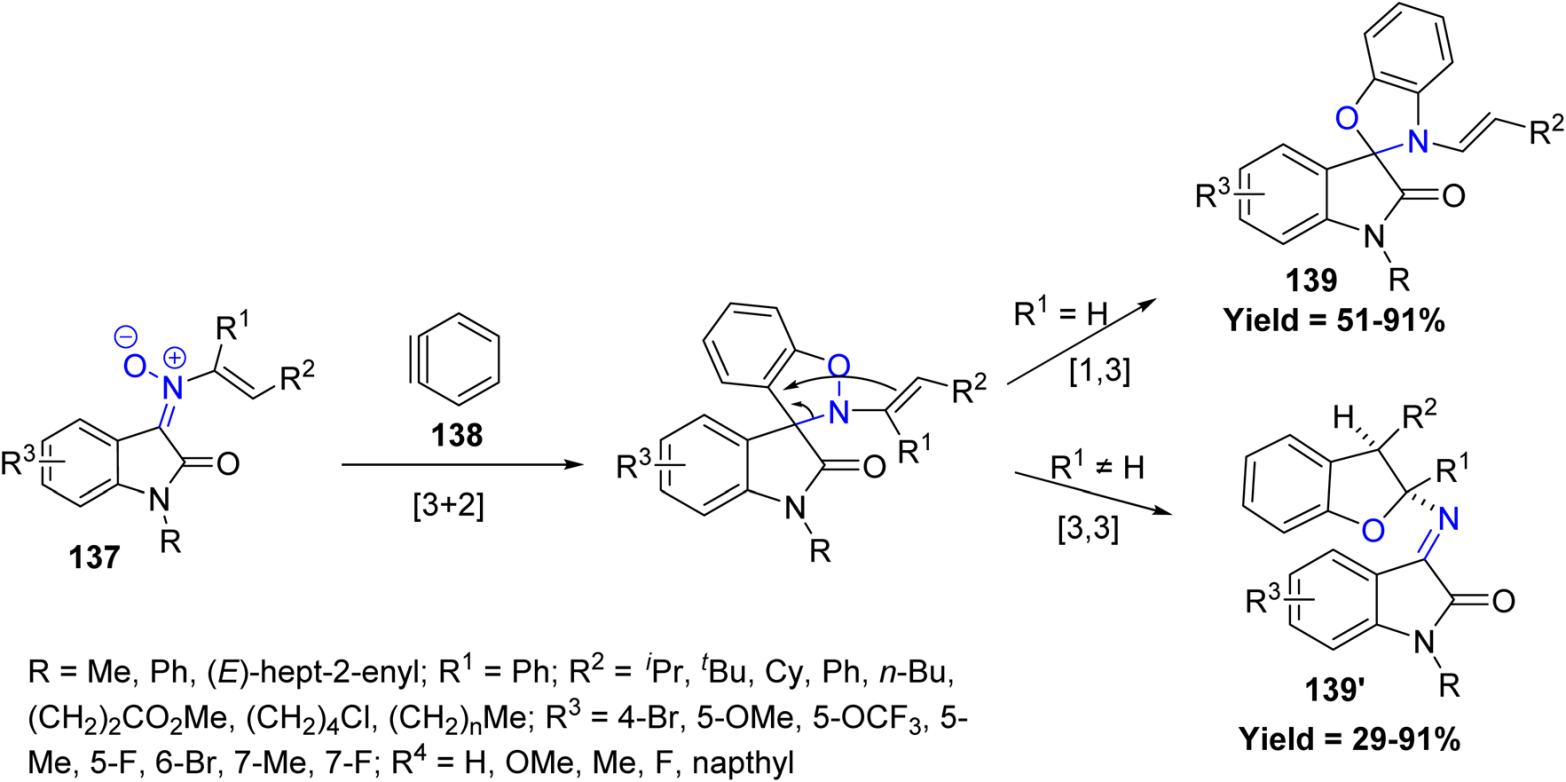
[3 + 2] cycloaddition and selective rearrangement of *N*-vinyl oxindole nitrones and arynes.

Recently, Dong-Liang Mo *et al.* in 2025 developed an efficient, transition metal-free protocol for the synthesis of a diverse array of spirooxindole-benzo[*d*]oxazoles 139 and dihydrobenzofurans 139′. This transformation proceeds *via* a [3 + 2] cycloaddition between *N*-vinyl oxindole-derived nitrones 137 and arynes 138, followed by a selective rearrangement. The nature of the substituent on the *N*-vinyl group of the nitrone plays a critical role, directing either a [1,3]- or [3,3]-sigmatropic rearrangement, primarily influenced by steric effects. The monosubstituted linear *N*-vinyl nitrones gave high yields regardless of chain length, whereas sterically bulky or disubstituted nitrones led to lower yields or favoured formation of dihydrobenzofurans over spirooxindoles. Substituents on the indole ring, whether electron-donating or electron-withdrawing, were generally tolerated, and sensitive functional groups such as chloro and ester were compatible. However, limitations were observed with highly hindered *N*-vinyl nitrones or naphthyl-derived arynes, which either decomposed or gave reduced yields, indicating that steric hindrance can significantly affect reaction efficiency and selectivity (Scheme 33).^80^

**Scheme 34:**
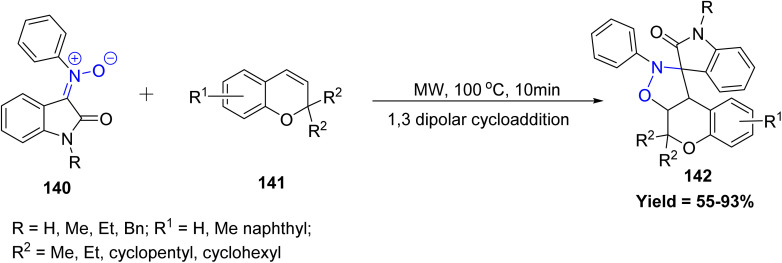
[3 + 2] cycloaddition of 2*H*-chromene and *N*-arylisatin nitrones.

In a significant contribution to green synthetic methodologies, Nayak *et al.* developed an efficient aqueous-mediated approach for the synthesis of chromene-fused spiro-isoxazolidine derivatives 142*via* a [3 + 2] cycloaddition reaction. Utilizing 2*H*-chromenes 141 and *N*-arylisatin-derived nitrones 140, the reaction proceeds under microwave irradiation, offering notable advantages such as reduced reaction times, minimized by-product formation, and excellent yields. Reactivity trends indicated that substitution at the 2-position of the chromene core significantly influenced yields, with cyclopentyl and cyclohexyl substituents giving higher yields compared to 2,2-dimethyl or 2,2-diethyl groups. Similarly, larger substituents on the nitrone (methyl < ethyl < benzyl) slightly improved reaction efficiency. However, limitations were observed with certain C3- and C4-substituted chromenes, such as 4-bromo, 4-styryl, or carbonyl/nitro-substituted derivatives, which either gave complex mixtures or failed to react, highlighting the sensitivity of this cycloaddition to steric and electronic effects (Scheme 34).^81^

#### 2.1.2 [4 + 2] hetero-Diels–Alder reactions

**Scheme 35:**
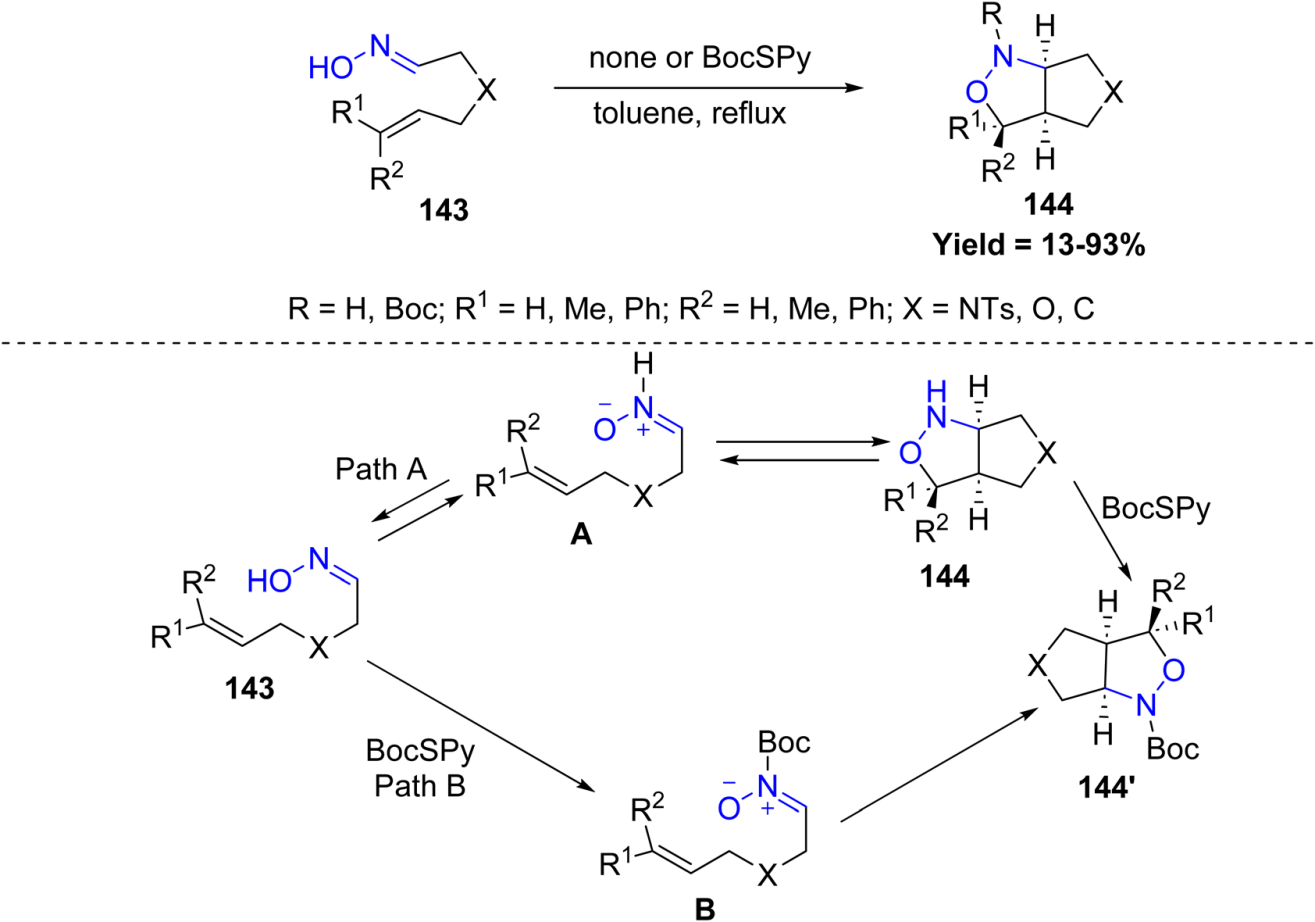
[4 + 2] intramolecular cycloaddition of *N*-alkoxycarbonyl nitrones.

In 2023, Osamu Tamura *et al.*, reported an elegant strategy for constructing *N*-alkoxycarbonylated polycyclic compounds 144 through an intramolecular nitrone cycloaddition. This method involves the thermal treatment of δ,ε-unsaturated oximes bearing a alkene groups with *O*-alkyl *S*-(pyridin-2-yl) carbonothioates (PySCO_2_R), resulting in the *in situ* formation of *N*-alkoxycarbonyl nitrones. These intermediates then undergo intramolecular [3 + 2] cycloaddition to furnish multi-cyclic frameworks. Reactivity trends revealed that substrates bearing quaternary centers or dimethyl-substituted alkenes delivered higher yields compared to less substituted analogues, while simple heating in the absence of BocSPy often led to poor conversion or recovery of starting materials. Two mechanistic pathways were proposed: in Path A, the oxime 143 first equilibrates with a transient NH-nitrone A, which cyclizes to form 144, this 144 is then acylated by the PySCO_2_R to form 144′. In Path B, direct acylation of the oxime 143 leads to the formation of an N-Boc nitrone B, which subsequently undergoes cycloaddition 144′. Both pathways effectively demonstrate the utility of *in situ* nitrone generation for rapid construction of complex heterocyclic compounds (Scheme 35).^82^

**Scheme 36:**
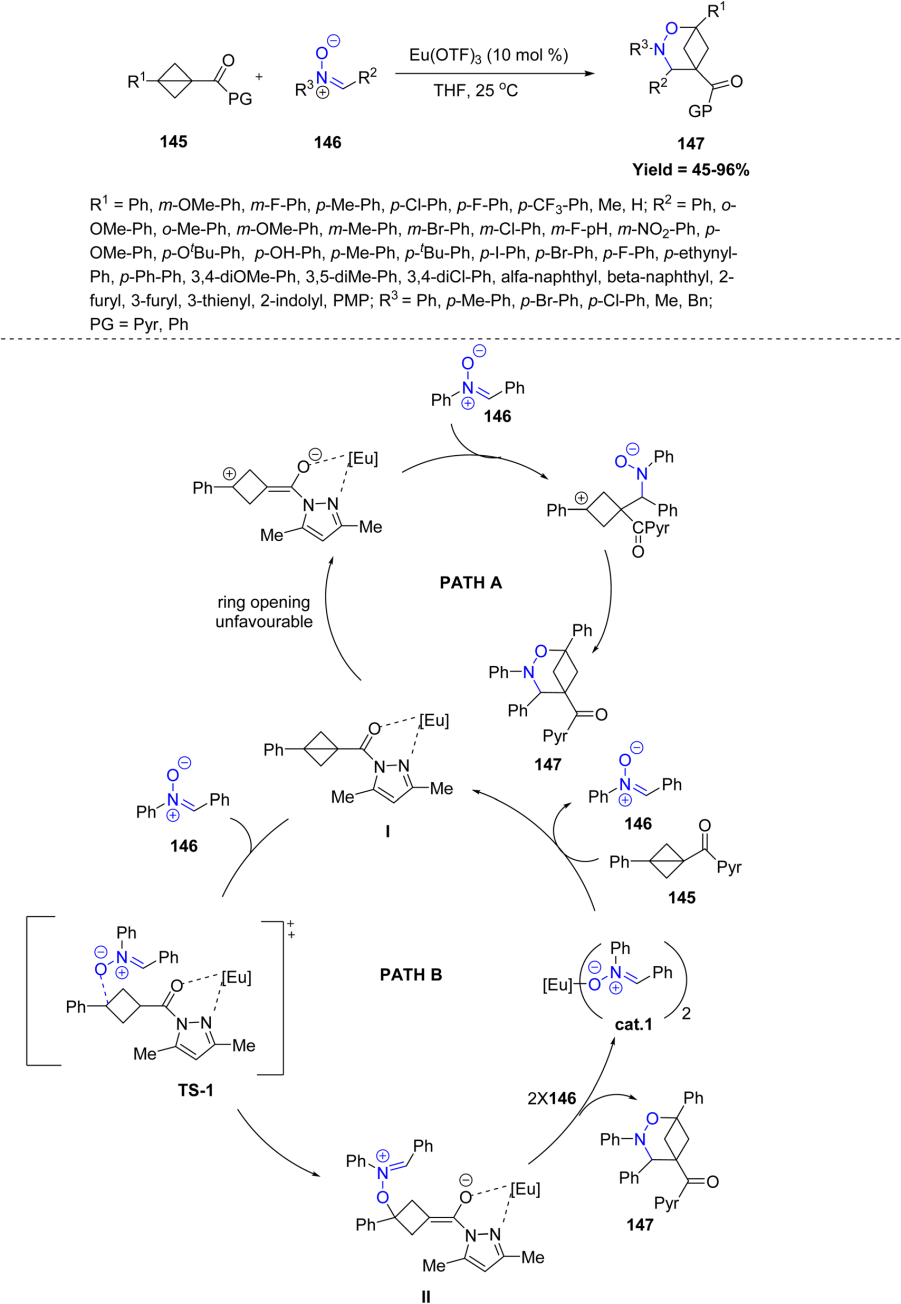
Formal dipolar [4 + 2] cycloaddition of bicyclo-[1.1.0]butanes with nitrones.

Similarly, Wei-Ping Deng *et al.* in 2024 reported a strategy for synthesizing valuable hetero-BCHeps 147*via* dipolar cycloadditions of bicyclo[1.1.0] butanes 145 (BCBs) with hetero-1,3-dipoles 146 under mild conditions, showcasing broad functional group tolerance. The method displays broad substrate tolerance: BCBs bearing electron-rich, electron-poor, or sterically varied aryl substituents reacted smoothly to deliver the corresponding products in moderate to excellent yields. Similarly, nitrones with diverse electronic and steric properties including mono- and disubstituted aryl, heteroaryl, and naphthyl groups were well accommodated, whereas strongly aliphatic nitrones showed reduced reactivity. Mechanistically, the transformation proceeds *via* a formal [4π + 2σ] cycloaddition reaction and proceeds through two pathway. In pathway-1, the nitrone first activates the BCB, followed by ring closure to afford the final product 147. Alternatively, in the second pathway, intermediate I reacts with compound 146 to form transition state TS-1, which leads to intermediate II. This intermediate then eliminates the catalyst to produce the final compound 147. Then the catalyst reacts with compound 145 to reform compound 146 (Scheme 36).^83^

#### 2.1.3 [2 + 2] cycloaddition

**Scheme 37:**
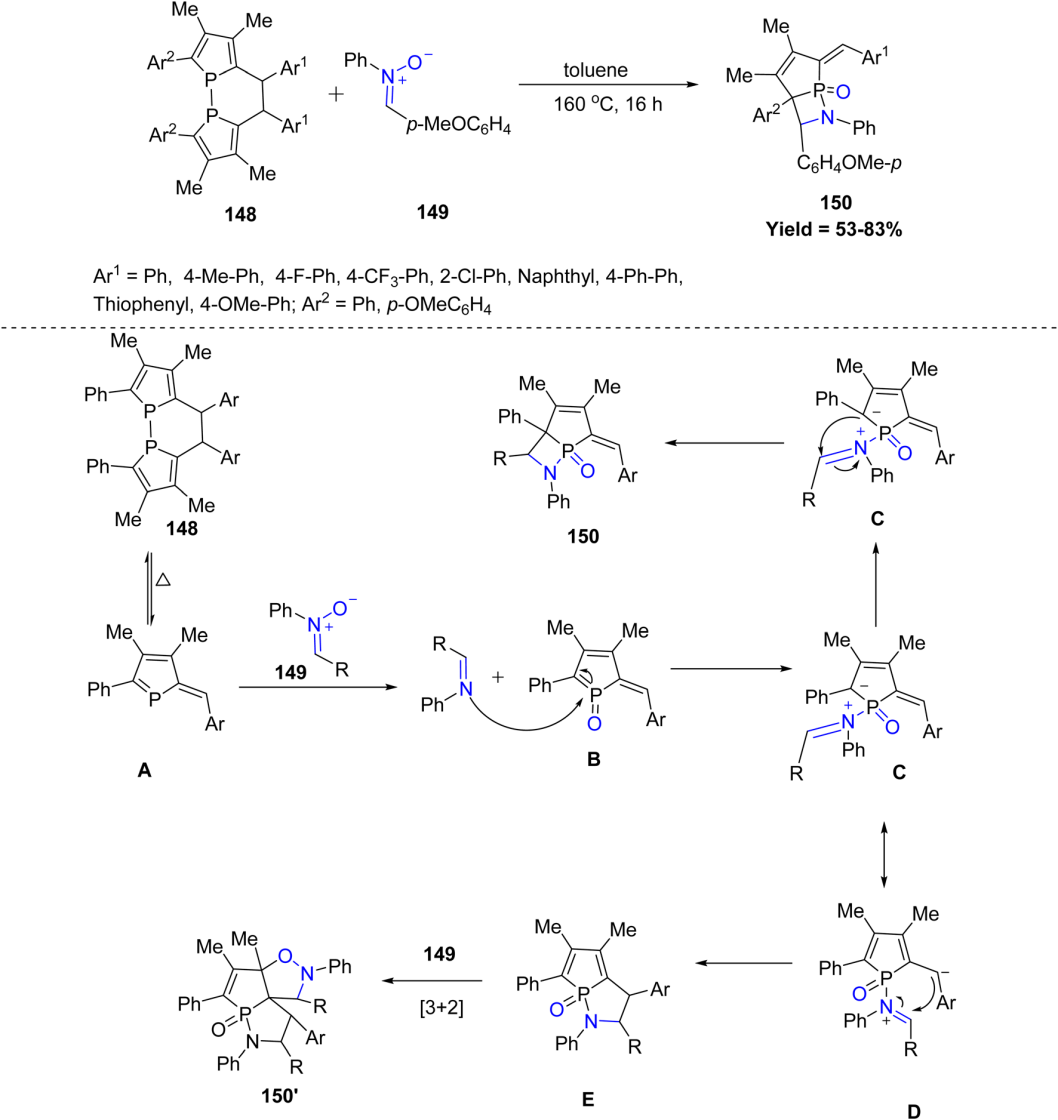
[4 + 2] cycloaddition reaction of α-C2-bridged biphospholes and nitrones.

An unprecedented reaction of nitrone-based [2 + 2] cycloaddition was reported in 2024 by Rongqiang Tian *et al.*, involving *in situ* generated 1-phosphafulvene 148 and nitrones 149 to give phospholene-fused β-phosphinolactam derivatives 150. The reaction works best with nitrones that are not strongly electron-withdrawing or sterically bulky, because such groups slow down the redox step and decrease product formation. A key limitation of the method is that strongly hindered or highly electron-poor nitrones give much lower yields or do not react efficiently. Mechanistically, biphosphole 148 first undergoes dissociation to generate 1 phosphafulvene A. The redox reaction between A and nitrone 149 provides 1-phosphafulvene oxide B and imine. The oxidation of 1-phosphafulvene boosts its reactivity toward imine. The nucleophilic addition of imine to B affords intermediate C. The cyclization of C produces β-phosphinolactam, leading to the isolated product 150. The negative ion of C shifts to exocyclic carbon atom results in intermediate D. The instability of phosphole oxide caused by slight antiaromaticity disfavours the negative ion migration from C to D. The intramolecular nucleophilic addition of carbanion to iminium ion produces E. The subsequent [3 + 2] cycloaddition reaction between phosphole oxide and nitrone phospholene 149 provided fused isoxazolidine 150′ (Scheme 37).^84^

### 2.2 Acid-catalysed pathways

**Scheme 38:**
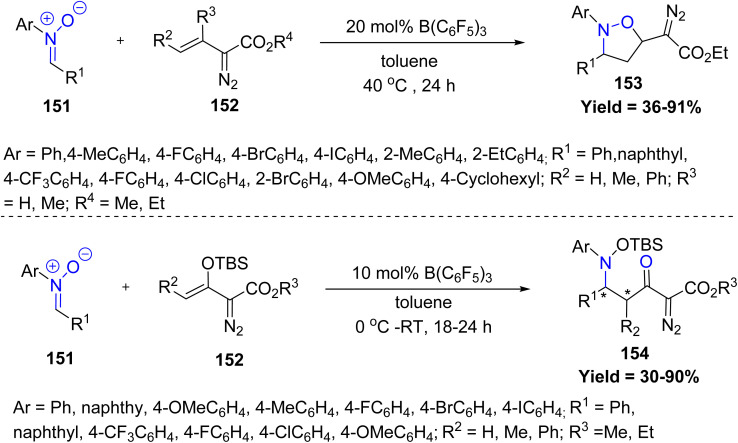
B(C_6_F_5_)_3_-Catalyzed diastereo selective and divergent reactions of vinyldiazo esters with nitrones.

Acid catalysis has emerged as a versatile strategy in nitrone-based heterocyclic synthesis, providing efficient, metal-free alternatives to transition-metal-catalyzed transformations. In 2023, Rebecca L. Melen and co-workers reported a transition-metal-free, Lewis acid-catalyzed reaction for the diastereoselective synthesis of highly functionalized isoxazolidine-derived diazo compounds 153 in excellent yields. Reactivity mainly depends on the strong Lewis acidity and steric features of B(C_6_F_5_)_3_, along with the electronic nature of the nitrone. However, electron-rich or bulky nitrones, *N*-alkyl nitrones, internal vinyldiazo esters, and coordination-prone solvents reduce or prevent reactivity (Scheme 38).^85^

**Scheme 39:**
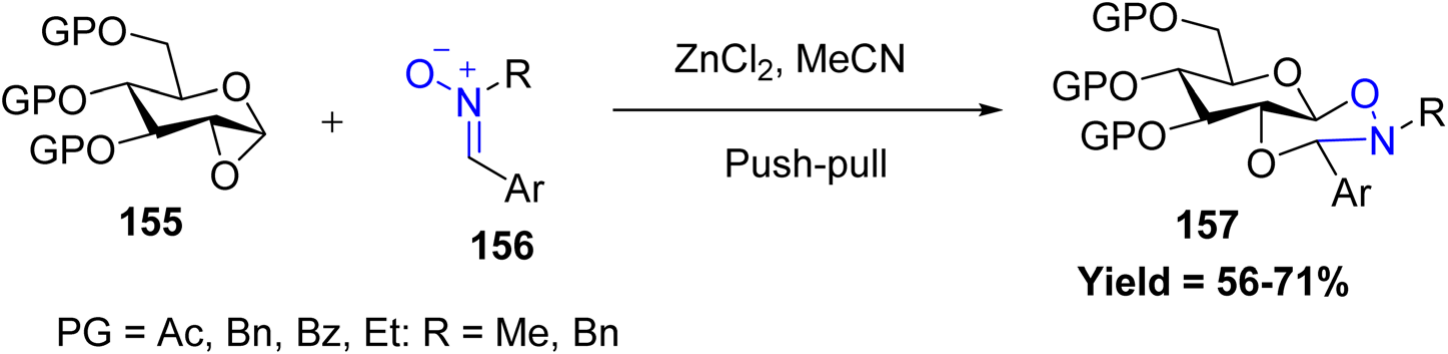
Zinc chloride catalyzed reaction of 1,2-anhydro sugars and *N*-substituted aromatic nitrones.

In this context, Debaraj Mukherjee *et al.* reported a zinc chloride-catalyzed, one-pot, diastereoselective approach for the synthesis of sugar-fused dioxazinane derivatives starting from 1,2-anhydro sugars 155 and *N*-substituted aromatic nitrones 156. These 1,2-annulated pyranose frameworks, fused with six-membered heterocycles, represent a valuable class of carbohydrate scaffolds 157 with significant synthetic and biological relevance. The methodology exhibits broad substrate tolerance, accommodating diverse aromatic nitrones with electron-donating and electron-withdrawing substituents, and is compatible with both ester- and ether-protected sugars. Mechanistically, ZnCl_2_ activates the anhydrosugar 155, promoting nucleophilic attack by the nitrone 156, followed by an intramolecular cyclization to furnish the fused dioxazinane products 157 (Scheme 39).^86^

**Scheme 40:**
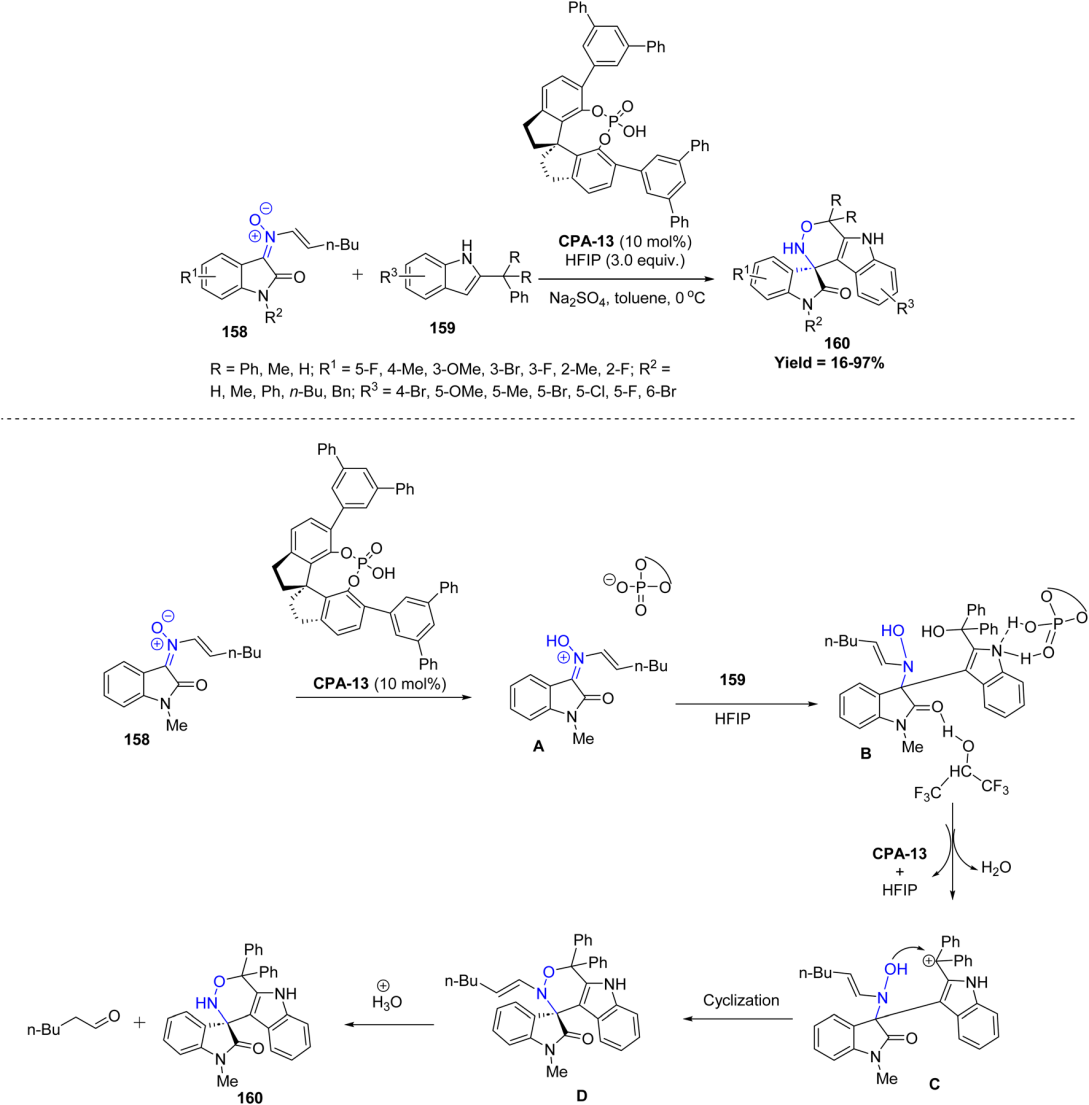
Chiral phosphoric acid (CPA) catalyzed asymmetric [3 + 3] cycloaddition of *N*-viny oxindole nitrones with 2-indolylmethanols.

In 2024, Wen-Jun Zhou *et al.* introduced an enantioselective strategy for constructing spirooxindole[1,2]oxazine frameworks 160*via* a chiral phosphoric acid (CPA)-catalyzed [3 + 3] cycloaddition between *N*-vinyl oxindole nitrones 158 and 2-indolylmethanols 159. The substrate scope is reasonably broad, allowing a variety of aromatic, heteroaromatic, and moderately substituted starting materials to participate effectively. However, the method still shows certain limitations, such as reduced efficiency with highly bulky substrates, intolerance toward strongly electron-withdrawing groups, and occasional formation of side products under harsh conditions. The proposed mechanism suggests initial activation of nitrone 158 by CPA-13, leading to intermediate A. Subsequent reaction with 159 in HFIP facilitates formation of intermediate B, which undergoes dehydration to yield intermediate C, concurrently releasing the catalyst and solvent. An intramolecular cyclization of C furnishes intermediate D, which upon hydrolytic cleavage of the enamine moiety under acidic aqueous conditions, results in the formation of the spirooxindole[1,2]oxazine product 160 along with 1-hexanal (Scheme 40).^87^

### 2.3 Redox and photochemical approaches

**Scheme 41:**
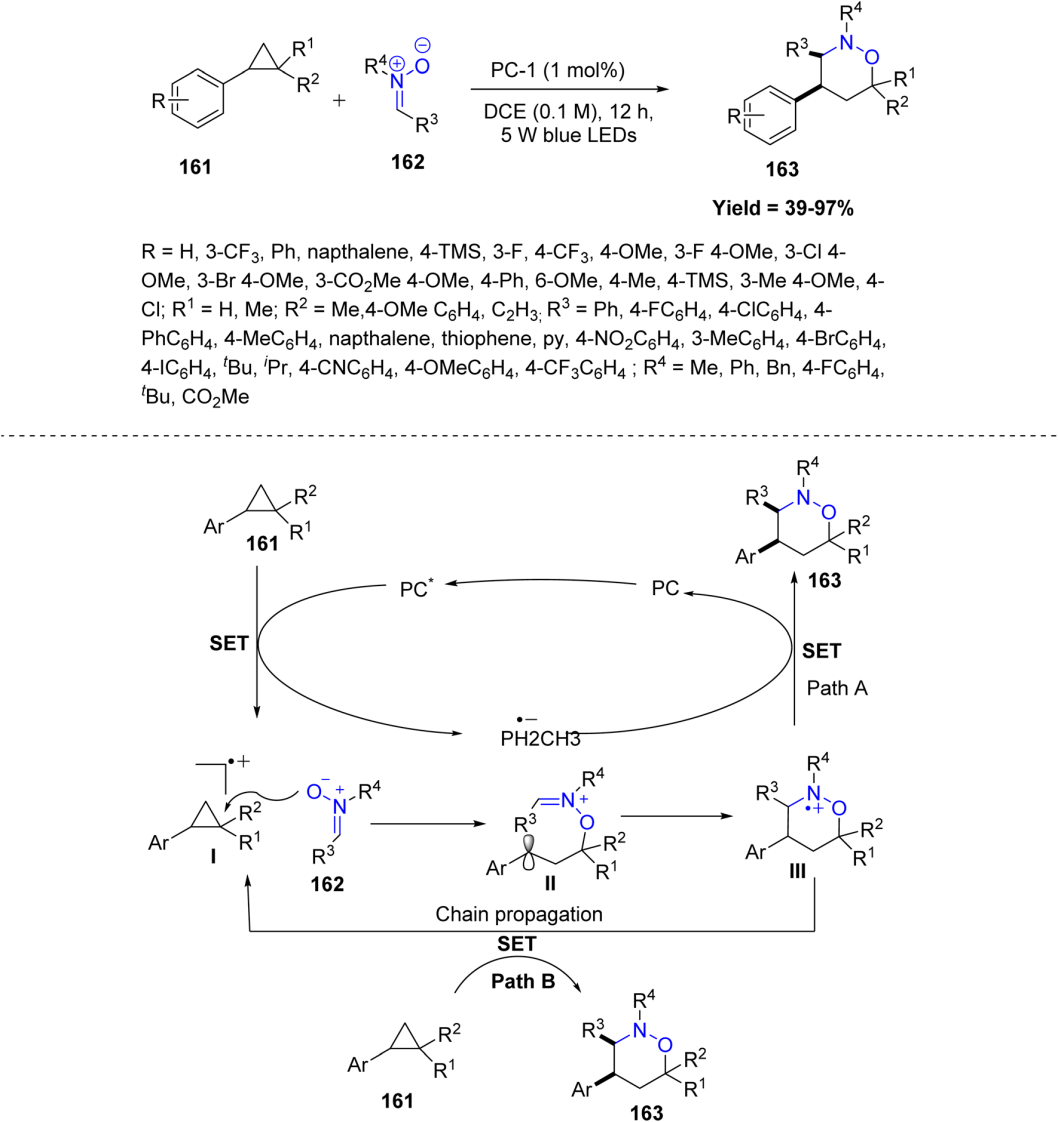
Photoredox-catalyzed [3 + 3] dipolar cycloaddition of nitrones with aryl cyclopropanes.

Chao Feng and co-workers in 2023 reported a novel visible-light-driven, photoredox-catalyzed [3 + 3] dipolar cycloaddition of nitrones 162 with aryl cyclopropanes 161 for the synthesis of complex six-membered heterocycles 163. Mechanistically, the reaction is initiated by the excitation of an acridinium photocatalyst under blue light (450 nm), which undergoes single-electron transfer (SET) with the aryl cyclopropane 161 to generate a radical cation I. The nitrone 162 then engages in a regio- and stereoselective nucleophilic attack on this radical cation, promoting ring opening and forming a benzylic radical intermediate II. This intermediate subsequently undergoes a 6-*endo*-trig radical cyclization to form a new C–C bond, delivering a second radical intermediate III. A final SET from the reduced photocatalyst regenerates the ground-state catalyst and completes the catalytic cycle, furnishing the cycloadduct 163 with high diastereoselectivity. Reactivity trends indicate that nitrone attack occurs at the more electron-rich cyclopropyl carbon, and substrates capable of easy SET oxidation react best. Limitations arise with sterically bulky or strongly electron-withdrawing substrates, and cyclopropanes that cannot undergo SET remain unreactive (Scheme 41).^88^

**Scheme 42:**
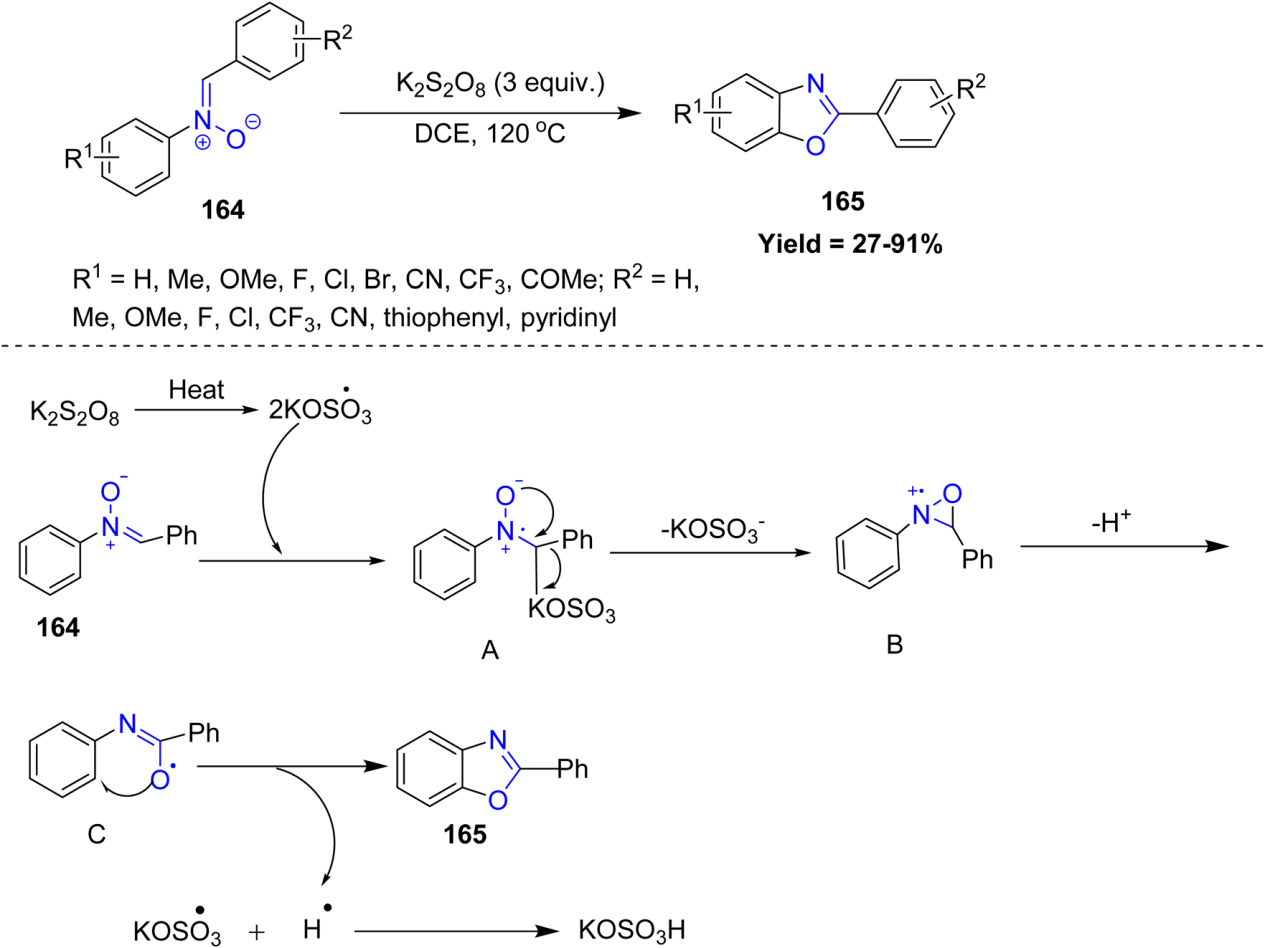
Self-oxidative cyclization of nitrone compound.

Baohua Chen *et al.* reported a highly efficient and environmentally friendly strategy for the synthesis of valuable benzoxazole derivatives *via* a self-oxidative cyclization pathway involving N–O bond cleavage. This metal-free protocol utilizes readily accessible nitrones 164 as starting materials and proceeds smoothly under simple, one-pot conditions. This metal-free oxidative cyclization shows broad functional-group tolerance, with most electron-rich and electron-poor *N*-aryl nitrones reacting smoothly. Reactivity is largely unaffected by electronics, but strong electron-withdrawing groups such as CF_3_ and CN suppress radical formation and give no product. Aryl aldehydes work well, alkenyl and aliphatic aldehydes react poorly, marking the main limitations of this otherwise efficient and green protocol. Mechanistically, the reaction is initiated by the formation of sulfate radical anions (SO_4_˙^−^) from thermolysis of potassium persulfate (K_2_S_2_O_8_), which abstract a hydrogen atom from the substrate 164 to generate a benzyl radical intermediate A. This radical then undergoes intramolecular cyclization to form a strained tricyclic intermediate B. Subsequent concerted cleavage of the N–O bond, accompanied by proton release, drives the ring opening to yield intermediate C. Finally, C undergoes further oxidation by excess sulfate radicals, giving the final compound 2-aryl-substituted (benzo)oxazole products 165 (Scheme 42).^89^

### 2.4 Miscellaneous approaches

#### 2.4.1 Asymmetric click reactions

**Scheme 43:**
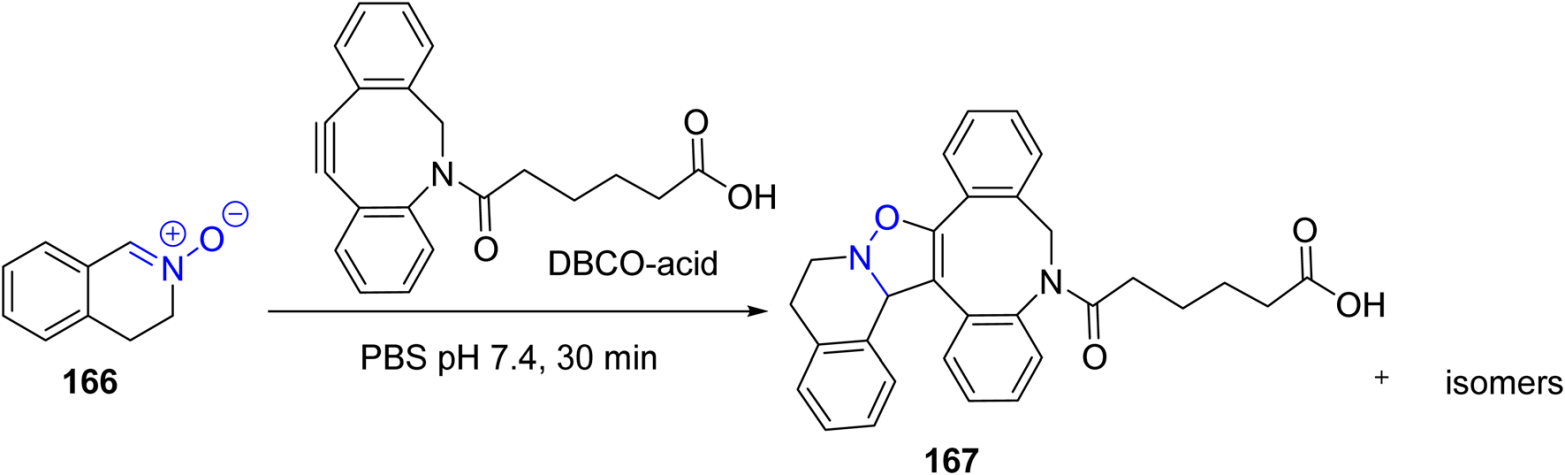
Asymmetric click reaction using cyclic nitrone and strained alkynes.

Tsung-Shing Andrew Wang *et al.* in 2025 reported a strain-promoted alkyne-nitrone cycloaddition (SPANC) using cyclic nitrone 166 and strained alkynes as modular building blocks. This click strategy enabled a convenient 2- and 3-component assembly of multifunctional biomolecules, including PROTACs and biological probes. Their method allows rapid construction of bifunctional and trifunctional degraders, demonstrating efficient in-cell assembly, and the synthesis of caged or trifunctional PROTACs through sequential nitrone formation and SPANC (Scheme 43).^90^

**Scheme 44:**
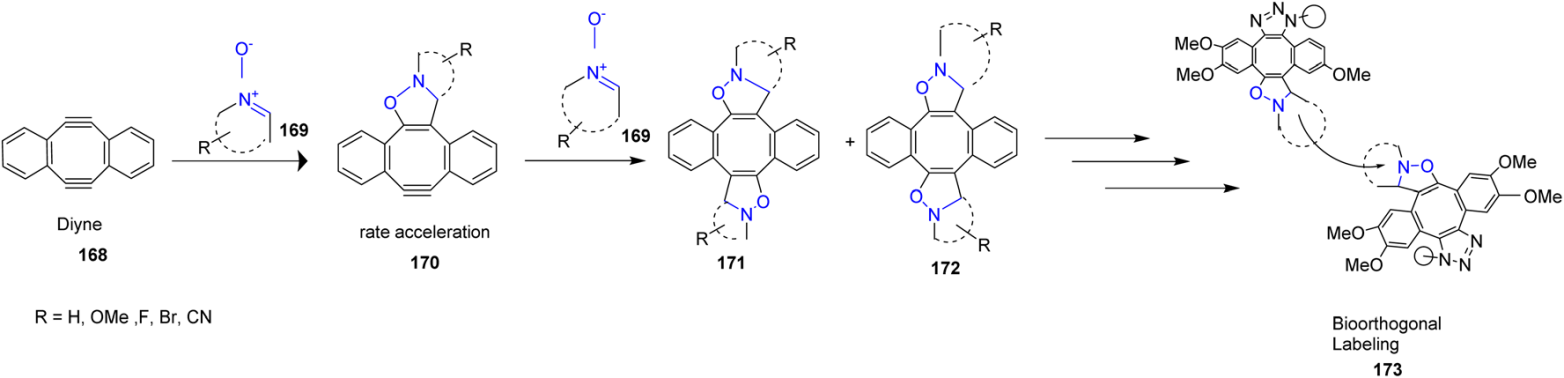
Strain-promoted double-click reaction of nitrone with dibenzocyclooctadiyne.

John Paul Pezacki *et al.* in 2023 demonstrated strain-promoted double-click (SPDC) reaction of nitrone 169 to the strain-promoted dibenzo-cyclooctadiyne 168, highlighting their potential for efficient bioorthogonal labeling. Detailed mechanistic investigations revealed that these transformations share a similar rate-limiting step with dual cycloaddition processes. Examination of the reaction scope and mechanism identified key limitations associated with nitrone cyclooctadiyne cycloadditions, while also providing insights into conditions under which the rate-limiting step can shift (Scheme 44).^91^

#### 2.4.2 Deoxygenative cyclization

**Scheme 45:**
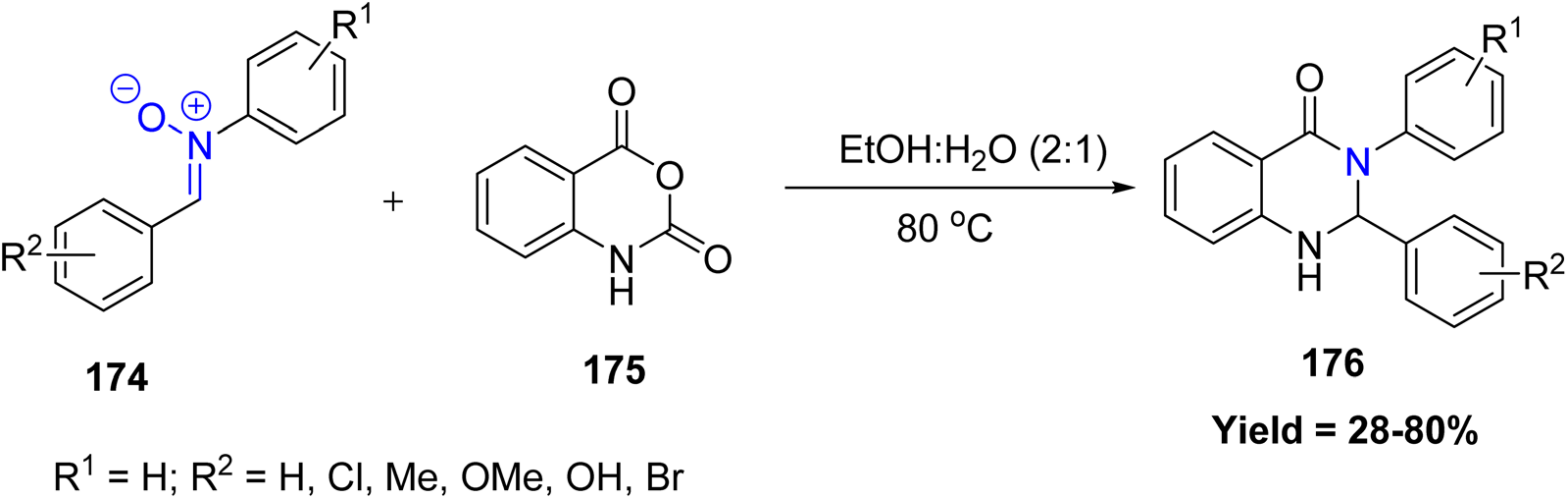
Deoxygenative cycloaddition of nitrones with isatoic anhydride.

Rakesh Kumar *et al.* reported a novel deoxygenative cycloaddition of nitrones 174 with isatoic anhydride 175 for the synthesis of quinazolinones derivatives 176 under metal-free conditions without using any acid or base catalyst. The study shows that nitrones can undergo a deoxygenative cycloaddition in water, making this an efficient and environmentally friendly approach for building useful quinazolinone structures (Scheme 45).^92^

**Scheme 46:**
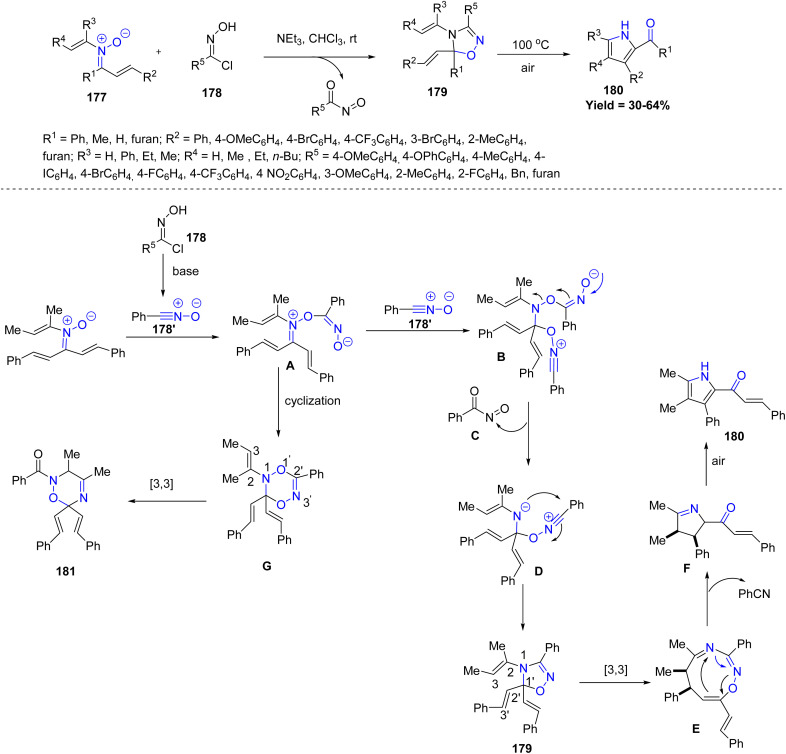
Deoxygenative cyclization cascade reaction between *N*-vinyl-α,β-unsaturated nitrones and nitrile oxides.

Dong-Liang Mo *et al.* in 2023, developed a metal-free deoxygenative cyclization cascade reaction between *N*-vinyl-α,β-unsaturated nitrones 177 and nitrile oxides 178′ for the synthesis of various functionalized 1,2,4-oxadiazolinesand 1,2,5-oxadiazolines 179. Both linear and cyclic *N*-vinyl groups, as well as heteroaryl moieties, were tolerated. Hydroxamoyl chlorides with diverse electronic properties worked efficiently, though bulky groups often reduced yields. Formation of 1,2,5-oxadiazolines required specific solvents and bases, indicating sensitivity to reaction conditions. Mechanistically, the nitrile oxide 178′, generated *in situ* from 178 under basic conditions, reacts with nitrone 177 to afford intermediate A. Subsequent nucleophilic addition of a second nitrile oxide 178′ furnishes intermediate B, which upon elimination yields D. An intramolecular cyclization of D produces the stable 1,5-diene 179, which undergoes a [3,3]-sigmatropic rearrangement to give the nine-membered ring E. Further N–O bond cleavage, elimination, and oxidation of the corresponding intermediate F provide pyrrole 180. Alternatively, intermediate A can undergo intramolecular cyclization to generate G, which through a selective [3,3]-rearrangement affords compound 181 (Scheme 46).^93^

#### 2.4.3 Silylacetate-promoted additions

**Scheme 47:**
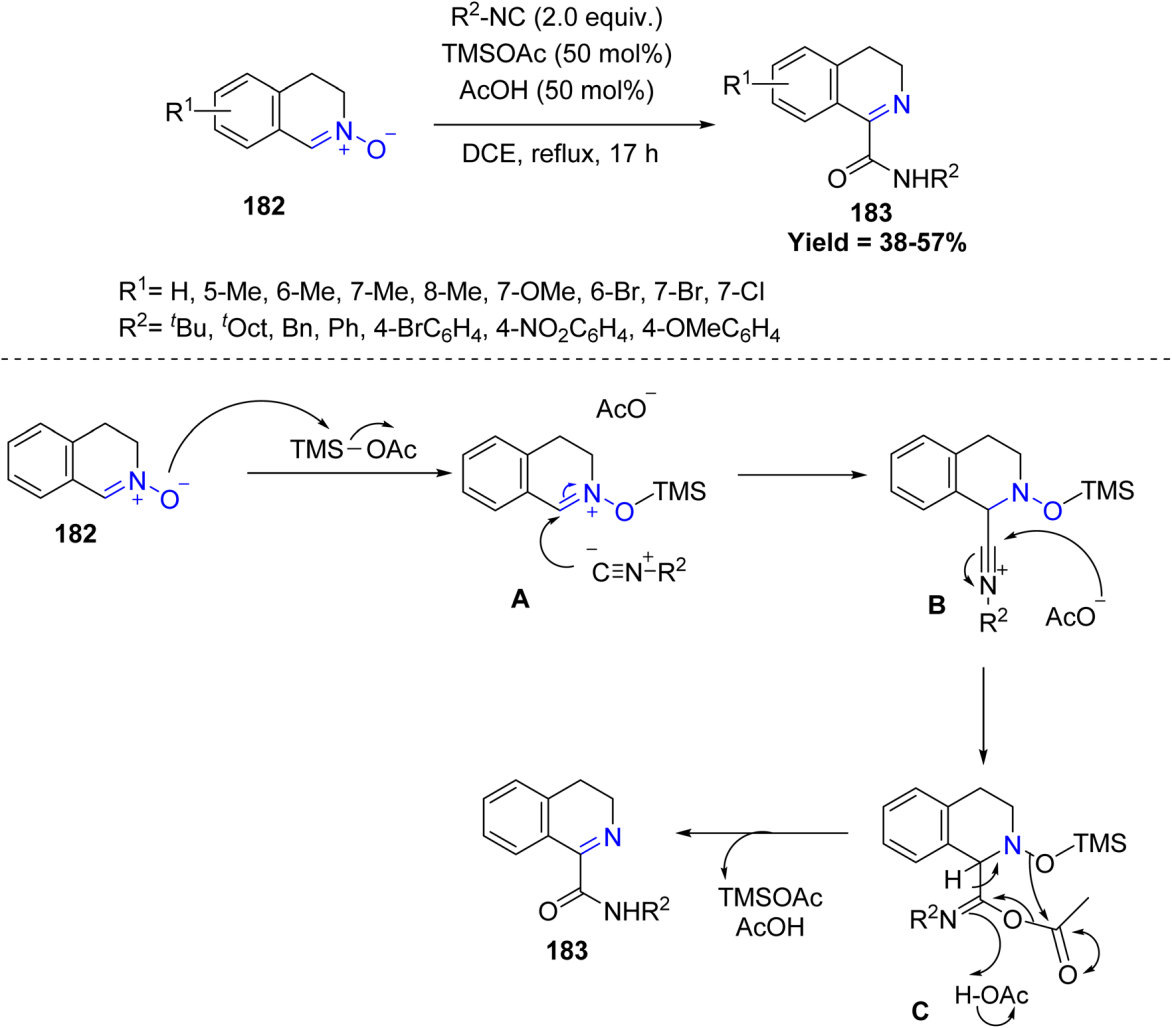
Silylacetate-promoted addition reaction of isocyanides to nitrones.

Takahiro Soeta *et al.*, in 2024 reported the addition of isocyanides to 3,4-dihydroisoquinoline-*N*-oxides 182*via* an innovative variation of the Ugi reaction to give the respective 3,4-dihydroisoquinoline-1-carboxylamide derivatives 183. Mechanistically the reaction is initiated by coordination of the nitrone oxygen with TMSOAc, generating intermediate A. This step is followed by stabilization of the resulting nitrilium intermediate *via* acetate addition, affording the imidoyl acetate C. Finally, regeneration of TMSOAc occurs concurrently with acyl group transfer, leading to the formation of the final product 183. Aliphatic isocyanides, particularly bulky tertiary and secondary alkyl groups, reacted efficiently, giving good to high yields, whereas aromatic isocyanides generally exhibited lower reactivity, with strongly electron-withdrawing substituents completely inhibiting the reaction. Limitations include reduced efficiency with electron-deficient or sterically hindered substrates, sensitivity to solvent choice, and lower reactivity for certain aromatic isocyanides, highlighting the need for optimized conditions for each substrate class (Scheme 47).^94^

## 3 Conclusion

Nitrones continue to represent a highly versatile class of intermediates in heterocyclic synthesis, enabling access to structurally diverse scaffolds with wide-ranging biological and material applications. Traditional transition-metal-catalyzed strategies have provided remarkable control over regio- and stereoselectivity, yet their high cost and limited substrate scope often restrict broader adoption. In parallel, the development of transition-metal-free methodologies has opened new opportunities for sustainable and scalable synthesis, particularly through [3 + 2], [2 + 2] and [4 + 2] cycloadditions, as well as emerging approaches such as asymmetric click chemistry, deoxygenative cyclization, Ugi reactions, photoredox catalysis, and self-oxidative cyclizations. Collectively, these methods showcase the adaptability of nitrone chemistry to diverse synthetic challenges while advancing the principles of green and economical synthesis. Despite these advances, significant challenges remain. The development of protocols that combine high selectivity, broad functional group tolerance, and operational simplicity under environmentally benign conditions is still a pressing need. In particular, catalyst-free strategies and photocatalytic approaches hold considerable promise for addressing issues of cost, scalability, and sustainability. Moreover, expanding the substrate scope to accommodate complex, multifunctional molecules will be vital for translating laboratory methodologies into practical applications. By addressing existing limitations and embracing interdisciplinary strategies, the field of nitrone-derived heterocycles is well-positioned to deliver transformative advances in both synthetic methodology and real-world applications. We believe this review will serve as a timely resource, offering insights that may inspire future innovations and guide researchers toward the development of more efficient, sustainable, and widely applicable nitrone-based transformations.

## Author contributions

Suhasini Mohapatra: conceptualization, writing original draft; Kamalika Prusty: review and editing; Subhashree Bhol: review and editing; Gopinatha Panigrahi: review and editing; Sabita Nayak: conceptualization, validation, supervision, funding acquisition, editing original draft.

## Conflicts of interest

There is no conflict of interest among the authors.

## Data Availability

No data was used for the research described in the article.
